# Promysalin is a salicylate-containing antimicrobial with a cell-membrane-disrupting mechanism of action on Gram-positive bacteria

**DOI:** 10.1038/s41598-017-07567-0

**Published:** 2017-08-18

**Authors:** Rahul D. Kaduskar, Giulia Della Scala, Zaaima J. H. Al Jabri, Stefania Arioli, Loana Musso, Marco R. Oggioni, Sabrina Dallavalle, Diego Mora

**Affiliations:** 1 Department of Food, Environmental, and Nutritional Sciences (DeFENS), University of Milan, via Celoria 2, 20133 Milan, Italy; 2 Department of Genetics, University of Leicester, University Road, Leicester, LE1 7RH United Kingdom

## Abstract

Promysalin was previously described as a narrow spectrum molecule with a unique species-specific activity against *Pseudomonas aeruginosa*. Here we demonstrate that promysalin is active against Gram-positive and Gram-negative bacteria using a microdilution assay. Promysalin acts on Gram-positive bacteria with a mechanism of action involving cell membrane damage with leakage of intracellular components. The evaluation of MICs and MBCs on 11 promysalin analogs, synthesized utilizing diverted total synthesis, allowed the identification of the structural moieties potentially involved in cell membrane interaction and damage. The mechanism of action of promysalin against Gram-negative bacteria is still not clarified, even if a synergistic effect with the bisguanidine chlorhexidine on cell membrane disruption has been observed.

## Introduction

The recent studies that highlight the long term effects on human physiology and health of the early exposure to antibiotics^1^, have prompted researchers to increase their efforts towards the search of new molecules with a narrow spectrum of activity. Antibiotics do not have species-specific activity, but are characterized by a wide spectrum of activity against several bacterial species and therefore they severely impact on microbiota composition^2^. It is well known that perturbations to the microbiota, such as those caused by the use of oral antibiotics, deplete the commensal microbiota, allowing pathogens to proliferate, thus determining gastrointestinal inflammations^3^. In this context, promysalin, a salicylate-containing *Pseudomonas putida* antibiotic, was described to selectively target members of the genus *Pseudomonas*, without affecting the viability of other Gram-negative and Gram-positive bacteria^4, 5^. More recently^6^, diverted total synthesis of promisalyn analogs demonstrates that the bioactivity of the molecule is sensitive to changes within its hydrogen bond network. Furthermore, it was reported that promysalin inhibits the production of the siderophore pyoverdine which is often linked to virulence. Therefore, the inhibition of pyoverdine by promysalin was considered an interesting point in order to develop novel antivirulence therapy^6^.

The amphiphilic nature of promysalin, prompted us to assume a mechanism of action based on cell membrane interaction. However, the interaction of promysalin with the membrane phospholipid bilayer was never investigated. The narrow spectrum of activity of promysalin against only bacteria of the genus *Pseudomonas* ^4^, was described by the authors as a novelty. To the best of our knowledge, there are no other molecules of microbial origin known to be active in a genus-specific way with the exception of a genus-specific molecule which was obtained modifying the original amino acid sequence of the antimicrobial peptide protegrin I through cycles of peptidomimetic synthesis^7^.

The basic purpose of the work outlined here was to investigate the activity of promysalin and analogues, prepared by a recently developed synthesis protocol^8^, against several Gram-negative and Gram-positive reference species using a validated standard microdilution method^9^. Additionally, a further aim was to get insights into the mechanism of action of promysalin.

## Results

### Promysalin is a broad spectrum antimicrobial

The data obtained (Table 1), highlight that promysalin is active against Gram-positive and Gram-negative bacteria including several human pathogens. The Minimal Inhibitory Concentrations measured (MICs) ranged from 16 µg/ml for *Pseudomonas aeruginosa* ATCC 10145 and ATCC 27853, *Staphylococcus aureus* ATCC 29213 and *Streptococcus thermophilus* DSM 20617 ^T^, to 256 µg/ml for all *Lactobacillus* species analyzed. MICs of 64 µg/ml were measured for *Enterococcus durans* NCDO 956, *E. faecalis* ATCC 29212, ATCC 19433, and LMG 19456, *E. faecium* ATCC 19434, *Staphylococcus eidermidis* ATCC 14990 ^T^ and *Streptococcus pyogenes* ATCC 12344 ^T^. Higher values were determined for the Minimal Bactericidal Concentrations (MBCs) that ranged from 32–64 µg/ml for *Bacillus subtilis* DSM 347, *S. thermophilus* DSM 20617 ^T^ and *S. pneumoniae*, to values of 256 or >256 µg/ml for all the other bacterial species analyzed. The overall data indicated that promysalin is sensibly more active on streptococci and lactococci than on lactobacilli. The activity of promysalin detected against Gram-positive microrganisms was in contrast to previous observation^4^ describing promysalin as an antibiotic which selectively targeted members of the genus *Pseudomonas*. The reason of these conflicting data is due to the methods used by Li and colleagues^4^ for the screening of promysalin sensitive bacteria, the agar diffusion test. In the SI (Figures S1–S3) we showed that agar and agar-well diffusion tests were not able to identify promysalin sensitive bacteria with exception of the sensitive *P. stutzeri* LMG 2333, whereas the dilution assay method was effective in the identification of promysalin-sensitive bacterial species. These contradictory results were not due to the different media used for the antimicrobial activity test. In fact TSB, the elective media for *P. stutzeri* LMG 2333, for *S. thermophilus* DSM20617^T^ and *Pediococcus acidilactici* PAC1.0 did not allow to visualize any promysalin activity against this strains in agar plate (Figure S2). Since the inhibitory activity of promysalin against the sensitive *Psuedomonas stutzeri* LMG 2333 was detectable using the agar diffusion assay, whereas it was not against the sensitive Gram-positive bacteria, we could hypothesize that promysalin might act on *Pseudomonas* spp. and on Gram-positive bacteria through a different mechanism of action.

**Table 1:** Minimal inhibitory concentration (MIC) and minimal bactericidal concentration (MBC) values of promysalin against Gram-negative and Gram-positive bacteria.

Bacterial species		MIC (µg/ml)	MBC (μg/ml)	Temperature (°C)
*Acetobacter aceti*	MIM2000/28	512	>512	30
*Escherichia coli*	ATCC 25922	64	256	37
*Pseudomonas aeruginosa*	ATCC 10145	16	128	30
*Pseudomonas aeruginosa*	ATCC 27853	16	128	30
*Pseudomonas stutzeri*	LMG 2333	512	>512	30
*Enterococcus durans*	NDCO 956	64	>256	37
*Enterococcus faecalis*	ATCC 29212	64	>256	37
*Enterococcus faecalis*	ATCC 19433	64	>256	37
*Enterococcus faecalis*	LMG 19456	64	>256	37
*Enterococcus faecium*	ATCC 19434	64	>256	37
*Enterococcus italicus*	DSM 15952	32	>256	37
*Lactococcus cremoris*	DSM 20069	32	32	30
*Lactococcus garviae*	DSM 20684	64	>256	30
*Staphylococcus aureus*	ATCC 25923	32	>256	37
*Staphylococcus aureus*	ATCC 29213	16	>256	37
*Staphylococcus epidermidis*	ATCC 14990 ^T^	64	128	37
*Streptococcus pneumoniae*	ATCC 700669	32	64	37
*Streptococcus pneumoniae*	ATCC 49619	32	64	37
*Streptococcus pneumoniae*	Pen6	32	64	37
*Streptococcus pyogenes*	ATCC 12344 ^T^	64	128	37
*Streptococcus thermophilus*	DSM 20617 ^T^	16	64	37
*Pediococcus acidilactici*	PAC1.0	128	128	37
*Lactobacillus acidophilus*	DSM 20079	256	>256	37
*Lactobacillus delbrueckii* subsp. *bulgaricus*	ATCC 11842	256	>256	37
*Lactobacillus casei*	LMG 6904	256	>256	37
*Lactobacillus helveticus*	ATCC 15009 ^T^	256	>256	37
*Lactobacillus paracasei* subsp. *paracasei*	DSM 5622 ^T^	256	>256	37
*Lactobacillus plantarum*	ATCC 4008	256	>256	30
*Bacillus subtilis*	DSM 347	16	32	30

### Promysalin acts on Gram-positive bacteria disrupting the cell membrane phospholipid bilayer

**Figure 1:**
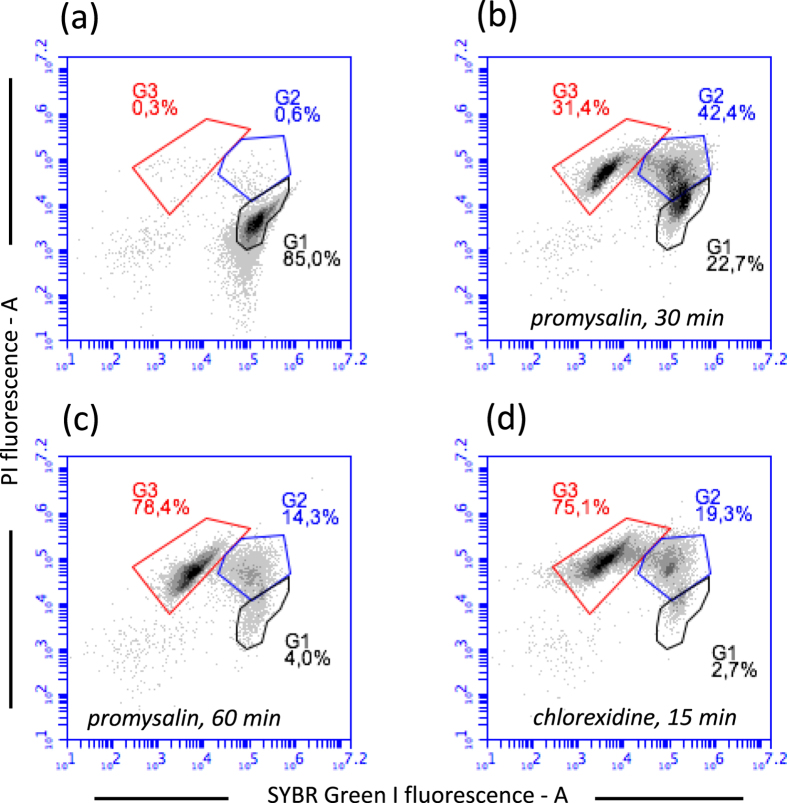
The effect of promysalin and chlorhexidine on *S. thermophilus* DSM 20617 ^T^ cell membrane integrity. Flow cytometry density diagrams show the SYBR Green I *vs* PI fluorescence of cells exposed to promysalin or chlorhexidine (100 µg/ml respectively). (**a**) Cells before the exposure to the antibacterial molecule. (**b**–**d**) Cells after the exposure to the antimicrobial molecule. Viable cells are gated in G1, viable cells with slightly damaged cell membrane are gated in G2. Dead cells with damaged membrane are gated in G3. The transition of cell population from gate G1 to gate G3 is related to the entity of cell membrane damage.

**Figure 2:**
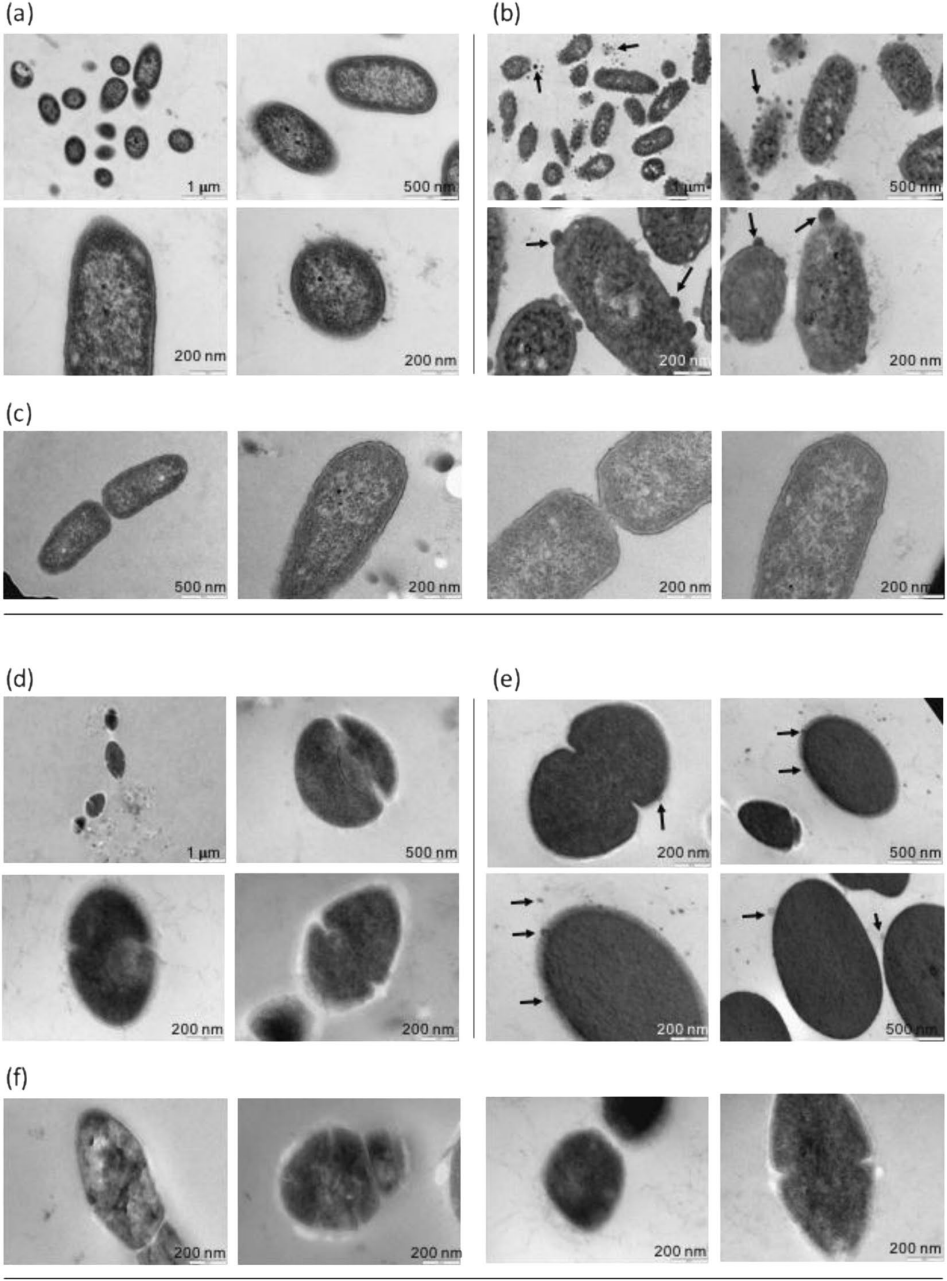
Transmission Electron Microscope images of *P. aeruginosa* ATCC 10145 and *S. thermophilus* DSM 20617 ^T^ before and after exposure to chlorhexidine and promysalin. (**a**) *P. aeruginosa* cell not exposed and (**b**) exposed to chlorhexidine (100 µg/ml) or (**c**) to promysalin (100 µg/ml). (**d**) *S. thermophilus* cell not exposed and (**e**) exposed to chlorhexidine (100 µg/ml) or (**f**) to promysalin (100 µg/ml). Black arrows indicate the membrane protrusions in cell exposed to chlorhexidine.

**Figure 3:**
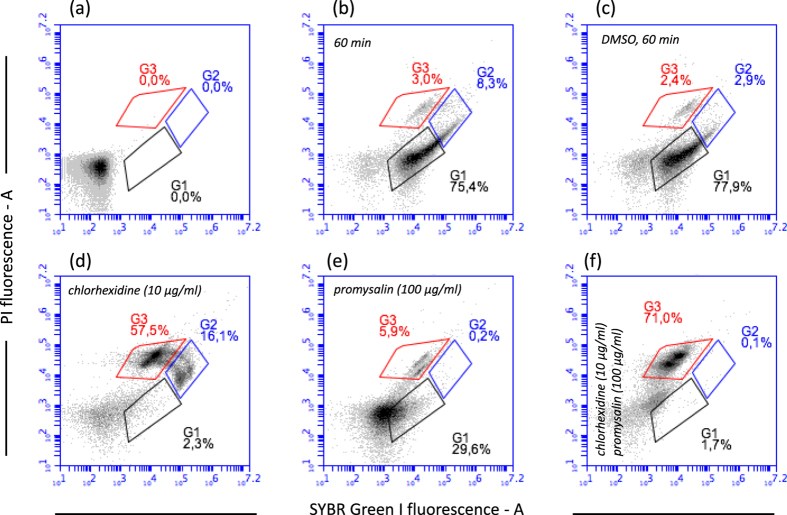
Membrane damage in *P. aeruginosa* ATCC 10145 exposed to promysalin, chlorhexidine, and to a mixture of the two molecules. Flow cytometry density diagrams show the SYBR Green I vs PI fluorescence of cells exposed to promysalin or chlorhexidine or both the molecules. Viable cells are gated in G1, viable cells with slightly damaged cell membrane are gated in G2. Dead cells with damaged membrane are gated in G3. The transition of cell population from gate G1 to gate G3 is related to the entity of cell membrane damage. (**a**) *P. aeruginosa* unlabeled cells. (**b**) *P. aeruginosa* cells incubated 60 min at 37 °C. (**c**) *P. aeruginosa* cells incubated 60 min at 37 °C in presence of DMSO. (**d**) *P. aeruginosa* cells incubated 60 min at 37 °C in presence of chlorhexidine (10 µg/ml). (**e**) *P. aeruginosa* cells incubated 60 min at 37 °C in presence of promysalin (100 µg/ml). (**F**) *P. aeruginosa* cells incubated 60 min at 37 °C in presence of chlorhexidine and promysalin (10 µg/ml and 100 µg/ml respectively). DMSO was added to cell suspension in the same amount which was present in the promysalin solution.

In order to obtain information on the mechanism of action of promysalin on Gram-positive bacteria, we exposed cell suspension to promysalin and then we evaluated the cell viability by flow cytometry. We used as model organisms the Gram-positive *S. thermophilus* DSM 20617 ^T^. Interestingly, cells exposed to 100 µg/ml of promysalin lost quickly viability (increasing their propidium iodide fluorescence) in the same way as when they were exposed to the biocide chlorhexidine (100 µg/ml) (Fig. 1), but with a different kinetic. Chlorhexidine determined the loss of viability of 75% of the cells population in 15 min while promysalin determined a similar effect after 60 min of exposure (Fig. 1). It is worth of mention that the loss of cell viability, by promysalin and chlorhexidine exposure, was determined by membrane damage as shown by the increased propidium iodide (PI) cell fluorescence, and by the decreased SYBR-Green I cell fluorescence. Further experiments, carried out on the viability of promysalin-exposed cells of other Gram-positive bacteria such as *Staphylococcus aureus* ATCC 25923, *L. paracasei* DSM 5622, and *B. subtilis* DSM 347 (SI, Figures S7–9) confirmed what previously observed for *S. thermophilus* cells. Chlorhexidine is an effective biocide known to be able to disrupt the cell membrane with a mechanism similar to antimicrobial peptides^10^. Benzalkonium chloride, another biocide belonging to the quaternary ammonium compounds (QACs) category, is a cationic surfactant whose mechanism of action implies the destruction of the lipid bilayer in the bacterial cell membrane^11^. Benzalkonium chloride, likewise chlorhexidine determined the same loss of membrane integrity in *S. thermophilus* and in the Gram-negative *Pseudomonas aeruginosa* ATCC 10145 (Figure S4). Chlorhexidine and benzalkonium chloride showed MICs and MBCs values against *S. thermophilus* lower than those measured for promysalin, whereas MICs and MBCs values were comparable to those measured for promysalin for *P. aeruginosa* ATCC 10145. We therefore hypothesized that chlorhexidine, benzalkonium chloride and promysalin share the same mechanisms of action. In this context, the amphipathic nature of promysalin is compatible with a possible interaction with the cell phospholipid bilayer. Unfortunately, when promysalin was tested against the sensitive *Pseudomonas stutzeri* LMG 2333 and *P. aeruginosa* ATCC 10145 by flow cytometry, a moderate or no cell membrane damage was observed, even if the exposition of bacterial cells to promysalin was prolonged for several hours at 37 °C (Figures S10–11). Transmission Electron Microscope analysis of *P. aeruginosa* and *S. thermophilus* cells exposed to promysalin did not show any visible membrane damage, whereas the exposition to chlorhexidine determined in *P. aeruginosa*, and with a lower frequency in *S. thermophilus*, the formation of vesicles and protrusions (Fig. 2). These vesicles are mainly described in Gram-negative^12^ and can have several functions in bacterial physiology, including the excretion of toxic compounds such as chlorhexidine^13^. TEM analysis thus confirmed that the mechanisms of action of promysalin and chlorhexidine were not comparable. However, the exposure of *P. aeruginosa* ATCC 10145 to promysalin together with a sub-lethal dose of chlorhexidine (10 µg/ml) showed an increase of membrane damage compared to that obtained exposing cells to high chlorhexidine concentration (Fig. 3). These results led us to hypothesize that promysalin cannot access the cell membrane of *P. aeruginosa*, unless chlorhexidine is present. Noteworthy, an evident membrane damage was generated by promysalin on the Gram-negative *Escherichia coli* and *Acetobacter aceti* (Figures S12 and S13), thus leading us to conclude that the outer membrane composition, or the cell surface structure of *Pseudomonas* species, interact with promysalin limiting its access to the phospholipid bilayer.

**Figure 4:**
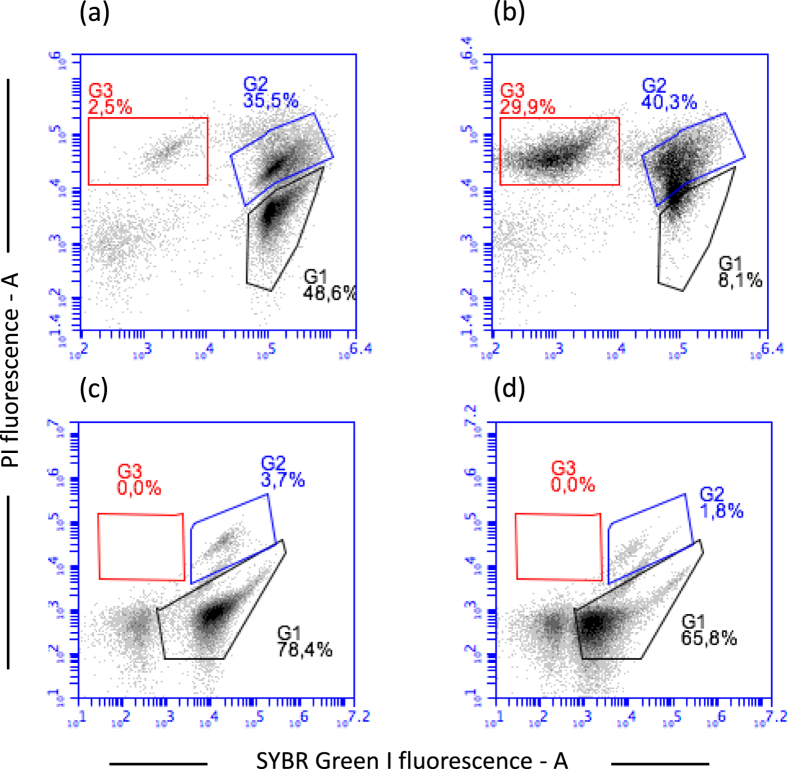
Membrane damage in *P. aeruginosa* ATCC 10145 and *S. thermophilus* DSM 20617 ^T^ exposed to surfactin. Flow cytometry density diagrams show the SYBR Green I vs PI fluorescence of cells exposed to promysalin or chlorhexidine or both the molecules. Viable cells are gated in G1, viable cells with slightly damaged cell membrane are gated in G2. Dead cells with damaged membrane are gated in G3. The transition of cell population from gate G1 to gate G3 is related to the entity of cell membrane damage. (**a**) *S. thermophilus* incubated 60 min at 37 °C in presence of DMSO. (**b**) *S. thermophilus* incubated 60 min at 37 °C in presence of surfactin (200 µg/ml). (**C**) *P. aeruginosa* cells incubated 60 min at 37 °C in presence of DMSO. (**d**) *P. aeruginosa* cells incubated 60 min at 37 °C in presence of surfactin (200 µg/ml). DMSO was added to cell suspension in the same amount which was present in the surfactin solution.

The amphiphilic nature of promysalin may allow an effect on bio-membranes similar to those exerted by microbial lipopetides such as surfactin^14^. Surfactin is a powerful biosurfactant able to penetrate into lipid membranes by means of hydrophobic interactions^15^. The effect of surfactin on the cell viability and the cell membrane integrity have been tested on *P. aeruginosa* ATCC 10145 and *S. thermophilus* DSM 20617 ^T^ and the results obtained were comparable to those previously reported for promysalin. While *S. thermophilus* showed a substantial membrane damage after 1 h of exposition to surfactin, no effect on cell membrane integrity was observed for *P. aeruginosa* (Fig. 4). MICs and MBCs for surfactin reflected the results obtained on membrane integrity, being *S. thermophilus* more sensitive (MIC 64 µg/ml, MBC 256 µg/ml) compared to *P. aeruginosa* (MBC 256 µg/ml, MBC >256 µg/ml). We therefore conclude that promysalin and surfactin might have the same mechanisms of action on cell membrane of Gram-positive bacteria.

### Promysalin is active against *Saccharomyces cerevisiae*

A partial cellular membrane damage, comparable to that obtained with the bisbiguanide biocide chlorhexidine, was also observed when cells of the yeast *Saccharomyces cerevisiae* BC1 were exposed to promysalin (Supporting Information, Figure S14). According to the partial cellular membrane damage reported above, a total inhibition of the growth of *Saccharomyces cerevisiae* BC1 was not observed (Figure S17), whereas a complete inhibition of the growth was observed when *S. cerevisae* was exposed to 8 µg/ml of chlorhexidine (Supporting Information, Figure S18), thus underlining that the mechanisms of the two molecules were different. At the highest concentration tested (128 µg/ml), promysalin determined a delay in the lag phase of the growth of more than 8 h compared to the growth in its absence. These data highlighted the potential activity of this molecule against yeast and fungi, thereby expanding the spectrum of activity of promysalin towards eukaryotic microorganisms.

### Promysalin cell-membrane damaging is accompanied with the release of intracellular material

**Figure 5:**
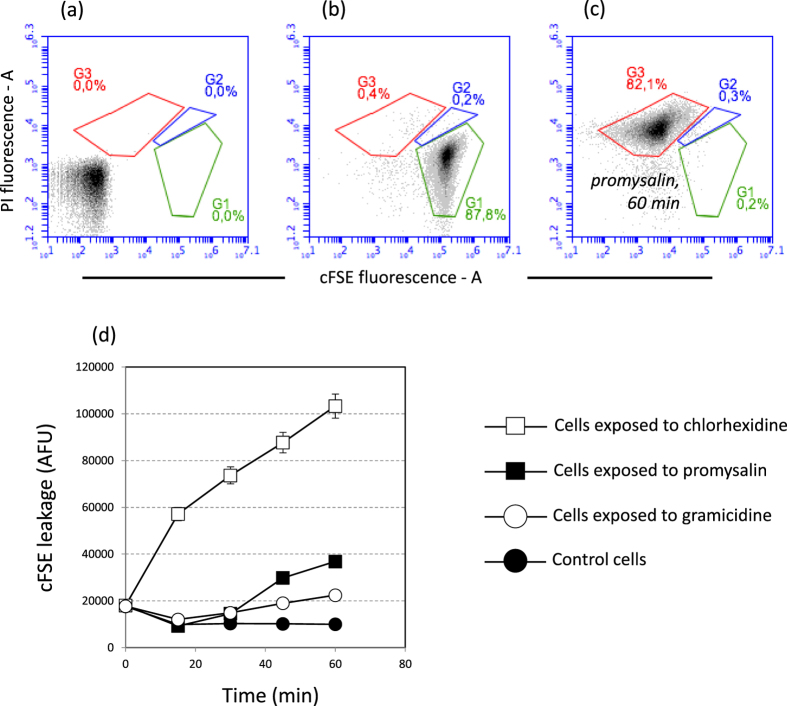
Membrane damage and cFSE fluorescence leakage in *S. thermophilus* DSM 20617 ^T^ exposed to promysalin, chlorhexidine, and gramicidin. Flow cytometry density diagrams show the cFSE *vs* PI fluorescence of cells exposed to promysalin or chlorhexidine or gramicidin (100, 200 µg/ml, and 100 µM respectively). (**a**) Cells before cFDASE labelling. (**b**) Cell labelled with cFSE and PI. (**c**) Cells after 60 min exposure to promysalin. Viable cells are gated in G1, viable cells with slightly damaged cell membrane are gated in G2. Dead cells with damaged membrane are gated in G3. The transition of cell population from gate G1 to gate G3 is related to the entity of cell membrane damage. (**d**) Leakage of cFSE fluorescence outside the cells during the exposure to promysalin, chlorhexidine and the membrane uncoupling gramicidin.

With the aim to verify if membrane damage was followed by the release of intracellular material, *S. thermophilus* DSM 20617 ^T^ cells were labelled with the fluorescence probe 5 (and 6-)-carboxyfuorescein succinimidyl ester (cFSE) using the cFDASE as a precursor molecule^16^. The membrane-permeating cFDASE is cleaved by intracellular esterases and the resultant cFSE molecules are conjugated to the aliphatic amines of intracellular proteins^17^. cFSE labelled *S. thermophilus* cells were exposed to promysalin or chlorhexidine and the cell membrane damage together with the leakage of cFSE fluorescence outside the cells was monitored by flow cytometry and by a standard fluorometer. As shown in Fig. 5, both promysalin, and with higher efficiency chlorhexidine, determined a reduction of cFSE fluorescence inside the cells and an increase of cFSE fluorescence outside the cells, thus leading us to hypothesize that phospholipid bilayer disruption was accompanied by the release of intracellular components.

These results imply that the mechanism of action of promysalin is linked to the disruption of the phospholipid membrane bilayer rather than the increase in ions membrane permeability as it happens for the membrane uncoupling gramicidin^18^. Noteworthy, gramicidin was not effective in creating a membrane damage or a cFSE-fluorescence leakage in *S. thermophilus* DSM 20617 ^T^ (Fig. 5 and SI Figure S5).

### Chemical synthesis of promysalin derivatives revealed that the salicylate fragment, the dehydroproline moiety, and the myristamide chain are confirmed mandatory to maintain the cell-membrane damage

**Figure 6:**
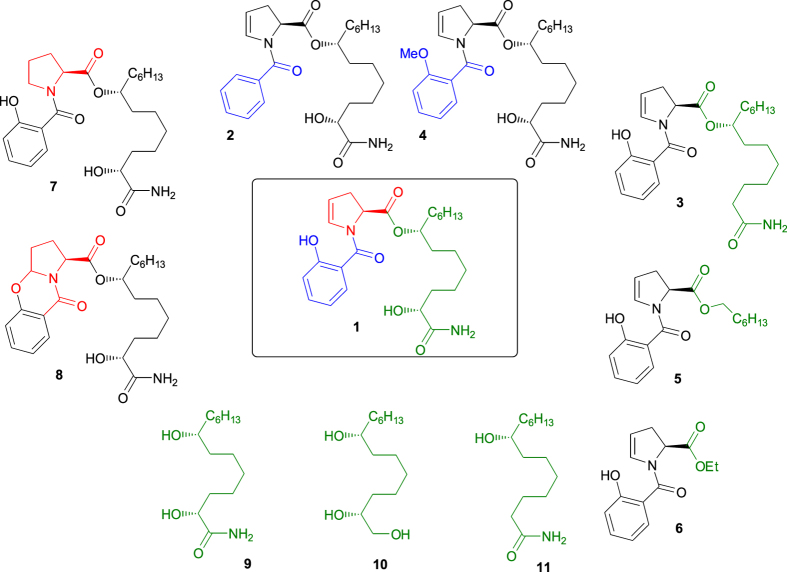
Structure of promysalin (**1**) and analogs (**2**–**11**).

The hypothesis on promysalin mechanisms of action formulated above, prompted us to gain insights into the most relevant structural features affecting the damage of the cellular phospholipid bilayer. We recently reported a total synthesis of promysalin^8^. The convergent character of our synthetic route made possible its easy application for the preparation of some representative derivatives. Our strategy to modulate the activity focused on stepwise modifications in three distinct areas of the molecule: the salicylate fragment, the dehydroproline moiety and the myristamide chain. To elucidate the role of the salicylate fragment, we synthesized analogues **2** and **4**, whereas compounds **3**, **5**, **6** were prepared to test the importance of the chain linked to the dehydroproline system. We also synthesized compounds **9**–**11** to assess whether the myristamide chain alone could maintain antimicrobial activity. Concerning the heterocyclic ring, we introduced a proline fragment in place of the enamide moiety (compound 7). As we noticed that promysalin was quite unstable under acidic conditions^8^, we also synthesized the cyclized analogue, containing a more rigid tetrahydro-9-oxa-aza-cyclopentanaphthalen-4-one ring (compound **8**), by treatment with TFA. All analogs are shown in Fig. 6. The synthetic route to all these compounds is reported in SI.

The antibacterial efficacy of promysalin analogs was firstly checked by microdilution methods in order to established MIC and MBC values against the following reference Gram-negative and Gram-positive strains: *Pseudomonas aeruginosa* ATCC 10145, *Staphylococcus aureus* ATCC 29213, and *S. thermophilus* DSM 20617 ^T^. MICs and MBCs values are reported in Table 2. Interestingly, MIC values comparable, although higher than those measured for promysalin, were retained by the analogs **2**, **3**, **4**, **7** and **8**. MBC values below 256 µg/ml were measured only for *S. thermophilus* strain and for analogs **2**, **3**, **4**, **7** and **8**, thus suggesting that for the other species promysalin and the analogs had a bacteriostatic rather than a bactericidal effect. Due to the high MIC values measured for analogs **9**–**11**, we conclude that the salicylate fragment and the dehydroproline moiety are essential for the antibacterial activity of promysalin. It is also interesting to note that analog **8**, in contrast to previous observation of Steele and colleagues^6^, retained a significant activity against *Pseudomonas aeruginosa* ATCC 10145, *Staphylococcus aureus* ATCC 29213, and *S. thermophilus* DSM 20617 ^T^. Analog **5** and **6** showed a significant activity reduction especially when tested against the Gram-positive *Staphylococcus aureus* ATCC 29213, and *S. thermophilus* DSM 20617 ^T^ (Table 2), thus confirming the relevance of the dehydroproline moiety for the antibacterial activity of promysalin.

**Table 2:** Minimal inhibitory concentration (MIC, μg/ml) and minimal bactericidal concentration (MBC, μg/ml) values of promysalin and analogues against Gram-negative and Gram-positive bacteria.

Compound	*Pseudomonas aeruginosa* ATCC 10145		*Staphylococcus aureus* ATCC 29213		*Streptococcus thermophilus* DSM20617^T^	
	MIC	MBC	MIC	MBC	MIC	MBC
Promysalin	16	128	16	>256	16	64
2	128	>256	64	>256	64	128
3	32	512	32	>256	32	64
4	32	>256	32	>256	64	128
5	32	>256	>256	>256	128	>256
6	128	>256	>256	>256	256	>256
7	32	>256	>256	>256	64	128
8	128	>256	64	>256	64	64
9	128	>256	125	>256	128	>256
10	128	>256	>256	>256	128	>256
11	128	>256	>256	>256	128	>256

**Figure 7:**
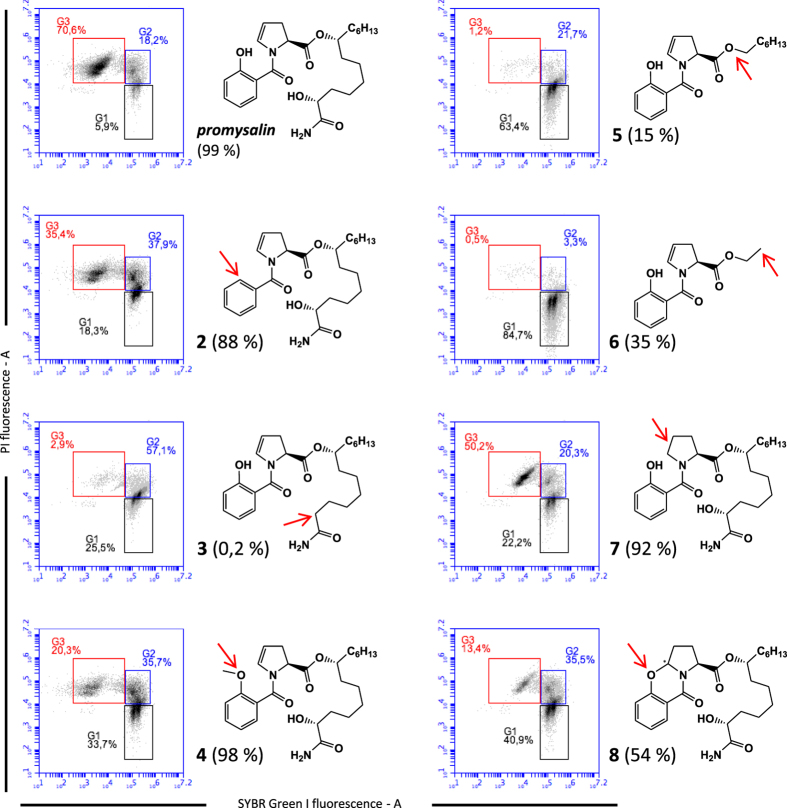
The effect of promysalin and the derivative analogs on *S. thermophilus* DSM 20617 ^T^ cell membrane integrity and cell viability. Flow cytometry density diagrams show the SYBR Green I *vs* PI fluorescence of cells exposed to promysalin or to the analogs **2–8** (100 µg/ml). Viable cells are gated in G1, viable cells with slightly damaged cell membrane are gated in G2. Dead cells with damaged membrane are gated in G3. The transition of cell population from gate G1 to gate G3 is related to the entity of cell membrane damage. The reduction of cell viability measured by standard plating was shown in parenthesis. The reduction of cell viability was calculated using as reference (100% of viability reduction) the bactericidal effect of chlorhexidine. The differences in structure between promysalin and the analogs are indicated by the red arrows. Flow cytometry density diagrams showing the SYBR Green I *vs* PI fluorescence of cells exposed to chlorhexidine or DMSO are shown in SI, Figure S5.

To evaluate the role on cell membrane damage of the structural modifications, we tested the analogs that retained MIC values equal or lower than 128 µg/ml in *S. thermophilus* DSM 20617 ^T^ by flow cytometry using the SYBR Green I/PI double staining assay. In addition, the cell suspensions exposed to promysalin or to the analogs, were subjected to the evaluation of cell viability by standard plating. Very interestingly, we observed that the benzoyl enamide (**2**) and the methyl ether (**4**) retained a remarkable membrane damage activity and a remarkable reduction of viability (88% and 98%, respectively) (Fig. 7). The cyclized compound (**8**) retained an interesting reduction of viability (54%), but it showed a strong reduction of the ability to interfere with the membrane probably as a consequence of the acquired structural rigidity. The fully functionalized myristamide chain was confirmed mandatory to maintain the activity, and the lipophilic ester derivatives (**5**, **6**) determined a remarkable reduction of membrane damage confirmed by a weak reduction of cell viability (15% and 35%, respectively). The C-2 dehydroxylation (**3**) was found to be detrimental, resulting in a negligible cell viability reduction despite the moderate cell membrane damage observed. Nevertheless, the exposure of *S. thermophilus* to compound **3** showed the highest % of cells with slightly damaged cell membrane (gate G2 in Fig. 7) thus suggesting that a prolonged exposure to this compound could increase the % of dead cells. The MIC and MBC values of compound **3** were in fact comparable to those of promysalin. More interestingly, the proline derivative (**7**) retained most of the membrane damage activity and cell viability reduction. To summarize, each portion of the parent molecule seemed to play an important role, only the modification of salicylate fragment being tolerated.

## Discussion

Promysalin, a salicylate-containing antimicrobial, previously characterized for its specific activity against members of the genus *Pseudomonas* by agar diffusion assay^4^, showed a wide spectrum of activity when tested by a standard microdilution method for drug susceptibility testing^9^. Using the microdilution method, promysalin showed to be active against Gram-negative and Gram-positive bacteria with comparable MIC values. Exposing cell suspensions to promysalin, we were able to detect by PI staining a damage of the cell membrane which was clearly evident in all Gram-positive strains tested, and some Gram-negative strains. However, a moderate or no membrane damage was detectable respectively in *P. stutzeri* LMG 2333 and in *P. aeruginosa* ATCC 10145, used as representative strains of Gram-negative bacteria, even if they were both sensitive to promysalin^4^ (Table 1). These differences in the role exerted by promysalin on cell membrane of Gram-positive and on bacteria belonging to the genus *Pseudomonas* led to hypothesize that promysalin could act on this genus using different mechanisms of action.

In light of the data presented here, the disruption of phospholipid bilayer appears as the most plausible mechanism of action of promysalin on Gram-positive and on non-*Pseudomonas* Gram-negative bacteria, while other possible mechanisms, already hypothesized^6^, can be responsible for the activity of promysalin on the genus *Pseudomonas*. In this context, the outer membrane of Gram-negative bacteria composed of an interior leaflet of phospholipids and an exterior leaflet of lipopolysaccharide, could represent in *Psedomonas* species a barrier which limits the access of promysalin to the below cell membrane phospholipid bilayer. The induction of outer membrane vesicles by chlorhexidine, with a consequent increase of the outer membrane overall surface, allowed promysalin to access and damage the cell membrane also in the Gram-negative *P. aeruginosa* ATCC10145.

We therefore conclude that a mechanism of action that involved cell membrane damage justifies the wide spectrum of activity shown by promysalin (Table 1) against several Gram-positive bacteria and Gram-negative bacteria. A stepwise chemical modification of the three distinct areas of the molecule (the salicylate fragment, the dehydroproline moiety and the myristamide chain) clearly highlighted that the dehydroproline moiety is essential for the antibacterial activity of promysalin and for its interaction with the membrane phospholipid bilayer.

The overall data described in this study are shedding new light on the spectrum of activity and mechanism of action of promysalin. The broad-spectrum activity shown against Gram-positive and Gram-negative bacteria, together with the ability of promysalin to interfere only with the membrane phospholipid bilayer, suggest to further investigate the mechanism of action of this molecule on Gram-negative bacteria with focus on the genus *Pseudomonas*. Therefore, the use of promysalin to counteract human infections, such as those caused by *Pseudomonas aeruginosa* as hypothesized in a previous study^6^, should be carefully evaluated.

## Methods

### Promysalin and its derivatives chemical synthesis

Promysalin synthesis was carried out as described previously^8^. Promysalin derivatives were synthesized according to the procedures described in the Supporting Information. Promysalin and its derivatives were stored dried at −20 °C and they were solved in DMSO at a final concentration of 4 mg/ml (Sigma-Aldrich, Milan, Italy) prior to be used in biological assays.

### Microbiological media and culture condition

Bacteria strains and yeast were maintained at −80 °C and cultivated in the appropriate media as reported in the Supplementary Information.

### Evaluation of minimal inhibitory concentration (MIC) and minimal bactericidal concentration (MBC) of promysalin, its derivative analogues and other compounds against Gram-positive and Gram-negative bacteria

Minimum inhibitory concentration (MIC) was determined using the standard microdilution method for drug susceptibility testing^8^. The detailed procedure is described in the Supplementary Information.

### Flow cytometry evaluation of cell membrane damage and measurement of cFSE fluorescence cell leakage

To evaluate whether membrane damage was linked to cell leakage of intracellular components, microbial cells grown for 18 h in the appropriate medium in Petri dishes were collected and diluted in sterile filtered (0.2 µm) phosphate-buffered saline (PBS) (NaCl 8 g/L; KCl 0.2 g/L; Na_2_HPO_4_ 1,44 g/L; KH_2_PO_4_ 0.24 g/L; pH 7.4) to a final concentration of 10^8^ events per ml. The cell suspension was diluted to 10^6^ events/ml and then exposed to promysalin (100 µg/ml) or its derivative analogues (100 µg/ml), chlorhexidine (100 µg/ml) (Sigma-Aldrich) or benzalkonium chloride (100 µg/ml) (Sigma-Aldrich) at 37 °C. The cell suspension was also exposed to a DMSO control. At the time requested, a sample was collected and subjected to SYBR Green I/PI double staining and analysis by flow cytometry and, when necessary, to a standard plate count in the appropriate medium. The detailed flow cytometry protocol is reported in the Supplementary Information. To evaluate whether membrane damage was linked to cell leakage of intracellular components, microbial cells grown for 18 h in the appropriate medium in Petri dishes were collected and diluted in PBS to a final concentration of 10^8^ per ml. The obtained cell suspension was supplemented with 4 µM cFDASE (Sigma-Aldrich, Milan, Italy), which is a precursor molecule of cFSE. The cFSE fluorescence intensity of stained cells was recovered by flow cytometry in the FL1 channel (excitation 488 nm, emission filter 530/30, provided by BD Biosciences, Milan, Italy). The cFSE-labeled cell suspension was then exposed to promysalin (100 µg/ml) or its derivative analogues (100 µg/ml), chlorhexidine (100 µg/ml) (Sigma-Aldrich) or benzalkonium chloride (100 µg/ml) (Sigma-Aldrich) at 37 °C. As a control, the cFSE-labeled cell suspension was also exposed to a volume of DMSO solvent equal to that used for promysalin and its derivative analogues. At the time point, two samples of each cell suspension were collected: i) one sample was labeled with PI as described above, incubated at room temperature for 15 min, and analyzed by flow cytometry. The second sample was used to measure cFSE-fluorescence cell leakage. In this case the sample was centrifuged (13000 rpm, 2 min), and the cell-free supernatant transferred to a 96-microtiter plate for measurement of cFSE-fluorescence in a Victor 3 fluorometer (PerkinElmer). The fluorescence data were calculated as the average of three independent assays and expressed in arbitrary units of fluorescence ± the standard deviation. Further details are described in the Supporting Information.

## Electronic supplementary material

Supplementary  Information
