# Supplementary material for: Promysalin is a salicylate-containing antimicrobial with a cell-membrane-disrupting mechanism of action on Gram-positive bacteria
Source: Sci Rep. 2017 Aug 18;7:8861. doi: 10.1038/s41598-017-07567-0 (PMC5562885; doi:10.1038/s41598-017-07567-0)
Supplement: Supplementary file 1 — Supplementary Information [file 41598_2017_7567_MOESM1_ESM.pdf]

Promysalin is a salicylate-containing antimicrobial with a cell-membrane-disrupting mechanism of action on Gram-positive bacteria

Rahul D. Kaduskar[1], Giulia Della Scala[1], Zaaima J.H. Al Jabri[2], Stefania Arioli[1], Loana Musso[1], Marco R. Oggioni[2], Sabrina Dallavalle[1], Diego Mora[1*]

[1]Department of Food, Environmental, and Nutritional Sciences (DeFENS), University of Milan, via Celoria 2, 20133 Milan, Italy

[2] Department of Genetics, University of Leicester, University Road, Leicester LE1 7RH, United Kingdom

Supporting information

Table of contents

1) Synthesis

1.1 General information S3

1.2 General procedures S4

1.3 Experimental procedures S7

1.3.1 Synthesis of compound **2** S7

1.3.2 Synthesis of compound **3** S10

1.3.3 Synthesis of compound **4** S11

1.3.4 Synthesis of compound **5** S13

1.3.5 Synthesis of compound **6** S14

1.3.6 Synthesis of compound **7** S15

1.3.7 Synthesis of compound **8** S18

1.3.8 Synthesis of compound **9** S19

1.3.9 Synthesis of compound **10** S20

1.3.10 Synthesis of compound **11** S21

1.4. References S23

1.5. Spectral data S24

2) Material and methods related to microbiological assays

2.1. Microbiological media and culture condition S57

2.2. Agar and agar-well diffusion assay S57
2.3. Evaluation of Minimal Inhibitory Concentration (MBC) and Minimal Bactericidal Concentration (MBC) of promysalin and its derivative analogs against Gram-positive and Gram-negative bacteria S59
2.4. Flow cytometry evaluation of cell membrane damage and measurement of cFSE-fluorescence cell leakage S61
2.5. Evaluation of the growth-kinetic parameters in *S. cerevisiae* in absence and in presence of promysalin S76

Tables

Table S1 S60

Figures

Figure S1 S58
Figure S2 S58
Figure S3 S58
Figure S4 S63
Figure S5 S64
Figure S6 S65
Figure S7 S66
Figure S8 S67
Figure S9 S68
Figure S10 S69
Figure S11 S70
Figure S12 S71
Figure S13 S72
Figure S14 S73
Figure S15 S74
Figure S16 S75
Figure S17 S76
Figure S18 S77

1) Synthesis

1.1 General information

All reagents and solvents were reagent grade or were purified by standard methods before use. Melting points were determined in open capillaries by a SMP3 apparatus and are uncorrected. ^{1}H spectra were recorded on Bruker AMX 300 MHz and Bruker AV600 spectrometers. TMS was used as an internal standard and the chemical shifts were reported in parts per million (δ). The peak patterns are indicated as follows: s, singlet; d, doublet; dd, doublet of doublet; t, triplet; m, multiplet; q, quartet. The coupling constants, J are reported in Hertz (Hz) and ^{13}C NMR spectra were recorded on Bruker AMX 300 MHz and Bruker AV600 spectrometers. Optical rotations were measured with a Perkin Elmer 241 polarimeter. The elemental analyses were recorded with a CARLO ERBA EA 1108 instrument. The accurate mass spectra were recorded using Bruker Daltonics model Autoflex III, accurate mass MALDI TOF/TOF MS/MS. Solvents were routinely distilled prior to use; anhydrous tetrahydrofuran (THF) and ether (Et_2O) were obtained by distillation from sodium-benzophenone ketyl; dry methylene chloride was obtained by distillation from phosphorus pentoxide. All reactions requiring anhydrous conditions were performed under a positive nitrogen flow and all glassware were oven dried and/or flame dried. Isolation and purification of the compounds were performed by flash column chromatography on silica gel 60 (230-400 mesh). Analytical thin-layer chromatography (TLC) was conducted on TLC plates (silica gel 60 F_{254}, aluminum foil). Compounds on TLC plates were detected under UV light at 254 and 365 nm or were revealed spraying with 10% phosphomolybdenic acid (PMA) in ethanol.

1.2 General procedures

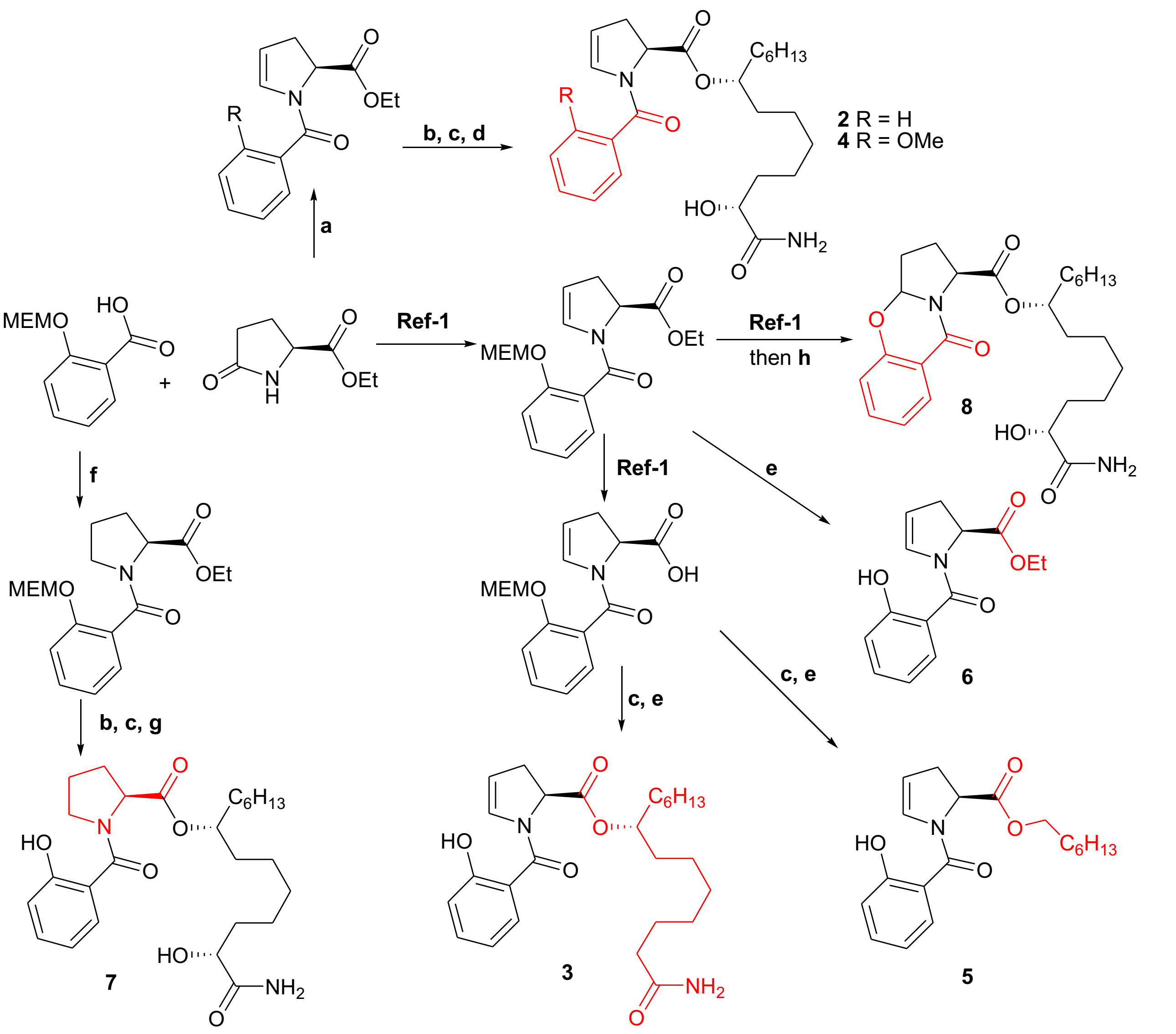

Scheme S1: Synthetic routes to compounds 2-8: a) i. aroyl chloride, NEt_3, 0 ºC to 80 ºC, 3h, ii. $LiBHEt_3$, toluene, -78 ºC, 1 h, then DIPEA, cat. DMAP, TFAA, -78 ºC to rt, 3 h; b) LiOH, EtOH : H_2O, 0 ºC to rt, 5 h; c) i) 2,4,6-trichlorobenzoyl chloride, NEt_3, THF, 0 ºC to rt, 2h, ii) alcohol, DMAP, toluene, 0 ºC to rt, 12 h; d) TBAF, THF, 0 ºC to rt, 1h; e) $TiCl_4$, -20 ºC, 15 min.; f) L-proline ethyl ester.HCl, EDCI, HOBt; g) 1N HCl, THF, 0 ºC to rt; h) TFA, CH_2Cl_2, rt, 30 min.

General procedure A: acylation of ethyl L-pyroglutamate.

NEt_3 (2 eq.), followed by acid chloride (1.2 eq.) were added dropwise to a stirred solution of ethyl L-pyroglutamate (1 eq.) in toluene (0.5 M) at 0 °C under N_2 atmosphere. The mixture was stirred at 80 °C for 3 h and cooled to room temperature. Sat. $NaHCO_3$ was added and the organic layer was separated. The aqueous layer was extracted with EtOAc (× 2). The combined organic extracts were washed with brine, dried over anhydrous Na_2SO_4 and concentrated *in vacuo*. The product was purified using flash column chromatography in 0-30% EtOAc/hexane.

General procedure B: reductive elimination.

To a stirred solution of acylated pyroglutamate (1 eq.) in dry toluene (0.2 M) was added Superhydride® (lithium triethylborohydride) (1.2 eq., 1M in THF) at -78 °C under N_2 atmosphere. The mixture was stirred at -78 °C for 1h, then DMAP (0.1 eq.) and DIPEA (5.7 eq.) were added, followed by very slow addition of TFAA (1.2 eq.). The reaction mixture was gradually warmed to room temperature and stirred for 3 h. Water (× 10) was added and the organic layer was separated. The aqueous layer was extracted with ethyl acetate (× 2); the combined organic extracts were washed with brine, dried over anhydrous Na_2SO_4 and concentrated *in vacuo*. The residue was purified using flash column chromatography in 0-50 % ethyl acetate: hexane.

General procedure C: hydrolysis of ethyl ester.

To a solution of ethyl ester (1 eq.) was added dropwise a solution of LiOH (1.5 eq.) in water (EtOH : H_2O 2:1, 0.08 M) at 0 °C. The reaction mixture was warmed to room temperature and stirred for 5 h. EtOH was removed *in vacuo,* the aqueous layer was washed with 40 % ethyl acetate in diethyl ether (× 2), cooled to 0 °C and acidified using 5% citric acid. The product was extracted using 5% CH_3OH : CH_2Cl_2 (× 3). The combined organic extracts were washed with brine, dried over anhydrous Na_2SO_4 and concentrated *in vacuo* to afford carboxylic acid.

General procedure D: Yamagouchi esterification.

NEt_3 (3 eq.) followed by 2,4,6-trichlorobenzoyl chloride (2 eq.) were added dropwise to a stirred solution of acid (1eq.) in THF (0.03 M) at 0 °C under N_2 atmosphere. The mixture was warmed to room temperature and stirred for 2h. THF was removed *in vacuo* and the residue was dissolved in toluene (0.03M). DMAP (3 eq.) followed by alcohol (0.8 eq.) in toluene were added at 0 °C under N_2 atmosphere. The resulting suspension was stirred overnight at room temperature. EtOAc (× 15) was added, the organic layer was washed with sat. NH_4Cl, brine, dried over anhydrous Na_2SO_4 and concentrated *in vacuo*. The product was purified by flash column chromatography 0-70 % EtOAc: hexane.

General procedure E: MEM deprotection.

To a stirred solution of MEM ether (1 eq.) in CH_2Cl_2 (0.15 M) was added $TiCl_4$ (2 eq., 1M in CH_2Cl_2) at -20 °C under N_2 atmosphere. The reaction mixture was stirred at -20 °C for 10 min; then aqueous

ammonia (20 times) was added. The aqueous layer was extracted with ethyl acetate (×2), and the combined organic extracts were washed with brine and dried over anhydrous Na_2SO_4. The solvent was removed *in vacuo*. The product was purified using preparative TLC.

General procedure F: TBDPS deprotection.

TBAF (3 eq. 1M in THF) was added dropwise to a stirred solution of silyl ether (1 eq.) in THF (0.2 M) at 0 °C. The reaction mixture was stirred at room temperature for 1h. Sat. NH_4Cl was added. The aqueous layer was extracted with ethyl acetate (× 2). The combined organic extracts were washed with brine, dried over anhydrous Na_2SO_4 and concentrated *in vacuo*. The product was purified using preparative TLC.

1.3 Experimental procedures

Promysalin (compound **1**) was synthesized as described in the literature.[1]

1.3.1 Synthesis of compound 2 (1-Benzoyl-2,3-dihydro-1H-pyrrole-2-carboxylic acid 7-carbamoyl-1-hexyl-7-hydroxy-heptyl ester)

Ethyl (2S)-1-benzoyl-2,3-dihydro-1H-pyrrole-2-carboxylate (S2)

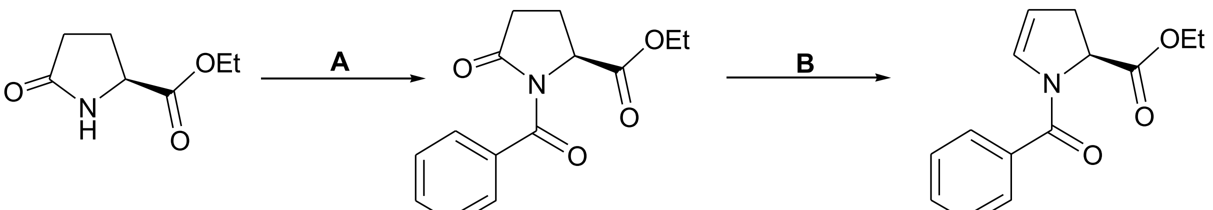

Using general procedure **A,** ethyl L-pyroglutamate (500 mg, 3.28 mmol) yielded ethyl (S)-1-benzoyl-5-oxopyrrolidine-2-carboxylate (**S1**) as a creamy solid (620 mg, 74 %); R_f (25 % EtOAc: Hexane) = 0.25, $[\alpha]_D^{23}$ = + 23.0 (*c* 1.00, $CHCl_3$). mp = 79-80 °C. ^{1}H NMR (300 MHz, $CDCl_3$) δ: 7.70-7.63 (2H, m), 7.58-7.50 (1H, m), 7.46-7.37 (2H, m), 4.89 (1H, dd, *J* = 3.9, 8.9 Hz), 4.26 (2H, q, J = 7.1 Hz), 2.83-2.68 (1H, m), 2.66-2.38 (1H, m), 2.23-2.09 (1H, m), 1.30 (3H, t, J = 7.1 Hz). ^{13}C NMR (75 MHz, $CDCl_3$) δ: 173.7, 171.2, 170.6, 134.0, 132.5, 129.3 (×2), 128.1 (×2), 62.1, 59.0, 32.0, 22.1, 14.3. Anal. Calcd. for $C_{14}H_{15}NO_4$: C, 64.36; H, 5.79; N, 5.36. Found: 64.25; H, 5.78; N, 5.37.

Using general procedure **B** the ester **S1** (478 mg, 1.83 mmol) yielded title compound **S2** as a colourless oil (200 mg, 44 %)**;** R_f (30 % EtOAc: Hexane) = 0.4; $[\alpha]_D^{23}$ = - 138.6 (*c* 1.00, $CHCl_3$).

^{1}H NMR (600 MHz, acetone-*d*$_6$) δ: 7.60-7.31 (5H, m); 6.57 (1H, s); 5.17 (1H, s); 4.95 (1H, dd, *J* = 5.1, 11.7 Hz); 4.28-4.12 (2H, m); 3.20-3.12 (1H, m); 2.70-2.63 (1H, m); 1.35-1.19 (3H, m). ^{13}C NMR (150 MHz, acetone-*d*$_6$) δ: 170.5, 166.0, 135.6, 130.6, 130.4, 128.4 (× 2), 127.6 (× 2), 108.4, 60.6, 58.4, 33.4, 13.5. Anal. Calcd. for $C_{14}H_{15}NO_3$: C, 68.56; H, 6.16; N, 5.71. Found: C, 68.64; H, 6.15; N, 5.72.

(7R,13R)-14-amino-13-hydroxy-14-oxotetradecan-7-yl (S)-1-benzoyl-2,3-dihydro-1H-pyrrole-2-carboxylate (2)

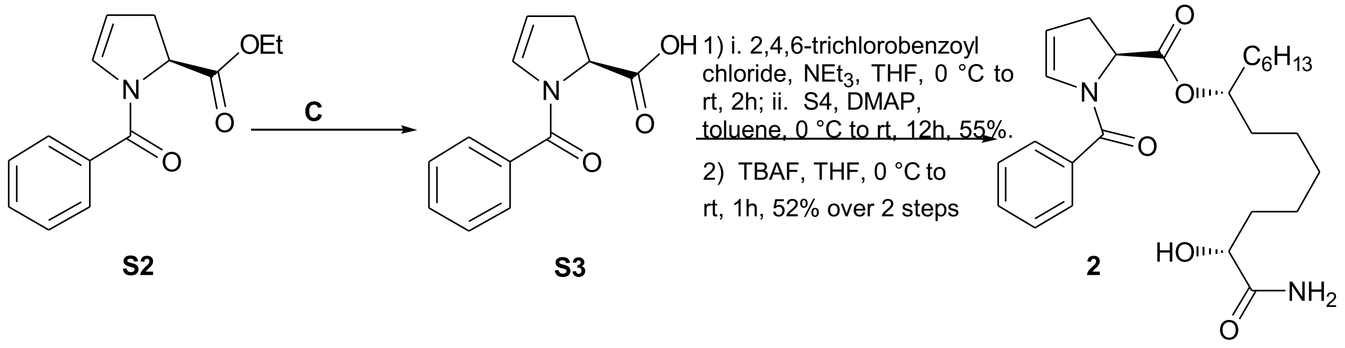

Using general procedure **C** the ester **S2** (170 mg, 0.69 mmol) was hydrolyzed to give (S)-1-benzoyl-2,3-dihydro-1H-pyrrole-2-carboxylic acid (**S3**) as a pale yellow solid (130 mg). R_f (5 % MeOH : CH_2Cl_2) = 0.2. $[\alpha]_D^{23}$ = - 85.3 (*c* 1.20, $CHCl_3$). ^{1}H NMR (300 MHz, CD_3OD) δ: 7.62-7.55 (2H, m); 7.52-7.42 (3H, m); 6.51-6.47 (1H, m); 5.26-5.21 (1H, m); 4.84 (1H, dd, J = 4.5, 11.2 Hz); 3.18-3.05 (1H, m); 2.78-2.67 (1H, m); ^{13}C NMR (150 MHz, CD_3OD) δ: 177.3, 168.0, 135.4, 130.7, 130.2, 130.0 (× 2), 128.4, 127.66 (× 2), 111.2, 60.6, 34.2, 26.5. Compound **S3** was used for the next step without further purification.

NEt_3 (0.09 mL, 0.69 mmol) followed by 2,4,6-trichlorobenzoyl chloride (0.07 mL, 0.46 mmol) were added dropwise to a stirred solution of the above acid **S3** (50 mg, 0.23 mmol) in THF (4.1 mL, 0.03 M) at 0 °C under N_2 atmosphere. The mixture was warmed to room temperature and stirred for 2h. THF was removed *in vacuo* and the residue was dissolved in toluene (3 mL). DMAP (84 mg, 0.69 mmol) followed by (2R,8R)-2-((tert-butyldiphenylsilyl)oxy)-8-hydroxytetradecanamide (**S4**)[1] (57 mg, 0.115 mmol) in toluene were added at 0 °C under N_2 atmosphere. The resulting suspension was stirred overnight at room temperature. EtOAc (8 mL) was added, the organic layer was washed with sat. NH_4Cl (5 mL), brine (5 mL), dried over anhydrous Na_2SO_4 and concentrated *in vacuo*. The crude silyl ether (54 mg, 0.076 mmol) was dissolved in THF (1 mL) and cooled to 0 °C; TBAF (0.22 mL, 0.23 mmol, 1M in THF) was added dropwise under N_2 atmosphere. The reaction mixture was stirred at room temperature for 1 h. Sat. NH_4Cl (7 mL) was added and the aqueous layer was extracted with ethyl acetate (2 × 7 mL) The combined organic extracts were washed with brine (5 mL), dried over anhydrous Na_2SO_4 and concentrated *in vacuo*. The product was purified preparative TLC in 6% MeOH : CH_2Cl_2, to afford the title compound **2** as a colourless oil (28 mg, 52 % over two steps). R_f (5 % MeOH: CH_2Cl_2) = 0.4. $[\alpha]_D^{23}$ = -42.0 (*c* 0.45, $CHCl_3$). ^{1}H NMR (300 MHz, $CDCl_3$) δ: 7.57-7.36 (5H, m); 6.85-6.72 (1H, m); 6.53-6.46 (1H, m); 5.20-5.14 (1H, m); 5.13-5.01 (1H, m); 4.97 (1H, dd, J = 4.1, 12.1 Hz); 4.27 (1H, m); 4.11-4.02 (1H, m); 3.50 (1H, brs); 3.21-3.07 (1H, m); 2.77-2.64 (1H, m); 1.90-1.17 (20H, m); 0.88 (3H, t, J = 6.9 Hz); ^{13}C NMR (75 MHz, $CDCl_3$) δ: 177.6, 170.7, 167.9, 134.8, 131.1, 130.9, 128.8 (× 2), 127.8 (× 2), 110.0, 75.3, 70.7, 58.7, 34.9, 34.2, 34.1, 33.7, 31.9, 29.3, 27.6, 25.7, 24.8, 24.3, 22.8, 14.3. Anal.

Calcd. for $C_{26}H_{38}N_2O_5$: C, 68.10; H, 8.35; N, 6.11. Found: C, 68.01; H, 8.33; N, 6.12. HRMS**:** (ES+) calculated for $C_{26}H_{38}N_2O_5Na$ $(M + Na)^+$ 481.26729, Found: 481.26861.

1.3.2. **Synthesis of compound 3. (R)-14-amino-14-oxotetradecan-7-yl (S)-1-(2-hydroxybenzoyl)-2,3-dihydro-1H-pyrrole-2-carboxylate.**

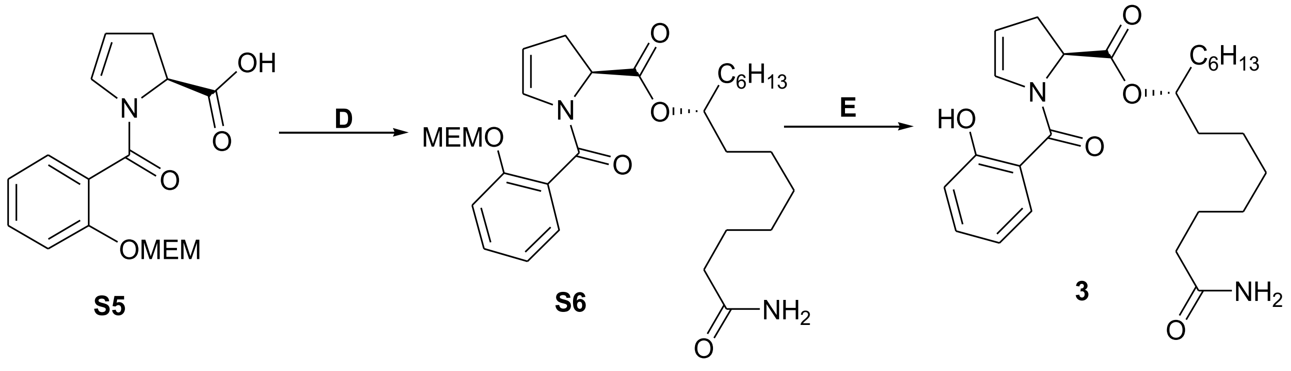

Using general procedure **D**, (S)-1-(2-((2-methoxyethoxy)methoxy)benzoyl)-2,3-dihydro-1H-pyrrole-2-carboxylic acid **S5** (50 mg, 0.15 mmol)[1] was reacted with (R)-8-hydroxytetradecanamide (**11**, see below) (32 mg, 0.139 mmol) to give (R)-14-amino-14-oxotetradecan-7-yl (S)-1-(2-((2-methoxyethoxy)methoxy)benzoyl)-2,3-dihydro-1H-pyrrole-2-carboxylate **S6** (40 mg, 54%) as a colourless oil. R_f (4 % MeOH:CH_2Cl_2) = 0.3. $[\alpha]_D^{23}$ = -54.5 (*c* 0.75, $CHCl_3$). ^{1}H NMR (300 MHz, $CDCl_3$) δ: 7.41-7.31 (2H, m); 7.22 (1H, dd, *J* = 8.2, 1.0 Hz); 6.30 (1H, brs); 6.21-6.14 (1H, m); 5.28 (2H, s); 5.09-4.91 (3H, m); 3.84-3.78 (2H, m); 3.57-3.50 (2H, m); 3.36 (3H, s); 3.20-3.07 (1H, m); 2.73-2.63 (1H, m); 2.42-2.34 (1H, m); 2.19 (2H, t, *J* = 7.5 Hz); 1.72-1.18 (20H, m); 0.87 (3H, t, *J* = 6.7 Hz); ^{13}C NMR (75 MHz, $CDCl_3$) δ = 176.3, 170.8, 165.3, 153.6, 131.6, 131.0, 128.9, 125.9, 122.4, 115.6, 109.0, 94.0, 75.5, 71.7, 68.2, 59.2, 58.2, 36.1, 34.7, 34.4, 31.9, 29.4, 28.9, 28.6, 25.6, 25.2, 24.8, 22.8, 14.3. Anal. Calcd. for $C_{30}H_{46}N_2O_7$: C, 65.91; H, 8.48; N, 5.12. Found: C, 65.83; H, 8.50; N, 5.10.

Using general procedure **E**, compound **S6** (85 mg, 0.159 mmol) was deprotected to afford the title compound **3** as a colourless oil (38 mg, 52 %). R_f (2 % MeOH:CH_2Cl_2) = 0.4. $[\alpha]_D^{23}$ = -50.1 (*c* 1.5, $CHCl_3$). ^{1}H NMR (300 MHz, $CDCl_3$) δ: 7.45-7.33 (2H, m); 6.99 (1H, dd, *J* = 8.2, 1.0 Hz); 6.89 (1H, ddd, *J* = 8.2, 8.2, 1.0 Hz); 6.79 (1H, brs); 5.67 (1H, brs); 5.32-5.16 (2H, m); 5.09-4.91 (2H, m); 3.23-3.05 (1H, m); 2.76-2.62 (1H, m); 2.20 (2H, t, *J* = 7.6 Hz); 1.74-1.11 (20H, m); 0.86 (3H, t, *J* = 6.9 Hz). ^{13}C NMR (75 MHz, $CDCl_3$) δ = 176.1, 171.1, 167.5, 158.8, 133.5, 131.1, 128.5, 119.2, 118.1, 117.5, 110.8, 76.1, 59.6, 36.1, 34.4, 34.2, 31.9, 29.3, 29.1, 29.0, 25.5 (× 3), 25.0, 22.7, 14.3. Anal. Calcd. for $C_{26}H_{38}N_2O_5$: C, 68.10; H, 8.35; N, 6.11. Found: C, 68.02; H, 8.37; N, 6.11. HRMS**:** (ES+) calculated for $C_{26}H_{38}N_2O_5Na$ $(M + Na)^+$ 481.26729, Found: 481.26829.

1.3.3. Synthesis of compound 4. (7R,13R)-14-amino-13-hydroxy-14-oxotetradecan-7-yl (S)-1-(2-methoxybenzoyl)-2,3-dihydro-1H-pyrrole-2-carboxylate

Ethyl (S)-1-(2-methoxybenzoyl)-5-oxopyrrolidine-2-carboxylate (S7)

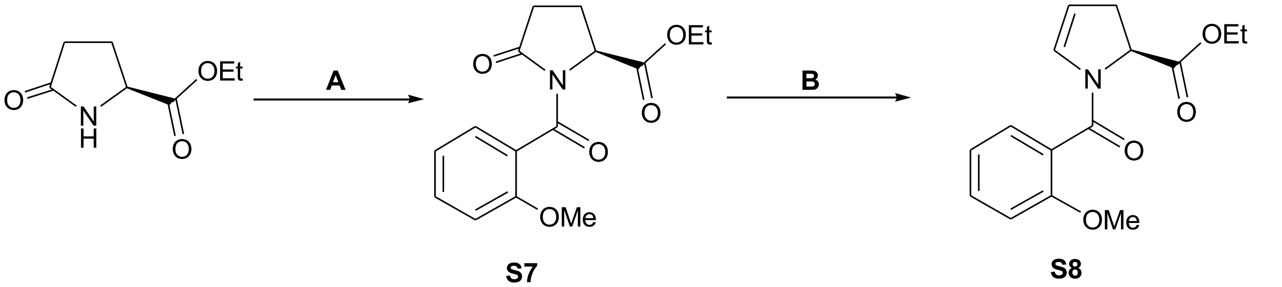

Using general procedure **A,** ethyl L-pyroglutamate (500 mg, 3.28 mmol) afforded compound **S7** as a white solid (723 mg, 77 %); R_f (30 % EtOAc: Hexane) = 0.4. mp = 93-94 °C. $[\alpha]_D^{23}$ = + 65.5 (*c* 1.00, $CHCl_3$). ^{1}H NMR (600 MHz, $CDCl_3$) δ: 7.44 (1H, ddd, *J* = 8.4, 7.7, 1.7 Hz); 7.33 (1H, dd, *J* = 7.7, 1.7 Hz); 7.01 (1H, dd, *J* = 7.7, 7.7 Hz); 6.93 (1H, d, *J* = 8.4 Hz); 4.92 (1H, dd, *J* = 9.5, 2.8 Hz), 4.29 (2H, q, *J* = 6.9 Hz); 3.83 (3H, s); 2.73-2.66 (1H, m); 2.57-2.52 (1H, m); 2.47-2.40 (1H, m); 2.19-2.13 (1H, m); 1.34 (3H, t, *J* = 7.1 Hz). ^{13}C NMR (150 MHz, $CDCl_3$) δ: 173.1, 171.4, 168.3, 157.2, 132.3, 129.0, 125.3, 120.8, 111.3, 62.1, 58.6, 56.1, 53.8, 31.9, 22.1, 14.5. Anal. Calcd. for $C_{15}H_{17}NO_5$ C, 61.85; H, 5.88; N, 4.81. Found: C, 61.95; H, 5.89; N, 4.80.

Using general procedure **B**, ethyl (S)-1-(2-methoxybenzoyl)-5-oxopyrrolidine-2-carboxylate **S7** (723 mg, 2.48 mmol) afforded compound **S8** as a clear oil (405 mg, 60 %). R_f (30 % EtOAc: Hexane) = 0.5. $[\alpha]_D^{23}$ = - 117.8 (*c* 1.25, $CHCl_3$). ^{1}H NMR (300 MHz, $CDCl_3$) δ = 7.43-7.33 (2H, m); 7.00 (1H, ddd, J = 8.2, 8.2, 1.1 Hz); 6.94 (1H, d, J = 8.2 Hz); 6.18-6.12 (1H, m); 5.08-5.03 (1H, m); 5.00 (1H, dd, J = 5.02, 11.7 Hz); 4.34-4.19 (2H, m); 3.84 (3H, s); 3.20-3.05 (1H, m); 2.76-2.65 (1H, m); 1.32 (3H, t, J = 7.1 Hz);^{13}C NMR (75 MHz, $CDCl_3$) δ = 171.2, 165.3, 156.1, 131.5, 131.0, 129.2, 125.1, 121.0, 111.6, 108.7, 61.6, 58.2, 56.0, 34.3, 14.3. Anal. Calcd. for $C_{15}H_{17}NO_4$: C, 65.44; H, 6.22; N, 5.09. Found: C, 65.54; H, 6.21; N, 5.08.

(7R,13R)-14-amino-13-hydroxy-14-oxotetradecan-7-yl (S)-1-(2-methoxybenzoyl)-2,3-dihydro-1H-pyrrole-2-carboxylate (4)

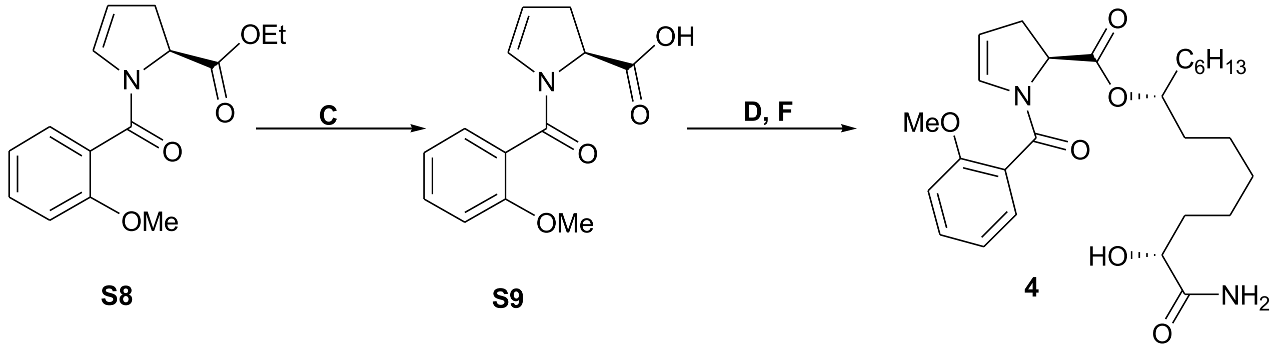

Using general procedure **C**, compound **S8** (250 mg, 0.908 mmol) was hydrolyzed to yield **S9** ((S)-1-(2-methoxybenzoyl)-2,3-dihydro-1H-pyrrole-2-carboxylic acid) as a yellow solid (185 mg, 82 %). R_f (4 % MeOH : CH_2Cl_2) = 0.4. mp = 145-146 °C. $[\alpha]_D^{23}$ = -82.8 (*c* 0.5, $CHCl_3$). ^{1}H NMR (300 MHz, $CDCl_3$) δ: 7.47-7.35 (2H, m); 7.03 (1H, ddd, *J* = 8.2, 8.2, 1.0 Hz); 6.96 (1H, d, *J* = 8.2 Hz); 6.07-6.02 (1H, m); 5.26-5.20 (1H, m); 5.13 (1H, dd, *J* = 4.3, 10.8 Hz); 3.84 (3H, s); 3.28-3.16 (1H, m); 3.11-2.96 (1H, m). ^{13}C NMR (75 MHz, $CDCl_3$) δ: 173.1, 167.3, 156.0, 132.2, 130.0, 129.3, 124.0, 121.1, 111.6, 111.3, 59.1, 56.0, 33.2. Anal. Calcd. for $C_{13}H_{13}NO_4$: C, 63.15; H, 5.30; N, 5.67. Found: C, 63.23; H, 5.29; N, 5.66.

Using general procedure **D**, the above acid **S9** (35 mg, 0.14 mmol) was reacted with **S4** ((2R,8R)-2-((tert-butyldiphenylsilyl)oxy)-8-hydroxytetradecanamide)[1] (56 mg, 0.112 mmol) to give (7R,13R)-14-amino-13-((tert-butyldiphenylsilyl)oxy)-14-oxotetradecan-7-yl (S)-1-(2-methoxybenzoyl)-2,3-dihydro-1H-pyrrole-2-carboxylate (**S10**) as a pale yellow oil (42 mg, 50 %) R_f (50 % EtOAc: hexane) = 0.3. $[\alpha]_D^{23}$ = -39.0 (*c* 0.4, $CHCl_3$). ^{1}H NMR (600 MHz, $CDCl_3$) δ: 7.67 (2H, d, J = 7.0 Hz); 7.62 (2H, d, J = 7.0 Hz); 7.50-7.34 (8H, m); 7.00 (1H, dd, J = 7.0, 7.0 Hz); 6.95 (1H, d, J = 8.3 Hz); 6.74 (1H, d, J = 4.5 Hz); 6.18-6.14 (1H, m); 5.58-5.54 (1H, m); 5.06-4.92 (3H, m); 4.27 (1H, dd, J = 3.9, 5.2 Hz); 3.85 (3H, s); 3.17-3.10 (1H, m); 2.71-2.66 (1H, m); 1.70-1.12 (29H, m); 0.98 (3H, t, J = 7.1 Hz); ^{13}C NMR (150 MHz, $CDCl_3$) δ: 176.2, 170.8, 164.9, 155.9, 135.7(× 2), 135.6(× 2), 133.0, 132.6, 131.2, 130.9, 130.2, 130.1, 127.9 (× 2), 127.8(× 2), 125.1, 120.8, 111.3, 108.3, 75.4, 74.2, 58.1, 55.7, 34.3, 34.2, 33.9, 33.8, 31.7, 29.7, 29.3, 29.2, 27.0 (× 2), 25.2, 24.8, 23.3, 22.6, 19.3, 14.0.

Compound **S10** (30 mg, 0.041 mmol) was deprotected using general procedure **F** to give the title compound **4** as a colourless oil (19 mg, 99 %). R_f (4 % MeOH : CH_2Cl_2) = 0.3, $[\alpha]_D^{23}$ = -32.8 (*c* 0.5, $CHCl_3$). ^{1}H NMR (300 MHz, $CDCl_3$) δ: 7.44-7.37 (1H, m); 7.33 (1H, dd, J = 1.8, 7.6 Hz); 7.03-6.97 (1H, m); 6.95 (1H, d, *J* = 8.2 Hz); 6.91 (1H, brs); 6.18-6.12 (1H, m); 5.14-4.99 (3H, m); 4.96 (1H, dd, *J* = 4.5, 11.6 Hz); 4.36 (1H, d, *J* = 5.4 Hz); 4.11-4.01 (1H, m); 3.83 (3H, s); 3.21-3.07 (1H, m); 2.73-2.62 (1H, m); 1.91-1.17 (20H, m); 0.88 (3H, t, *J* = 6.7 Hz). ^{13}C NMR (75 MHz, $CDCl_3$) δ: 177.9, 170.7, 166.0, 156.0, 131.9, 130.8, 129.0, 124.3, 121.1, 111.6, 109.8, 75.1, 70.5, 58.2, 56.0, 35.0, 34.3, 33.7, 31.9, 29.9, 29.3, 27.5, 25.7, 24.8, 24.4, 22.8, 14.3. Anal. Calcd. for $C_{27}H_{40}N_2O_6$: C, 66.37; H, 8.25; N, 5.73. Found: C, 66.21; H, 8.26; N, 5.72. HRMS**:** (ES+) calculated for $C_{27}H_{40}N_2O_6Na$ $(M + Na)^+$ 511.27786, Found: 511.28881.

1.3.4. Synthesis of compound 5. Heptyl (S)-1-(2-hydroxybenzoyl)-2,3-dihydro-1H-pyrrole-2-carboxylate

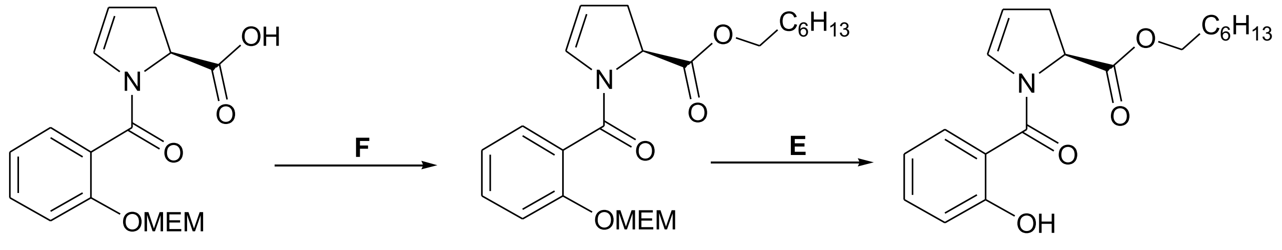

S5 **S11** **5**

Using general procedure **F**, compound **S5** (S)-1-(2-((2-methoxyethoxy)methoxy)benzoyl)-2,3-dihydro-1H-pyrrole-2-carboxylic acid [1] (50 mg, 0.15 mmol) was reacted with heptanol (0.026 mL, 0.19 mmol) to give heptyl (S)-1-(2-((2-methoxyethoxy)methoxy)benzoyl)-2,3-dihydro-1H-pyrrole-2-carboxylate **S11** as a colourless oil (50 mg, 58 %). R_f (30 % EtOAc: hexane) = 0.2. $[\alpha]_D^{23}$ = -85.7 (*c* 1.25, $CHCl_3$). ^{1}H NMR (300 MHz, $CDCl_3$) δ: 7.39-7.32 (2H, m); 7.21 (1H, dd, *J* = 1.2, 8.2 Hz); 7.04 (1H, ddd, *J* = 1.2, 8.2, 8.2 Hz); 6.18-6.13 (1H, m); 5.28 (2H, s); 5.05-5.01 (1H, m); 4.99 (1H, dd, *J* = 5.0, 11.8 Hz); 4.23-4.12 (2H, m); 3.83-3.76 (2H, m); 3.56-3.48 (2H, m); 3.35 (3H, s); 3.19-3.03 (1H, m); 2.74-2.63 (1H, m); 1.71-1.60 (2H, m); 1.42-1.18 (8H, m); 0.86 (3H, t, *J* = 6.7 H). ^{13}C NMR (75 MHz, $CDCl_3$) δ: 171.2, 165.1, 153.7, 131.4, 131.0, 129.1, 126.1, 122.3, 115.5, 108.7, 93.9, 71.7, 68.1, 65.8, 59.2, 58.1, 34.4, 31.9, 29.1, 28.8, 26.0, 22.8, 14.3. Anal. Calcd. for $C_{23}H_{33}NO_6$ C, 65.85; H, 7.93; N, 3.34. Found: C, 65.73; H, 7.91; N, 3.33.

Using general procedure **E** the above MEM ether **S11** (25 mg, 0.04 mmol) was deprotected to give the title compound **5** as a colourless oil (14 mg, 99 %). R_f (20 % EtOAc: hexane) = 0.4. $[\alpha]_D^{23}$ = -112.7 (*c* 0.65, $CHCl_3$). ^{1}H NMR (300 MHz, $CDCl_3$) δ: 9.78 (1H, s); 7.45-7.33 (2H, m); 7.01 (1H, dd, J = 1.2, 8.3 Hz); 6.88 (1H, ddd, J = 1.2, 8.3, 8.3 Hz); 6.84-6.80 (1H, m); 5.31-5.23 (1H, m); 5.03 (1H, dd, *J* = 5.3, 11.4 Hz); 4.27-4.09 (2H, m); 3.20-3.04 (1H, m); 2.78-2.67 (1H, m); 1.72-1.61 (2H, m); 1.40-1.16 (8H, m); 0.87 (3H, t, *J* = 7.1 Hz); ^{13}C NMR (75 MHz, $CDCl_3$) δ: 170.9, 167.6, 159.2, 134.1, 130.9, 128.3, 118.8, 118.0, 116.8, 110.6, 65.8, 59.3, 33.5, 31.7, 28.8, 28.5, 25.7, 22.5, 14.0. Anal. Calcd. for $C_{19}H_{25}NO_4$: C, 68.86; H, 7,60; N, 4.23. Found: C, 68.68; H, 7,58; N, 4.24. HRMS**:** (ES+) calculated for $C_{19}H_{25}NO_4Na$ $(M + Na)^+$ 354.16758, Found: 354.16798.

1.3.5. Synthesis of compound 6. Ethyl (S)-1-(2-hydroxybenzoyl)-2,3-dihydro-1H-pyrrole-2-carboxylate

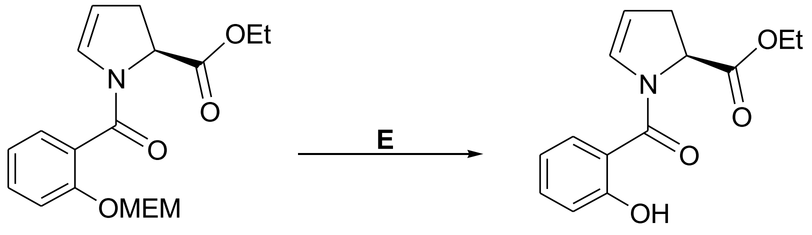

S12 **6**

Using general procedure **E** ethyl (S)-1-(2-((2-methoxyethoxy)methoxy)benzoyl)-5-oxopyrrolidine-2-carboxylate[1] (**S12)** (35 mg, 0.10 mmol) was deprotected to give the title compound **6** as a colourless oil (20 mg, 76 %), R_f (20 % EtOAc : hexane) = 0.3; $[\alpha]_D^{23}$ = - 132.3 (*c* 1.00, $CHCl_3$). ^{1}H NMR (300 MHz, $CDCl_3$) δ: 9.76 (1H, s); 7.46-7.34 (2H, m); 7.00 (1H, dd, *J* = 1.2, 8.3 Hz); 6.88 (1H, ddd, *J* = 8.3, 8.3, 1.2 Hz); 6.84-6.78 (1H, m); 5.30-5.24 (1H, m); 5.02 (1H, dd, *J* = 5.2, 11.4 Hz); 4.30-4.19 (2H, m); 3.18-3.05 (1H, m); 2.78-2.66 (1H, m); 1.29 (3H, t, J = 7.1 Hz). ^{13}C NMR (75 MHz, $CDCl_3$) δ: 171.1, 167.8, 159.3, 133.7, 131.1, 128.5, 119.1, 118.2, 117.0, 110.9, 61.9, 59.5, 33.6, 14.3. Anal. Calcd. for $C_{14}H_{15}NO_4$: C, 64.36; H, 5.79, N, 5.36. Found: C, 64.52; H, 5.78; N, 5.34. HRMS**:** (ES+) calculated for $C_{14}H_{15}NO_4Na$ $(M + Na)^+$ 284.08933, Found: 284.08971.

1.3.6. Synthesis of compound 7. (1-(2-hydroxybenzoyl)-pyrrolidine-2-carboxylic acid 7-carbamoyl-1-hexyl-7-hydroxyheptyl ester)

Ethyl (2-((2-methoxyethoxy)methoxy)benzoyl)-L-prolinate (S13).

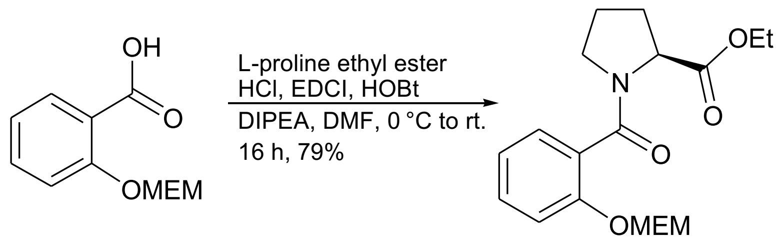

HOBt (125 mg, 0.93 mmol) and EDC·HCl (168 mg, 0.93 mmol) were added sequentially to a stirred solution of 2-((2-methoxyethoxy)methoxy)benzoic acid[1] (150 mg, 0.66 mmol) and L-proline ethyl ester·HCl (143 mg, 0.80 mmol) in DMF (2 mL, 0.3 M) at 0 °C under N_2 atmosphere. DIPEA (0.54 mL, 3.31 mmol) was added dropwise and the reaction mixture was stirred at room temperature for 16 h. The reaction mixture was poured into ice cold water (15 mL) and the aqueous layer was extracted with ethyl acetate (2 × 10 mL). The combined organic extracts were washed with cold brine (2 × 5 mL), dried over anhydrous Na_2SO_4 and concentrated *in vacuo*. Purification using flash column chromatography in 2 % MeOH : CH_2Cl_2 afforded the compound **S13** as a colourless oil (185 mg, 79 %). R_f (2 % EtOAc: hexane) = 0.15. $[\alpha]_D^{23}$ = - 69.7 (*c* 1.2, $CHCl_3$). ^1H NMR (300 MHz, $CDCl_3$) mixture of rotamers (major) δ: 7.34-7.12 (3H, m); 7.07-7.00 (1H, m); 5.29 (2H, s); 4.65 (1H, dd, J = 4.3, 8.6 Hz); 4.23 (2H, q, J = 6.9 Hz); 3.86-3.76 (2H, m); 3.57-3.51 (2H, m); 3.36 (3H, s); 3.38-3.25 (2H, m); 2.37-1.78 (4H, m); 1.31 (3H, t, J = 6.9 Hz). ^{13}C NMR (75 MHz, $CDCl_3$) δ: (major) 172.2, 168.0, 153.1, 130.2, 128.0, 127.7, 122.2, 115.4, 94.0, 71.5, 67.9, 61.0, 59.0, 58.6, 48.2, 29.6, 24.7, 14.2. Anal. Calcd. for $C_{18}H_{25}NO_6$: C, 61.52; H, 7.17; N, 3.99. Found: C, 61.39; H, 7.16; N, 4.00.

(7R,13R)-14-amino-13-hydroxy-14-oxotetradecan-7-yl (2-hydroxybenzoyl)-L-prolinate (7)

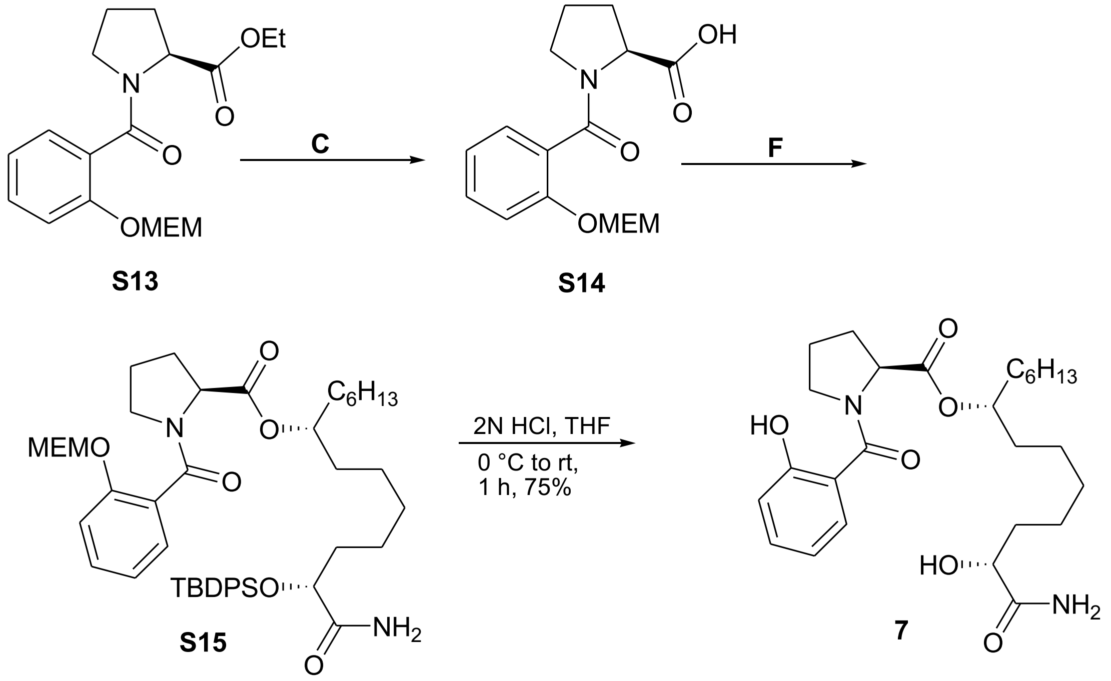

Using general procedure **C** ester **S13** (165 mg, 0.47 mmol) was hydrolyzed to (2-((2-methoxyethoxy)methoxy)benzoyl)-L-proline **S14** as a colourless gummy mass (140 mg, 92 %); R_f (10 % MeOH : CH_2Cl_2) = 0.3. $[\alpha]_D^{23}$ = -79.3 (*c* 1.7, $CHCl_3$). ^{1}H NMR (300 MHz, $CDCl_3$) mixture of rotamers (major) δ: 7.41-7.17 (3H, m); 7.06 (1H, dd, J = 7.8, 7.8 Hz); 5.29 (2H, s); 4.76 (1H, dd, J = 4.2, 8.2 Hz); 3.83-3.77 (2H, m); 3.57-3.52 (2H, m); 3.37 (3H, s); 3.40-3.32 (2H, m); 2.55-2.36 (1H, m); 2.26-1.80 (3H, m). ^{13}C NMR (75 MHz, $CDCl_3$) δ = major: 170.2, 168.5, 153.2, 131.4, 128.0, 126.6, 122.5, 115.5, 114.8, 94.1, 71.7, 68.2, 59.7, 59.2, 49.0, 28.6, 24.8. Anal. Calcd. for $C_{16}H_{21}NO_6$: C, 59.43; H, 6.55; N, 4.33. Found: C, 59.55; H, 6.54; N, 4.34.

Using general procedure **F**, the above acid was reacted with (2R,8R)-2-((tert-butyldiphenylsilyl)oxy)-8-hydroxytetradecanamide[1] **S4** (61 mg, 0.12 mmol) to give (7R,13R)-14-amino-13-((tert-butyldiphenylsilyl)oxy)-14-oxotetradecan-7-yl(2-((2-methoxyethoxy) methoxy)benzoyl)-L-prolinate **S15** as a yellow sticky solid (58 mg, 59 %); R_f (3 % MeOH : CH_2Cl_2) = 0.4. $[\alpha]_D^{23}$ = -25.1 (*c* 0.35, $CHCl_3$). ^{1}H NMR (300 MHz, $CDCl_3$) mixture of rotamers (major) δ: 7.69-6.98 (14H, m); 6.72 (1H, brs); 5.52 (1H, brs); 5.29 (2H, s); 4.70-4.58 (1H, m); 4.29-4.20 (1H, m); 3.87-3.72 (3H, m); 3.58-3.47 (2H, m); 3.35 (3H, s); 3.45-3.24 (2H, m); 2.40-1.77 (4H, m); 1.74-1.04 (29H, m); 0.87 (3H, t, J = 6.7 Hz). ^{13}C NMR (75 MHz, $CDCl_3$) δ: (major) 176.4, 172.1, 167.8, 153.3, 136.0 (× 2), 135.8 (× 4), 133.2, 132.8, 130.7, 130.4, 128.2, 128.1 (× 4), 122.4, 115.2, 94.1, 75.3, 74.4, 71.8, 68.1, 59.2, 48.4, 34.5, 34.2, 32.0, 31.9, 31.5, 30.0, 29.9, 29.5, 29.4, 29.3, 27.3, 25.4, 25.0, 23.0, 22.8, 22.78, 19.5, 14.3. Anal. Calcd. for $C_{46}H_{66}N_2O_8Si$: C, 68.79; H, 8.28; N, 3.49. Found: C, 68.67; H, 8.26; N, 3.48.

2N HCl (0.5 mL) was added dropwise to a stirred solution of the **S15** (50 mg, 0.06 mmol) in THF (0.5 mL) at 0 °C. The reaction mixture was stirred at room temperature for 1h. THF was removed *in vacuo*; the aqueous layer was extracted with ethyl acetate (2 × 7 mL). The combined organic extracts were washed with brine (5 mL), dried over anhydrous Na_2SO_4 and concentrated *in vacuo*. The product was purified using preparative TLC in 10% MeOH : CH_2Cl_2, to afford the title compound **7** as a pale yellow oil (25 mg, 75 %). R_f (10 % MeOH : CH_2Cl_2) = 0.4. $[\alpha]_D^{23}$ = -13.0 (*c* 1.0, $CHCl_3$). ^{1}H NMR (300 MHz, $CDCl_3$) δ: 10.66 (1H, s); 7.55-7.44 (1H, m); 7.40-7.30 (1H, m); 6.96 (1H, dd, *J* = 1.0, 8.2 Hz); 6.87 (1H, dd, *J* = 8.2, 8.2 Hz); 6.59 (1H, brs); 5.29 (1H, brs); 4.98 (1H, brs); 4.65 (1H, dd, *J* = 5.5, 8.6 Hz); 4.15-4.06 (1H, m); 3.95-3.74 (2H, m); 3.40-3.31 (1H, m); 2.43-2.21 (1H, m); 2.17-1.89 (3H, m); 1.83-1.07 (20H, m); 0.87 (3H, t, *J* = 6.7 Hz). ^{13}C NMR (75 MHz, $CDCl_3$) δ: 177.3, 172.4, 170.4, 159.1, 133.3, 128.2, 118.9, 117.95, 117.87, 75.5, 71.7, 60.7, 50.8, 34.6, 34.5, 34.4, 31.9, 29.4, 29.3, 28.6, 25.9, 25.6, 25.0, 24.7, 22.8, 14.3. Anal. Calcd. for $C_{26}H_{40}N_2O_6$: C, 65.52; H, 8.46; N, 5.88. Found: C, 65.63; H, 8.48; N, 5.89. HRMS**:** (ES+) calculated for $C_{26}H_{40}N_2O_6Na$ $(M + Na)^+$ 499.27786, Found: 499.27697.

1.3.7. Synthesis of compound 8. (7R,13R)-14-amino-13-hydroxy-14-oxotetradecan-7-yl (1S)-9-oxo-1,2,3,3a-tetrahydro-9H-benzo[e]pyrrolo[2,1-b][1,3]oxazine-1-carboxylate.

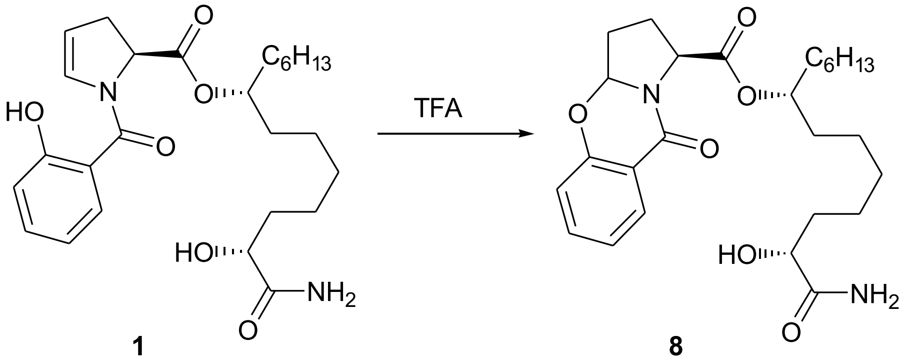

1 8

Compound **8** was synthesized as described in the literature.[2]

More polar diastereomer: R_f (50 % EtOAc: hexane) = 0.32. $[\alpha]_D^{23}$ = -54.0 (*c* 0.75, $CHCl_3$). ^1H NMR (600 MHz, $CDCl_3$) δ: 7.83 (1H, d, J = 7.6 Hz); 7.44 (1H, dd, J = 7.6, 7.6 Hz); 7.09 (1H, dd, J = 7.6, 7.6 Hz); 6.99 (1H, d, J = 7.6 Hz); 6.87 (1H, s); 5.56 (1H, dd, J = 6.2, 7.3 Hz); 5.48 (1H, s); 4.99-4.93 (1H, m); 4.60 (1H, d, J = 8.9 Hz); 4.29-4.26 (1H, m); 4.13-4.08 (1H, m); 2.50-2.44 (1H, m); 2.40-2.23 (2H, m); 2.20-2.15 (1H, m); 1.84-1.04 (20H, m); 0.85 (3H, t, J = 7.3 Hz);^{13}C NMR (150 MHz, $CDCl_3$) δ: 177.3, 170.6, 161.4, 157.8, 134.4, 127.8, 122.8, 118.9, 116.9, 88.5, 75.5, 71.0, 57.1, 34.4, 33.8, 33.7, 31.7, 30.2, 29.0, 27.7, 26.0, 25.3, 24.5, 24.0, 22.5, 14.0. Anal. Calcd. for $C_{26}H_{38}N_2O_6$ C, 65.80; H, 8.07; N, 5.90. Found: C, 65.87; H, 8.09; N, 5.91. HRMS**:** (ES+) calculated for $C_{26}H_{38}N_2O_6Na$ $(M + Na)^+$ 497.26221, Found: 497.26346.

1.3.8. Synthesis of compound 9. (2R,8R)-2,8-dihydroxytetradecanamide

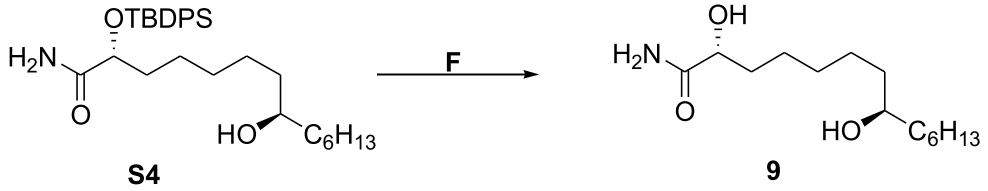

Using general procedure **F**, silyl ether **S4** (38 mg, 0.076 mmol) yielded the title compound **9** as an off white solid (12 mg, 63 %). R_f (2 % MeOH:CH_2Cl_2) = 0.3. mp. 100-102 ºC. $[\alpha]_D^{23}$ = +15.0 (*c* 0.5, $CHCl_3$). ^1H NMR (300 MHz, CH_3OH - *d_4*) δ: 3.97 (1H, dd, J = 3.9, 7.7 Hz); 3.50 (1H, brs); 1.83-1.67 (1H, m); 1.66-1.52 (1H, m); 1.50-1.22 (20H, m); 0.90 (3H, t, *J* = 7.0 Hz). ^{13}C NMR (75 MHz, CH_3OH - *d_4*) δ: 179.5, 71.5, 71.2, 37.3, 37.2, 34.4, 31.9, 29.4, 29.4, 25.6, 25.5, 24.9, 22.5, 13.2. Anal. Calcd. for $C_{14}H_{29}NO_3$: C, 64.83; H, 11.27; N, 5.40. Found: C, 64.75; H, 11.29; N, 5.41. HRMS**:** (ES+) calculated for $C_{14}H_{29}NO_3Na$ $(M + Na)^+$ 282.20396, Found: 282.20448.

1.3.9. Synthesis of compound 10. (2R, 8R)- 2,8 dihydroxytetradecanol.

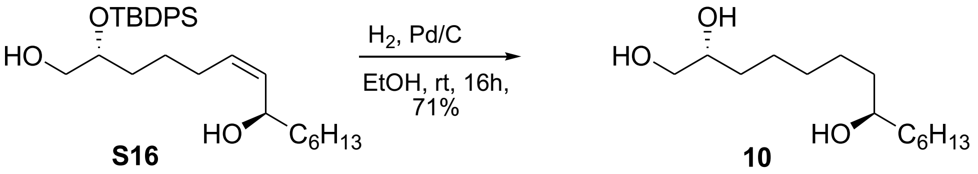

To a solution of diol **S16**[1] (100 mg, 0.274 mmol) in ethanol (7 mL) was added 10% Pd/C (10 mg). The suspension was evacuated under vacuum and flushed with H_2 gas (4 times). The reaction mixture was stirred under H_2 atmosphere at room temperature for 12 h. The reaction mixture was filtered through a plug of celite and the residue was washed with ethyl acetate (2 × 5 mL). The filtrate was concentrated *in vacuo*, the concentrate was triturated with diethyl ether (5 mL) to obtain alcohol **10** (50 mg, 74 %) as white solid. R_f (70 % ethyl acetate: hexane) = 0.2. mp 103-104 °C. $[\alpha]_D^{23}$ = +8.0 (*c* 0.5, MeOH). ^1H NMR (300 MHz, 300 MHz, CH_3OH - *d_4*) δ: 3.62-3.34 (4H, m); 1.60-1.22 (20H, m), 0.90 (3H, t, J = 6.9 Hz). ^{13}C NMR (75 MHz, CH_3OH - *d_4*) δ: 72.1, 71.2, 66.2, 37.3, 37.2, 33.2, 31.9, 29.7, 29.3, 25.6 (× 2), 25.5, 22.5, 13.2. Anal. Calcd. for $C_{14}H_{30}O_3$: C, 68.25; H, 12.27. Found: C, 68.33; H, 12.29. HRMS: (ES+) calculated for $C_{14}H_{30}O_3Na$ $(M + Na)^+$ 269.20872, Found: 269.20931.

1.3.10. Synthesis of compound 11. (R)-8-hydroxytetradecanamide

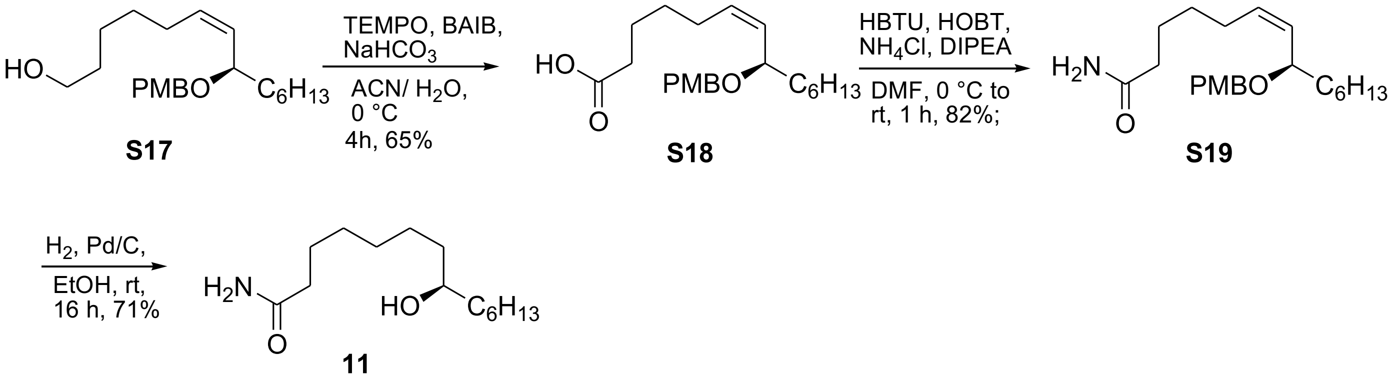

A suspension of alcohol **S17**[1] (700 mg, 2.02 mmol), $NaHCO_3$ (509 mg, 6.06 mmol) in CH_3CN : H_2O 1 : 1 (25 mL) was cooled to 0 °C and stirred for 10 min. TEMPO (63 mg, 0.40 mmol) and bis(acetoxy)iodobenzene (1.62 gm, 5.05 mmol), were added sequentially in one portion and the solution was stirred at 0 °C for 4 h in the dark. Saturated aq. $NaHCO_3$ (20 mL) was added at 0 °C and the aqueous layer was extracted with ethyl acetate (2 × 25 mL). The combined organic extracts were washed with brine (20 mL), dried over anhydrous Na_2SO_4 and concentrated *in vacuo*. The crude oil was purified using flash column chromatography with 0-30% ethyl acetate: petroleum ether) to furnish carboxylic acid **S18** (475 mg, 65 %) as white translucent oil. R_f (30 % ethyl acetate: hexane) = 0.25. $[\alpha]_D^{23}$ = +28.5 (*c* 1.00, $CHCl_3$). ^{1}H NMR (300 MHz, $CDCl_3$) δ: 7.24 (2H, d, *J* = 9.1 Hz); 6.86 (2H, d, *J* = 9.1 Hz); 5.58 (1H, dt, *J* = 11.2, 7.4 Hz); 5.32 (1H, dd, *J* = 11.2, 9.5 Hz); 4.50 (1H, d, *J* = 12.3 Hz); 4.26 (1H, d, *J* = 12.3 Hz); 4.11-4.02 (1H, m); 3.80 (3H, s); 2.35 (2H, t, *J* = 7.3 Hz); 2.16-1.93 (2H, m); 1.74-1.18 (14H, m); 0.87 (3H, t, *J* = 7.3 Hz). ^{13}C NMR (75 MHz, $CDCl_3$) δ: 179.7, 159.2, 132.6, 131.9, 131.3, 129.5 (× 2), 113.9 (× 2), 74.0, 69.6, 55.5, 35.9, 34.1, 32.1, 29.5, 29.4, 27.7, 25.6, 24.6, 22.8, 14.3. Anal. Calcd. for $C_{22}H_{34}O_4$: C, 72.89; H, 9.45. Found: C, 72.77; H, 9.44.

A solution of carboxylic acid **S18** (475 mg, 1.31 mmol), NH_4Cl (282.3 mg, 5.27 mmol) in dry DMF (18 mL, 0.07 M) was cooled to 0 °C. HOBT (534 mg, 3.93 mmol) and HBTU (1.5 gm, 3.93 mmol) were added, followed by DIPEA (1.87 mL, 5.27 mmol). The reaction mixture was warmed to room temperature and stirred for 1 h. Ice pieces were added, and then the aqueous layer was extracted with ethyl acetate (2 × 20 mL). The combined organic extracts were washed with cold brine (3 × 15 mL) and dried over anhydrous Na_2SO_4. After removal of the solvent *in vacuo*, the residue was purified using flash column chromatography with 0-50% ethyl acetate: petroleum ether to furnish **S19** (389 mg, 82 %) as a colourless oil. R_f (50 % ethyl acetate: hexane) = 0.33. $[\alpha]_D^{23}$ = +17.4 (*c* 0.5, $CHCl_3$). ^{1}H NMR (300 MHz, $CDCl_3$) δ: 7.24 (2H, d, *J* = 8.9 Hz); 6.86 (2H, d, *J* = 8.9 Hz); 5.58 (1H, dt, *J* = 11.2, 7.4 Hz); 5.45 (2H, brs); 5.31 (1H, dd, *J* = 11.2, 9.3 Hz); 4.49 (1H, d, *J* = 11.7 Hz); 4.26 (1H, d, *J* = 11.7 Hz); 4.12-4.00 (1H, m); 3.80 (3H, s); 2.20 (2H, t, *J* = 7.6 Hz); 2.15-1.93 (2H, m); 1.74-1.17 (14H, m); 0.87 (3H, t, *J* = 7.7 Hz). ^{13}C NMR (75 MHz, $CDCl_3$) δ = 175.4, 159.2, 132.7, 131.8, 131.3, 129.5 (× 2), 113.9 (× 2),

74.0, 69.6, 55.5, 35.9, 35.8, 32.0, 29.5 (× 2), 27.7, 25.6, 25.4, 22.8, 14.3. Anal. Calcd. for $C_{22}H_{35}NO_3$: C, 73.09; H, 9.76; N, 3.87. Found: C, 73.17; H, 9.73; N, 3.87.

To a solution of **S19** (389 mg, 1.083 mmol) in ethanol (30 mL) was added 10% Pd/C (200 mg). The suspension was evacuated under vacuum and flushed with H_2 gas (× 4). The reaction mixture was stirred under H_2 at room temperature for 12 h, then it was filtered through a plug of celite and the residue was washed with ethyl acetate (2 × 20 mL). The filtrate was concentrated *in vacuo;* the concentrate was triturated with diethyl ether (10 mL) to obtain **(**R)-8-hydroxytetradecanamide **11** (150 mg, 71 %) as white solid. R_f (50 % ethyl acetate: hexane) = 0.15. mp :97-99 ºC.$[\alpha]_D^{23}$ = +9.0 (*c* 1.0, $CHCl_3$). ^{1}H NMR (300 MHz, CD_3OD) δ = 3.49 (1H, brs), 2.49-2.40 (1H, m); 2.22-2.15 (3H, s); 1.66-1.22 (20H, m); 0.90 (3h, t, J = 6.7 Hz); ^{13}C NMR (75 MHz, CD_3OD) δ = 178.1, 71.2, 37.3, 35.3, 31.9, 29.6, 29.5, 29.4, 29.3, 29.1, 25.7, 25.6, 22.5, 13.3. Anal. Calcd. for $C_{14}H_{29}NO_2$: C, 69.09; H, 12.01; N, 5.75. Found: C, 69.15; H, 12.03; N, 5.74. HRMS**:** (ES+) calculated for $C_{14}H_{29}NO_2Na$ $(M + Na)^+$ 266.20905, Found: 266.20960.

1.4. References

1) R. D. Kaduskar, A. A. Dhavan, S. Dallavalle, L. Scaglioni, L. Musso Total synthesis of the salicyldehydroproline-containing antibiotic *Tetrahedron*, **2016**, *72*, 2034−2041.

2) A. D. Steele, C. E. Keohane, K. W. Knouse, S. E. Rossiter, S. J. Williams, W. M. Wuest Diverted total synthesis of promysalin analogs demonstrates that an iron-binding motif is responsible for its narrow-spectrum antibacterial activity *J. Am. Chem. Soc.* **2016**, *138*, 5833−5836.

1.4 Spectral data

Compound S1

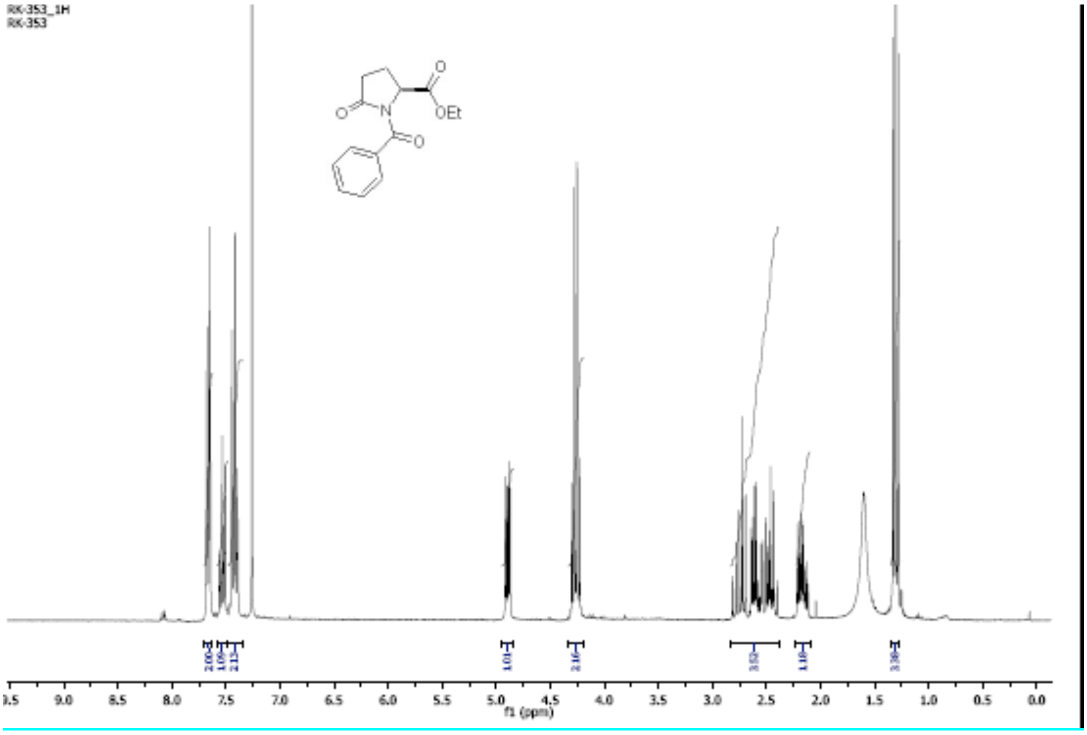
RK-353_1H
RK-353
O
OEt
N
O
O
f1 (ppm)

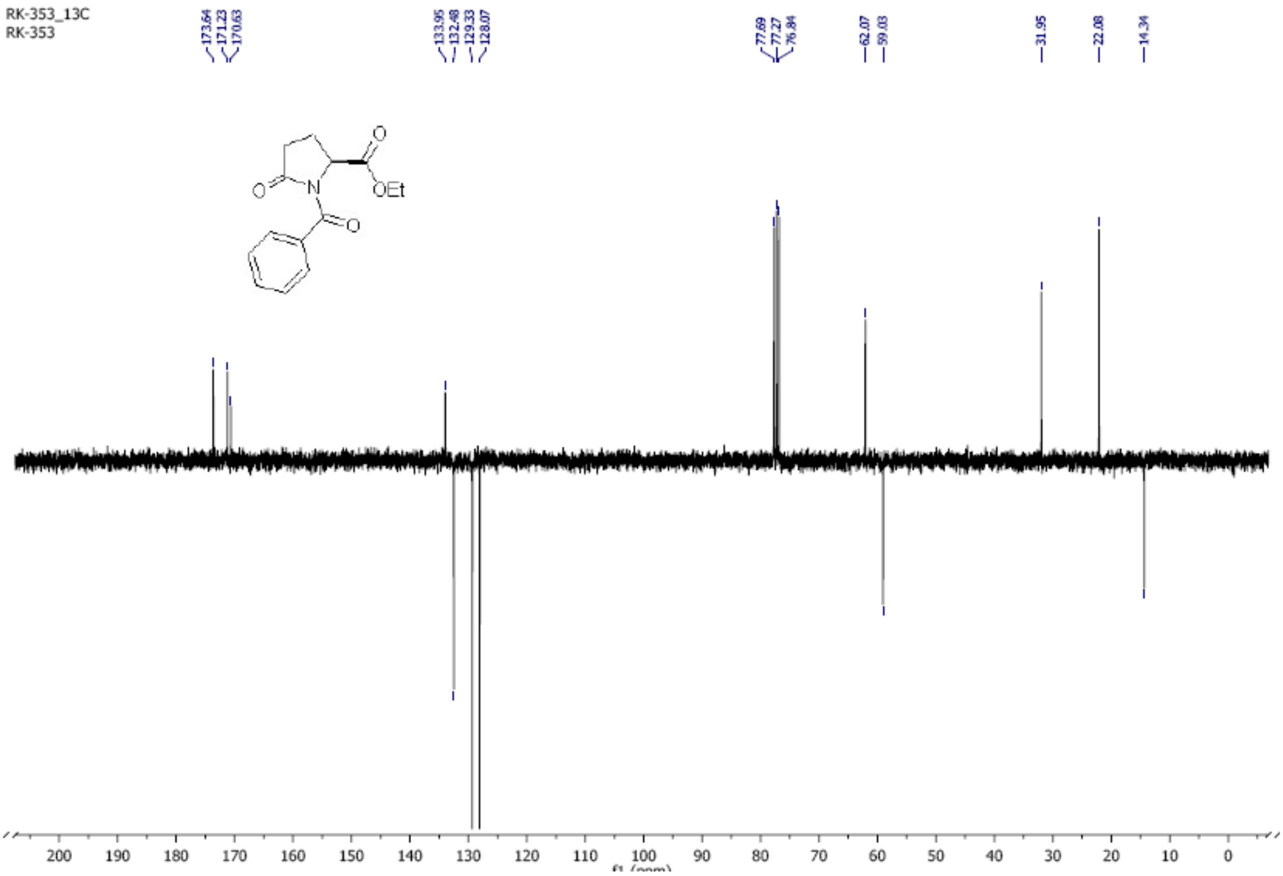
RK-353_13C
RK-353
173.64
171.23
170.63
133.95
132.48
129.33
128.07
77.69
77.27
76.84
62.07
59.03
31.95
22.08
14.34
O
OEt
N
O
O

Compound S2

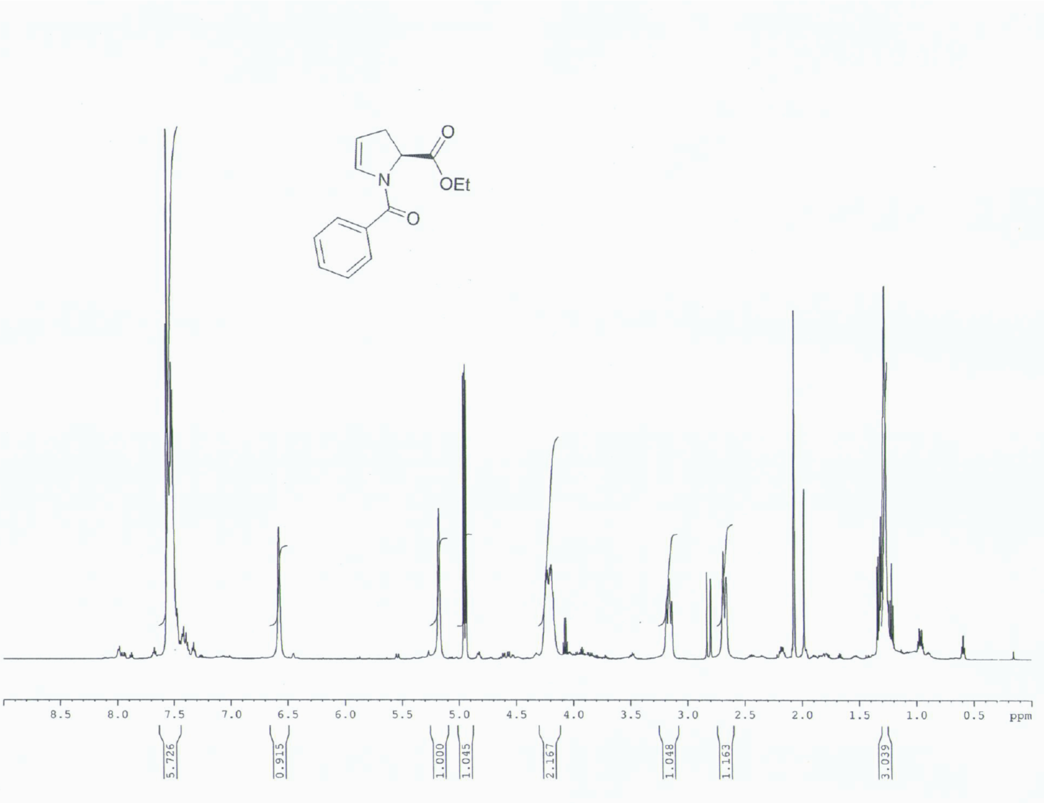
O
N
OEt
O
8.5 8.0 7.5 7.0 6.5 6.0 5.5 5.0 4.5 4.0 3.5 3.0 2.5 2.0 1.5 1.0 0.5 ppm
5.726
0.915
1.000
1.045
2.167
1.048
1.163
3.039

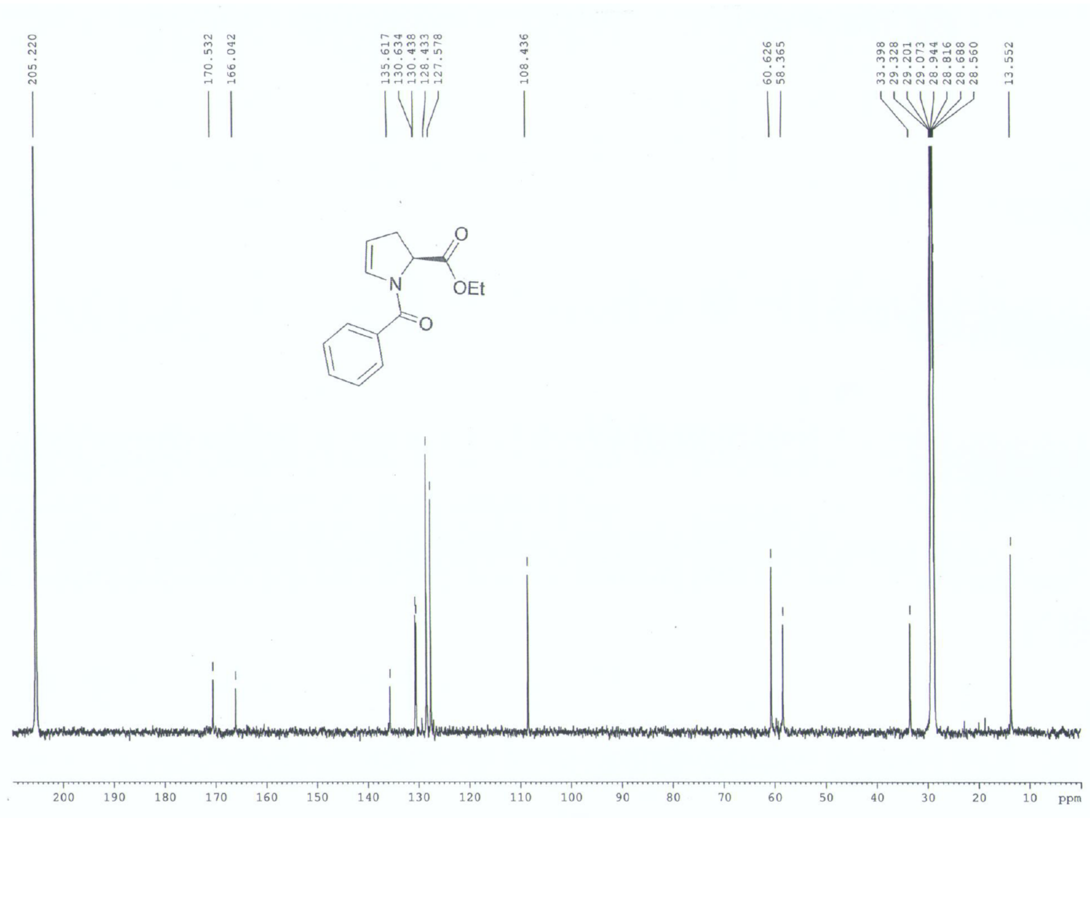
205.220
170.532
166.042
135.617
130.634
130.438
128.433
127.578
108.436
60.626
58.365
33.398
29.328
29.201
29.073
28.944
28.816
28.688
28.560
13.552
O
N
OEt
O
200 190 180 170 160 150 140 130 120 110 100 90 80 70 60 50 40 30 20 10 ppm

Compound S3

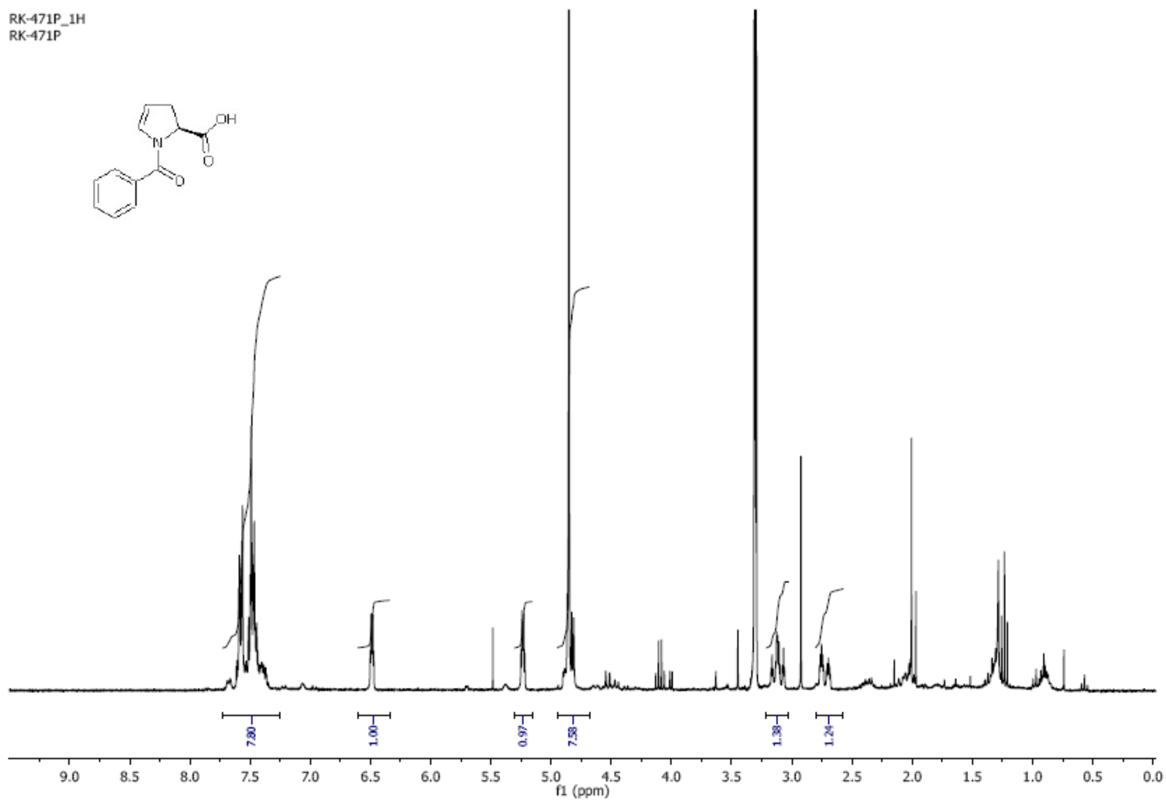
RK-471P_1H
RK-471P
OH
N
O
O
7.80
1.00
0.97
7.58
1.38
1.24
9.0 8.5 8.0 7.5 7.0 6.5 6.0 5.5 5.0 4.5 4.0 3.5 3.0 2.5 2.0 1.5 1.0 0.5 0.0
f1 (ppm)

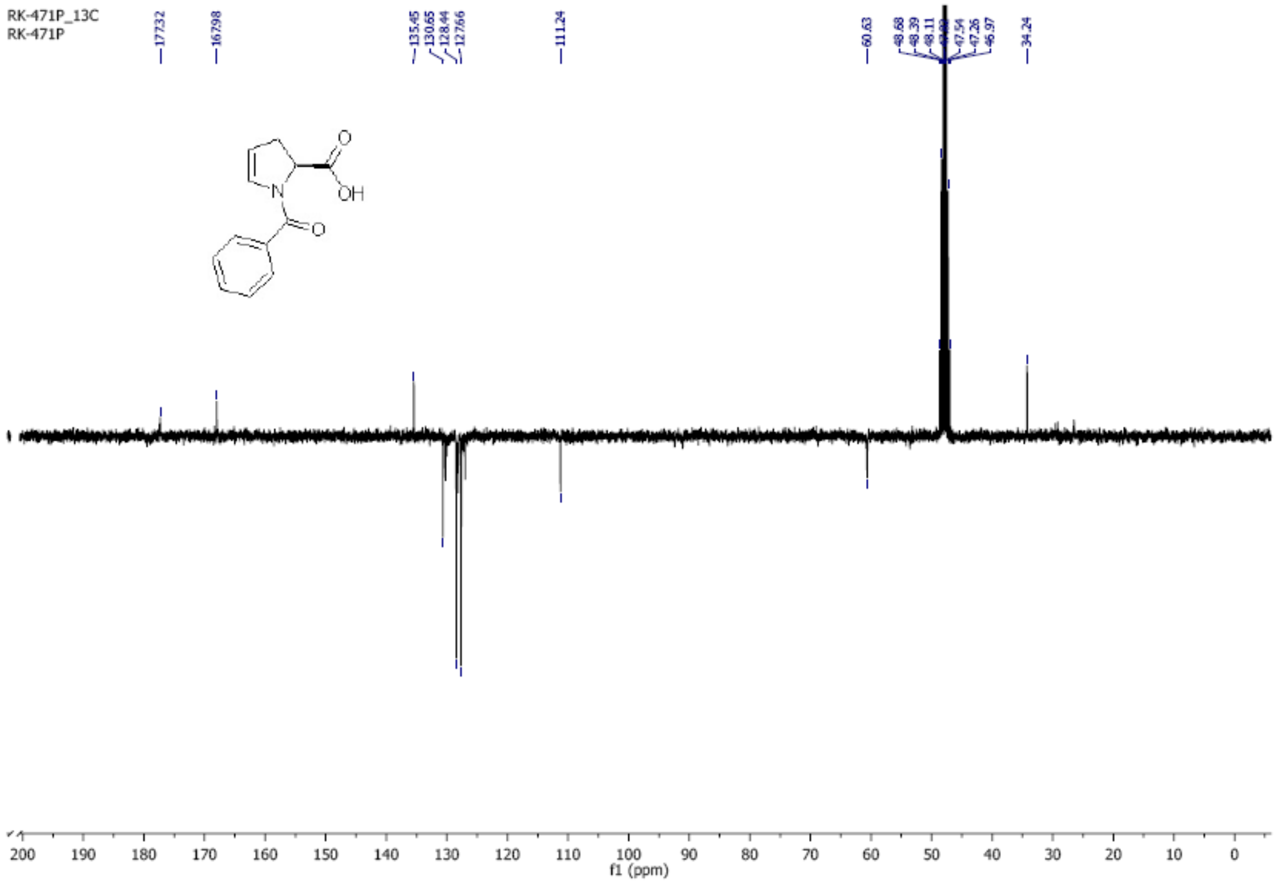
RK-471P_13C
RK-471P
177.32
167.98
135.45
130.65
128.44
127.66
111.24
60.63
48.68
48.39
48.11
47.54
47.26
46.97
34.24
O
OH
N
O
200 190 180 170 160 150 140 130 120 110 100 90 80 70 60 50 40 30 20 10 0
f1 (ppm)

Compound 2

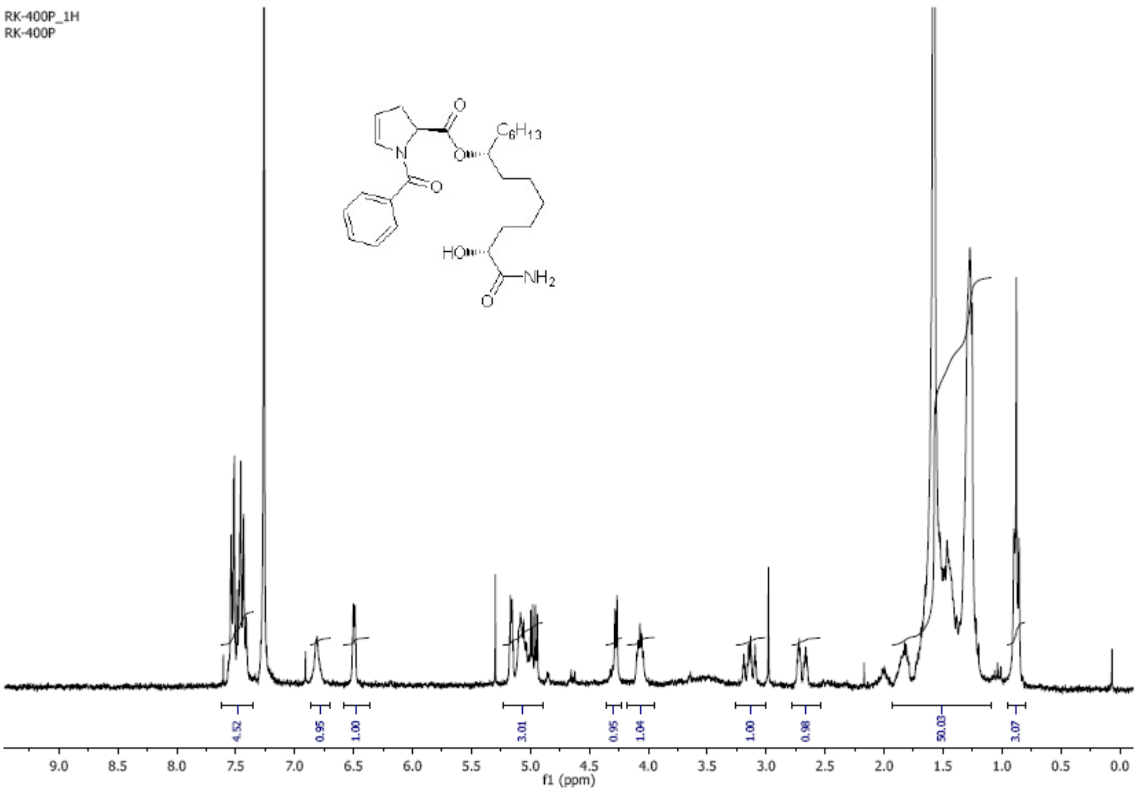

RK-400P_1H
RK-400P
C6H13
HO
NH2
4.52
0.95
1.00
3.01
0.95
1.04
1.00
0.98
50.03
3.07
f1 (ppm)

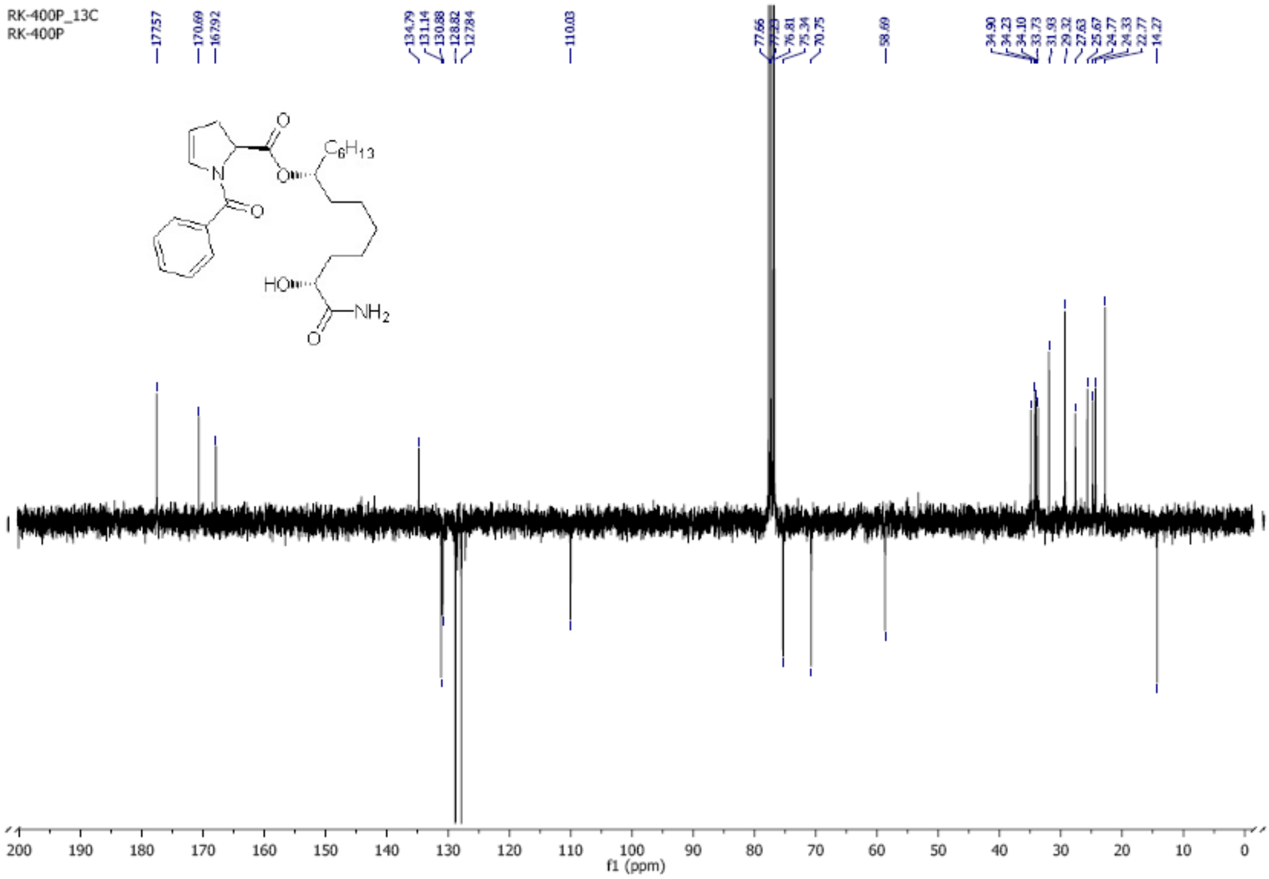

RK-400P_13C
RK-400P
177.57
170.69
167.92
134.79
131.14
130.88
128.82
127.84
110.03
77.66
76.81
75.34
70.75
58.69
34.90
34.23
34.10
33.73
31.93
29.32
27.63
25.67
24.77
24.33
22.77
14.27
C6H13
HO
NH2
f1 (ppm)

Compound S6

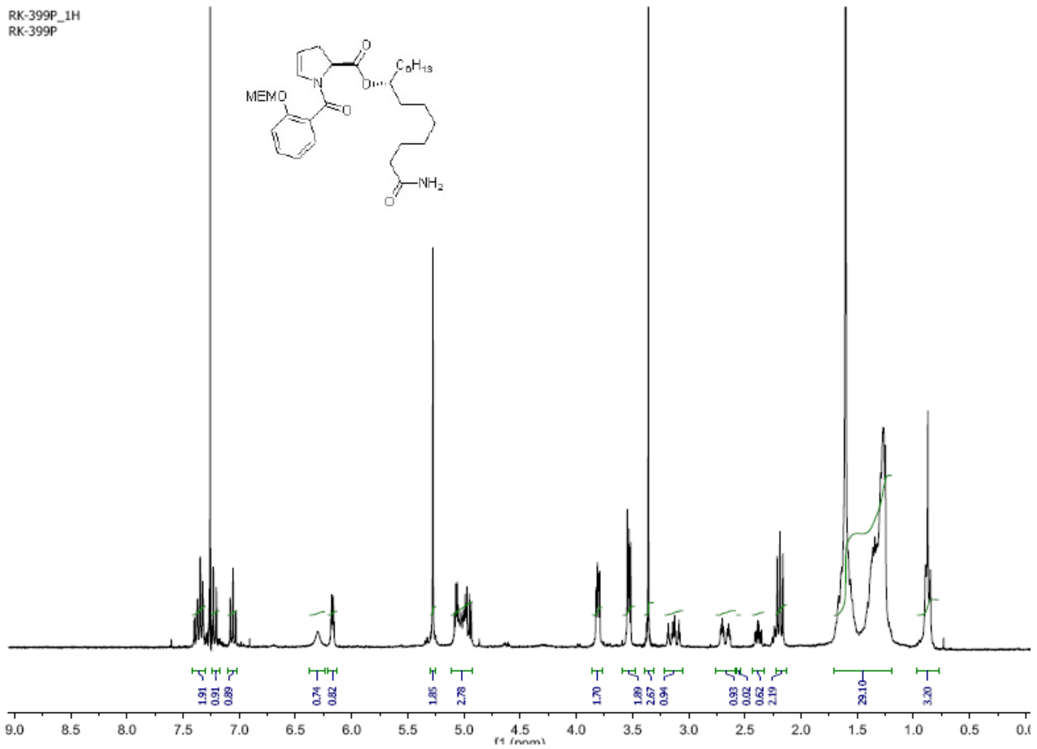

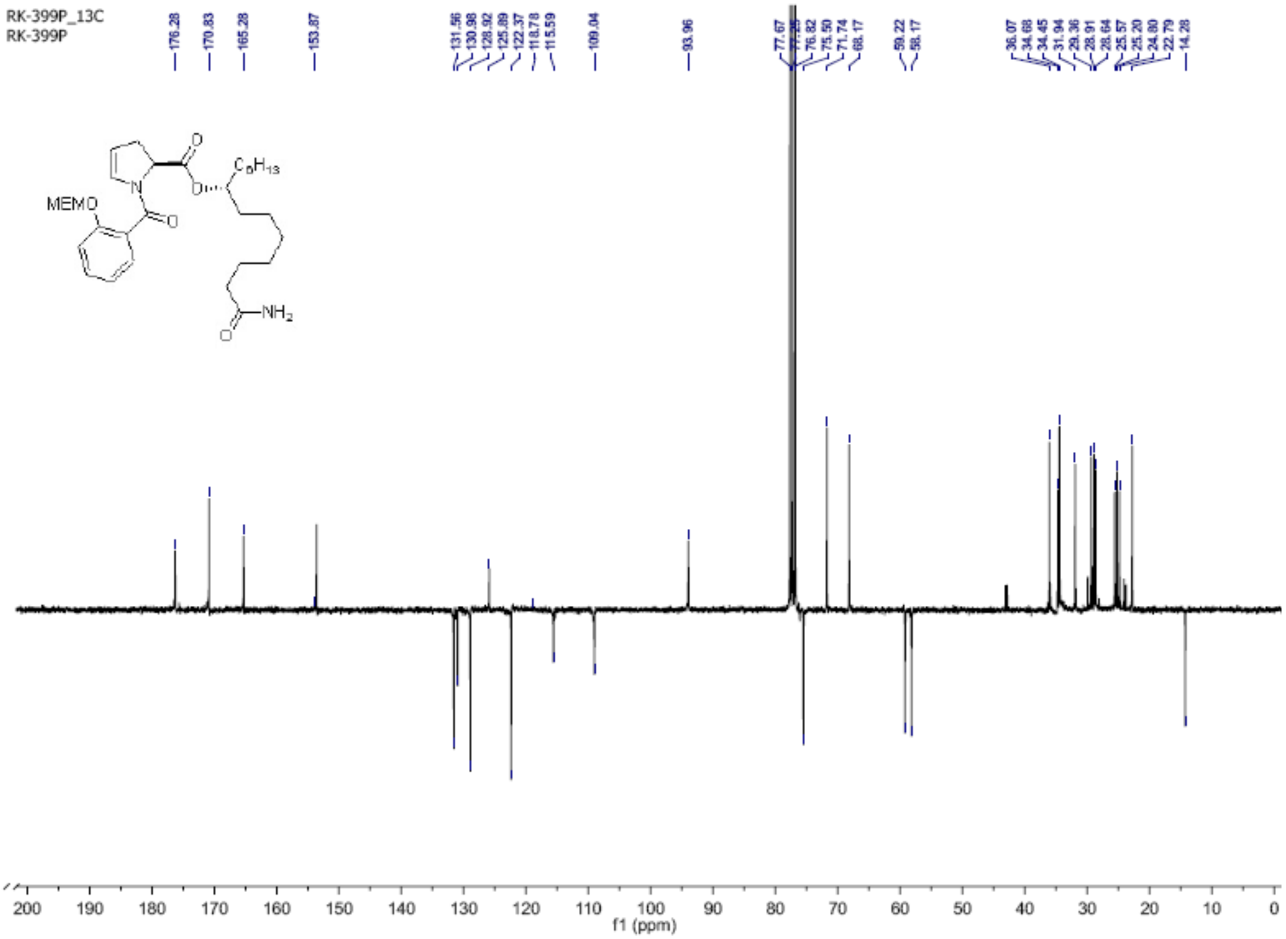

Compound 3

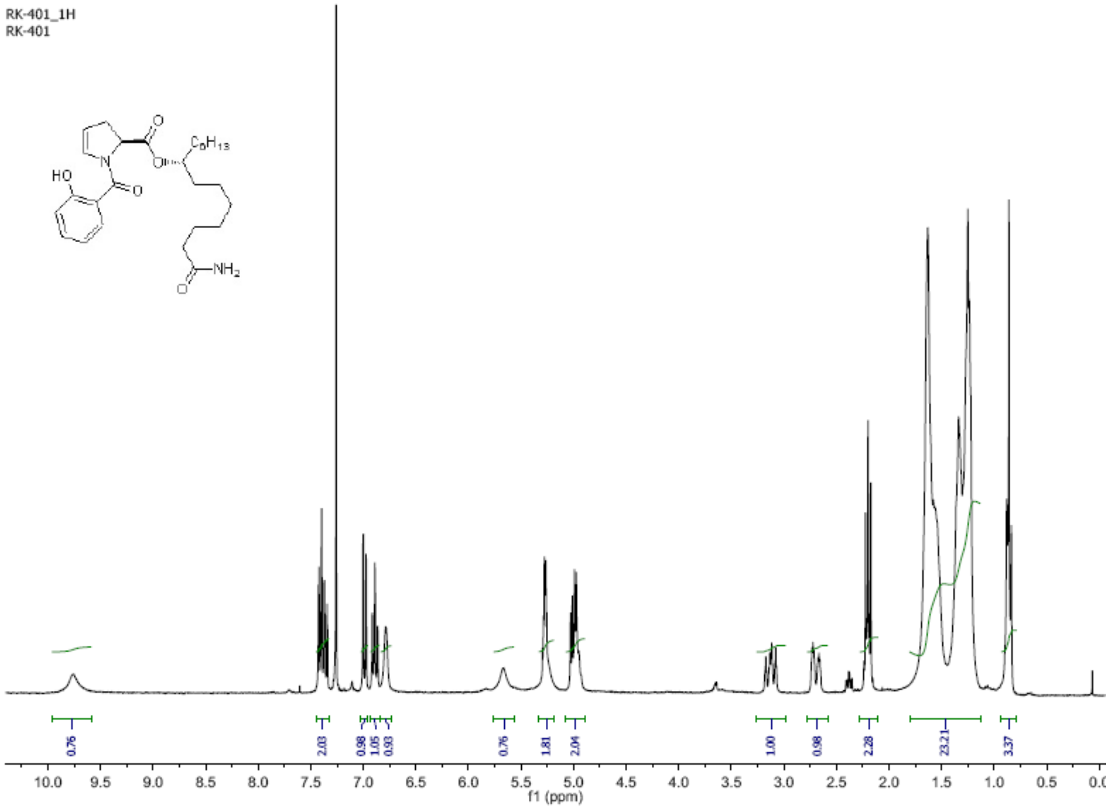

RK-401_1H
RK-401
HO
O
N
O
C6H13
NH2
0.76
2.03
0.98
1.05
0.93
0.76
1.81
2.04
1.00
0.98
2.28
23.21
3.37
f1 (ppm)

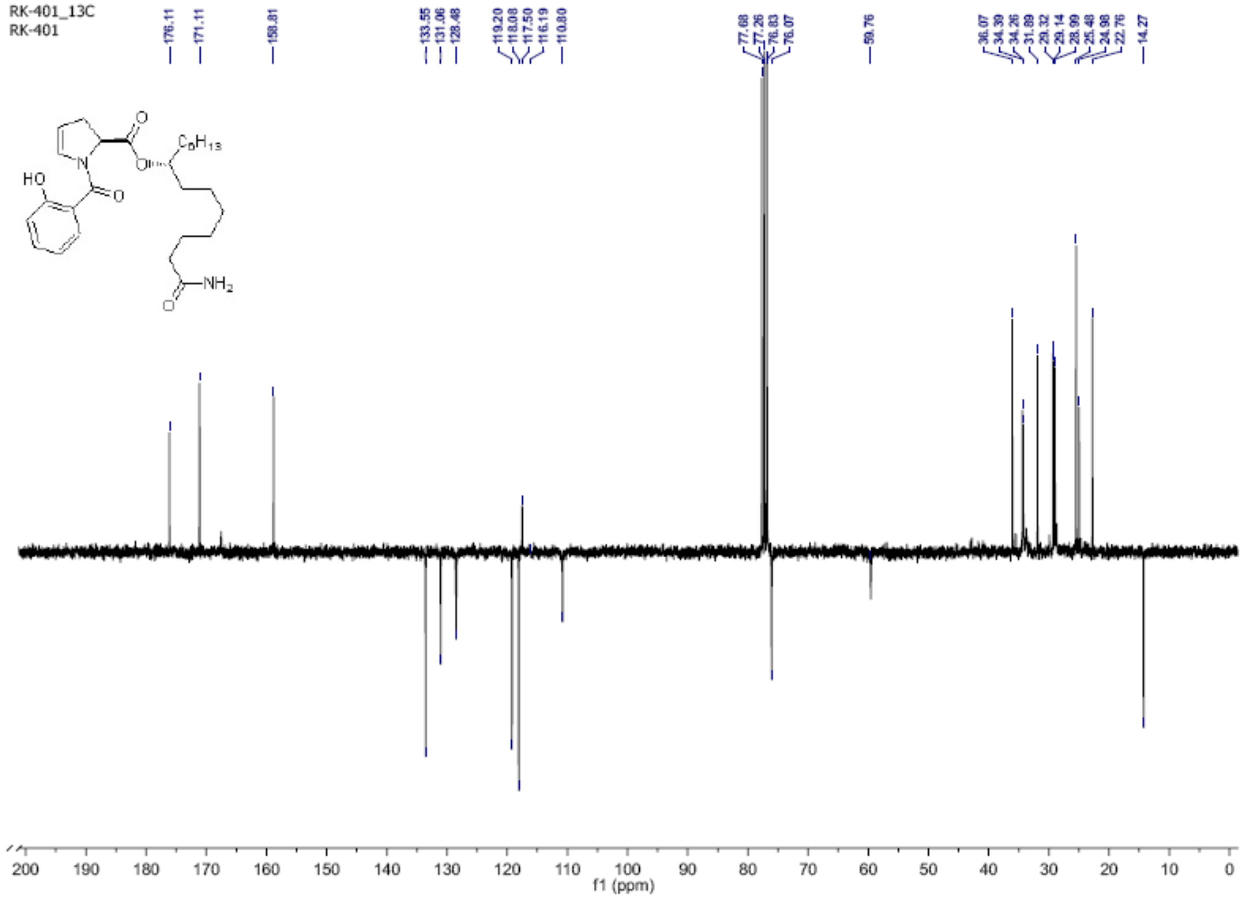

RK-401_13C
RK-401
176.11
171.11
158.81
133.55
131.06
128.48
119.20
118.08
117.50
116.19
110.80
77.68
77.26
76.83
76.07
59.76
36.07
34.39
34.26
31.89
29.32
29.14
28.99
25.48
24.98
22.76
14.27
HO
N
C6H13
NH2
f1 (ppm)

Compound S7

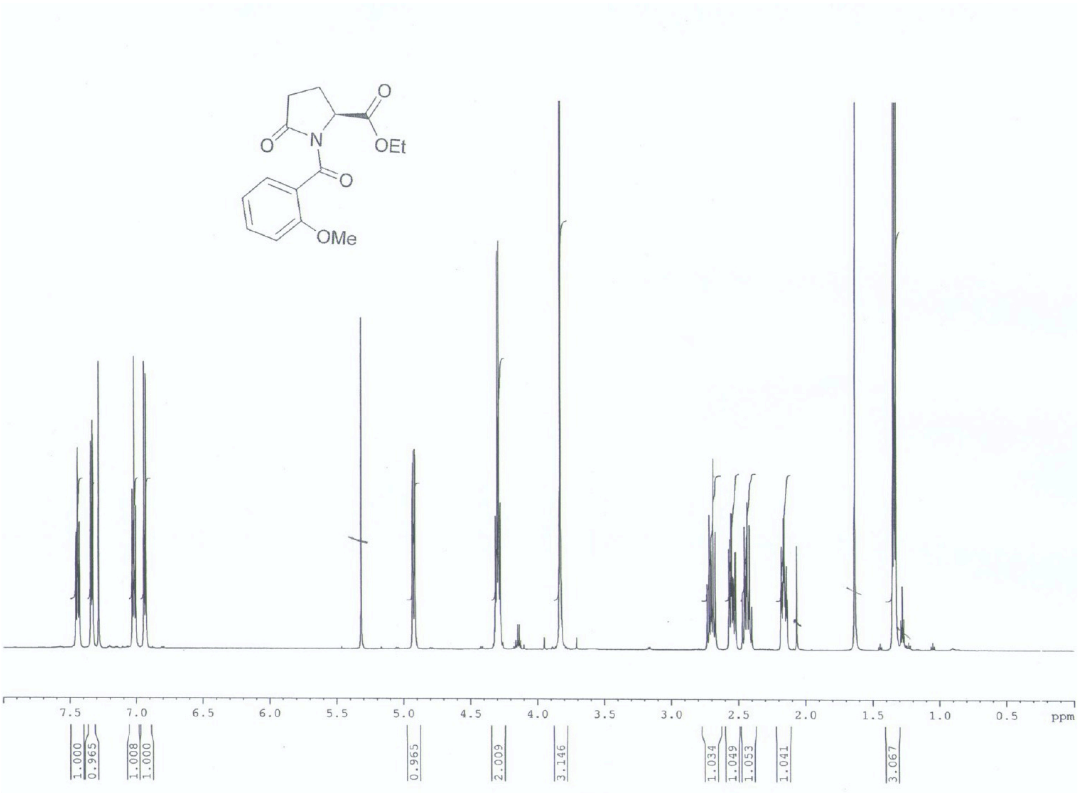

O
N
OEt
O
O
OMe
7.5
7.0
6.5
6.0
5.5
5.0
4.5
4.0
3.5
3.0
2.5
2.0
1.5
1.0
0.5
ppm
1.000
0.965
1.008
1.000
0.965
2.009
3.146
1.034
1.049
1.053
1.041
3.067

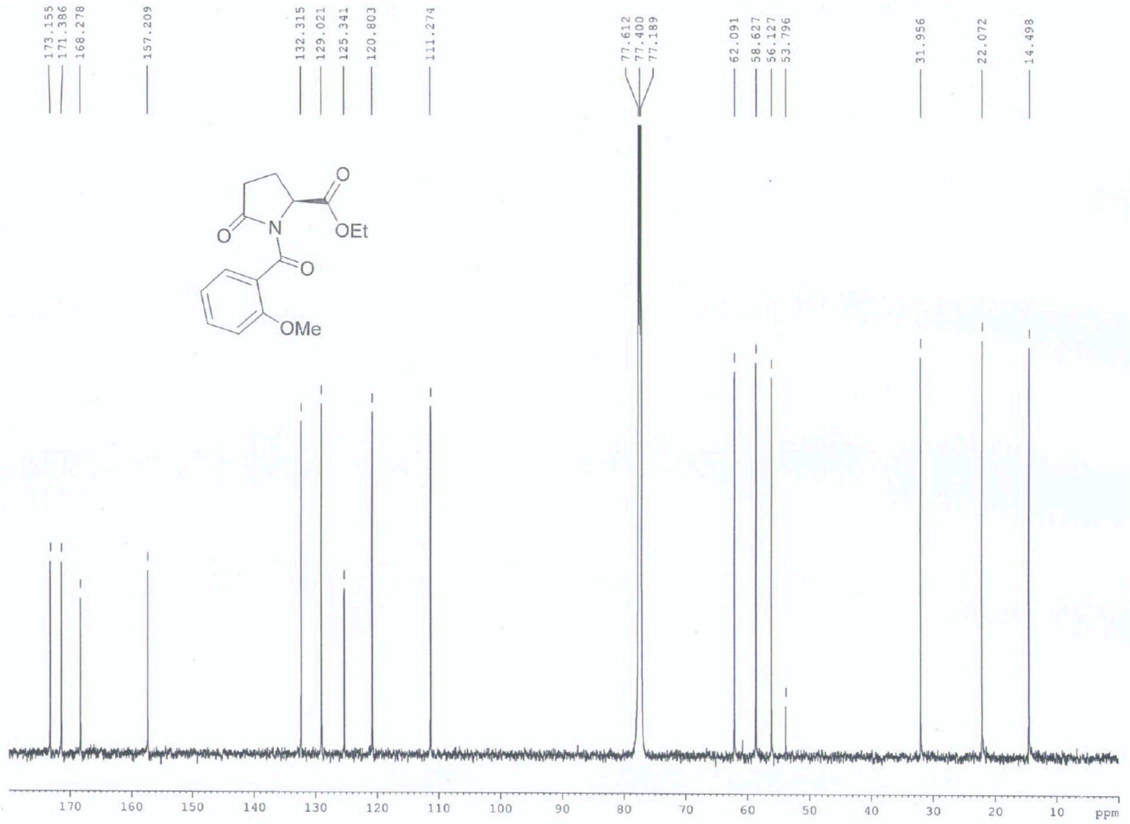

173.155
171.386
168.278
157.209
132.315
129.021
125.341
120.803
111.274
77.612
77.400
77.189
62.091
58.627
56.127
53.796
31.956
22.072
14.498
O
N
OEt
O
O
OMe
170
160
150
140
130
120
110
100
90
80
70
60
50
40
30
20
10
ppm

Compound S8

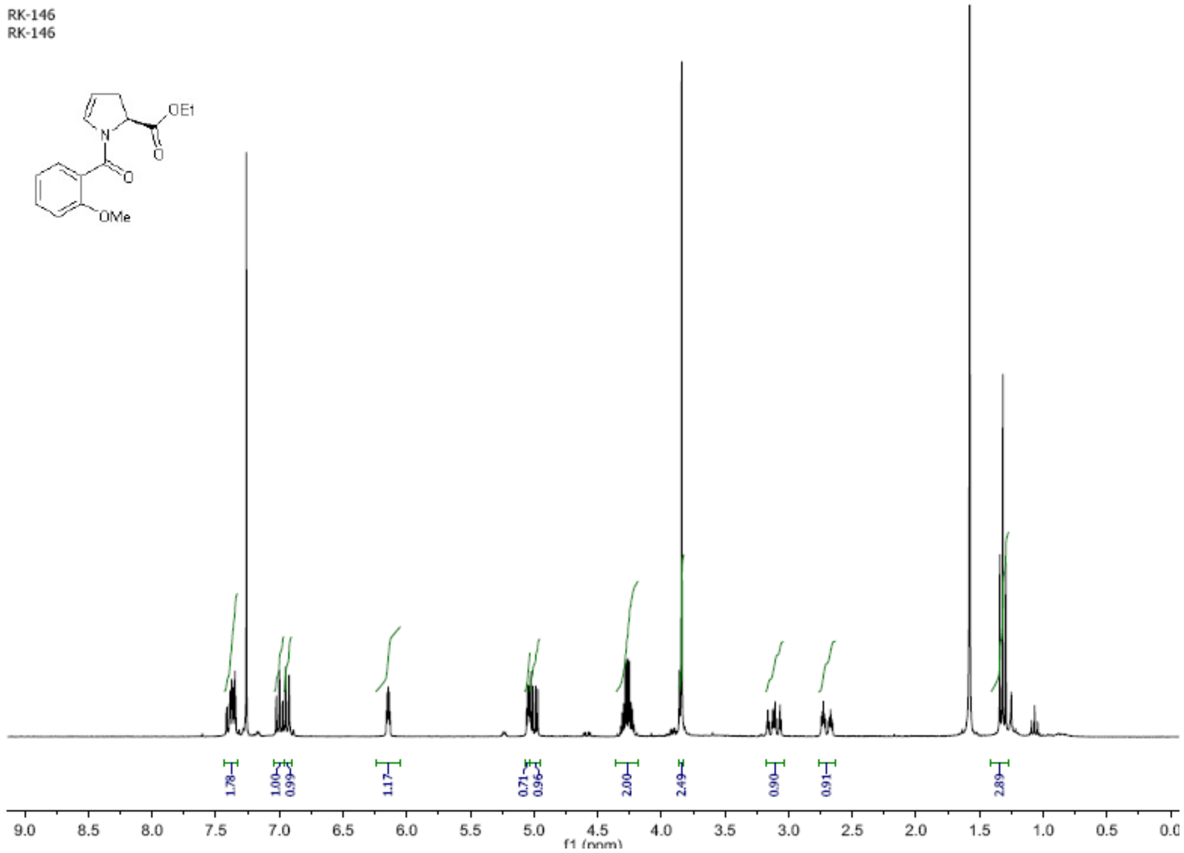
RK-146
RK-146
OEt
O
N
O
OMe
1.78
1.00
0.99
1.17
0.71
0.96
2.00
2.49
0.90
0.91
2.89
9.0
8.5
8.0
7.5
7.0
6.5
6.0
5.5
5.0
4.5
4.0
3.5
3.0
2.5
2.0
1.5
1.0
0.5
0.0
f1 (ppm)

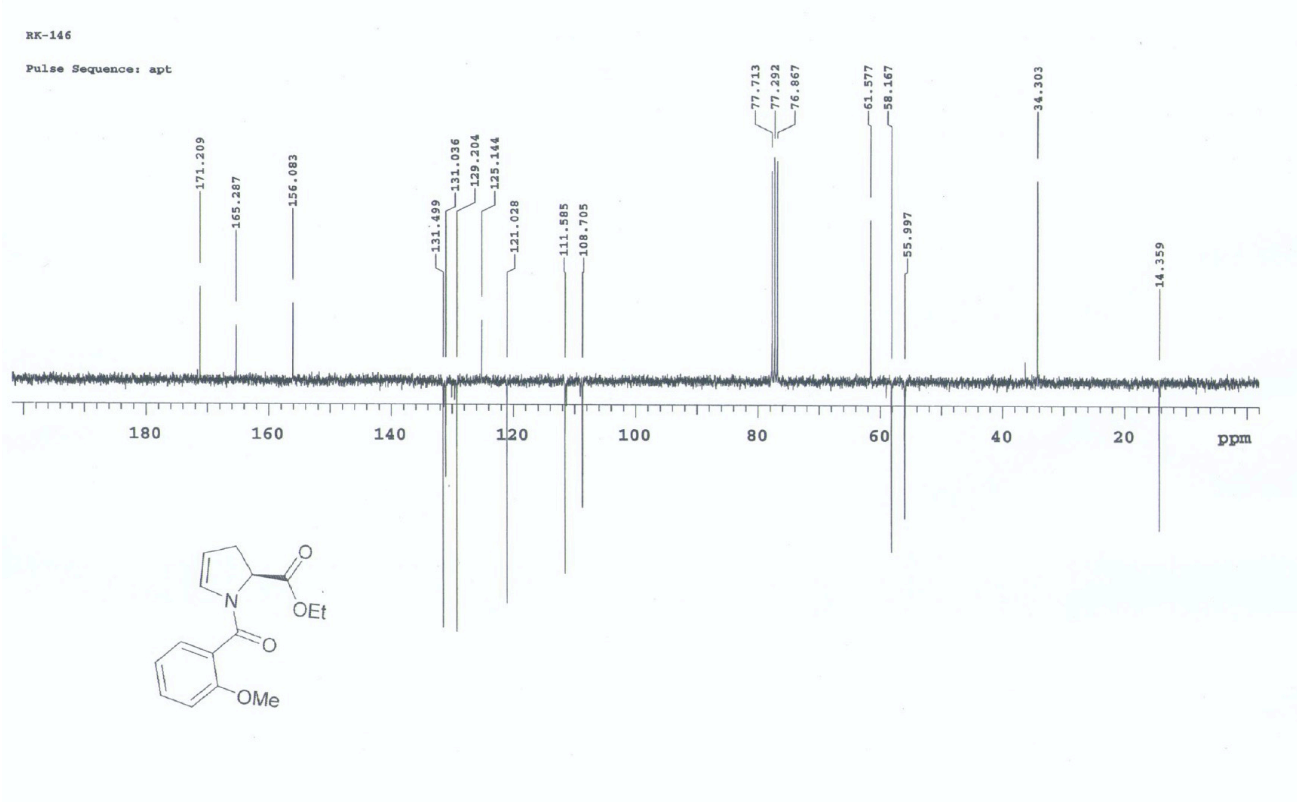
RK-146
Pulse Sequence: apt
171.209
165.287
156.083
131.499
131.036
129.204
125.144
121.028
111.585
108.705
77.713
77.292
76.867
61.577
58.167
55.997
34.303
14.359
180
160
140
120
100
80
60
40
20
ppm
O
N
OEt
O
OMe

Compound S9

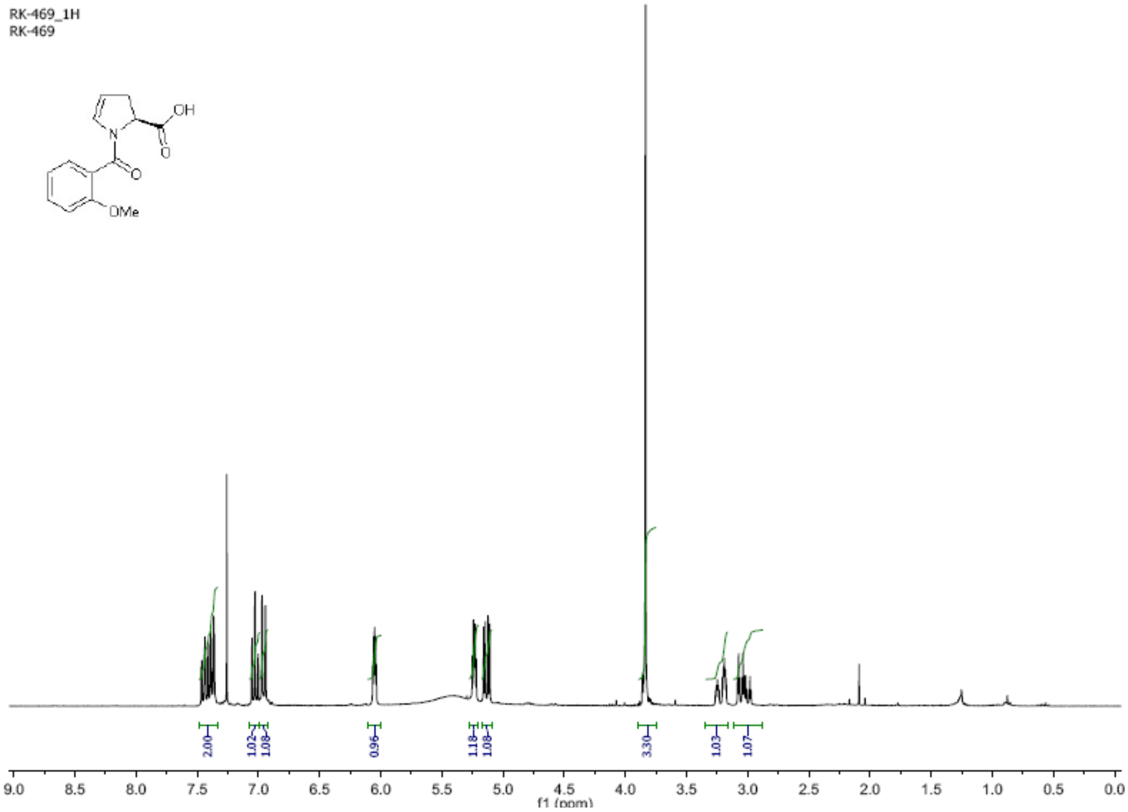
RK-469_1H
RK-469
OH
N
O
OMe
2.00
1.02
1.08
0.96
1.18
1.08
3.30
1.03
1.07
9.0 8.5 8.0 7.5 7.0 6.5 6.0 5.5 5.0 4.5 4.0 3.5 3.0 2.5 2.0 1.5 1.0 0.5 0.0
f1 (ppm)

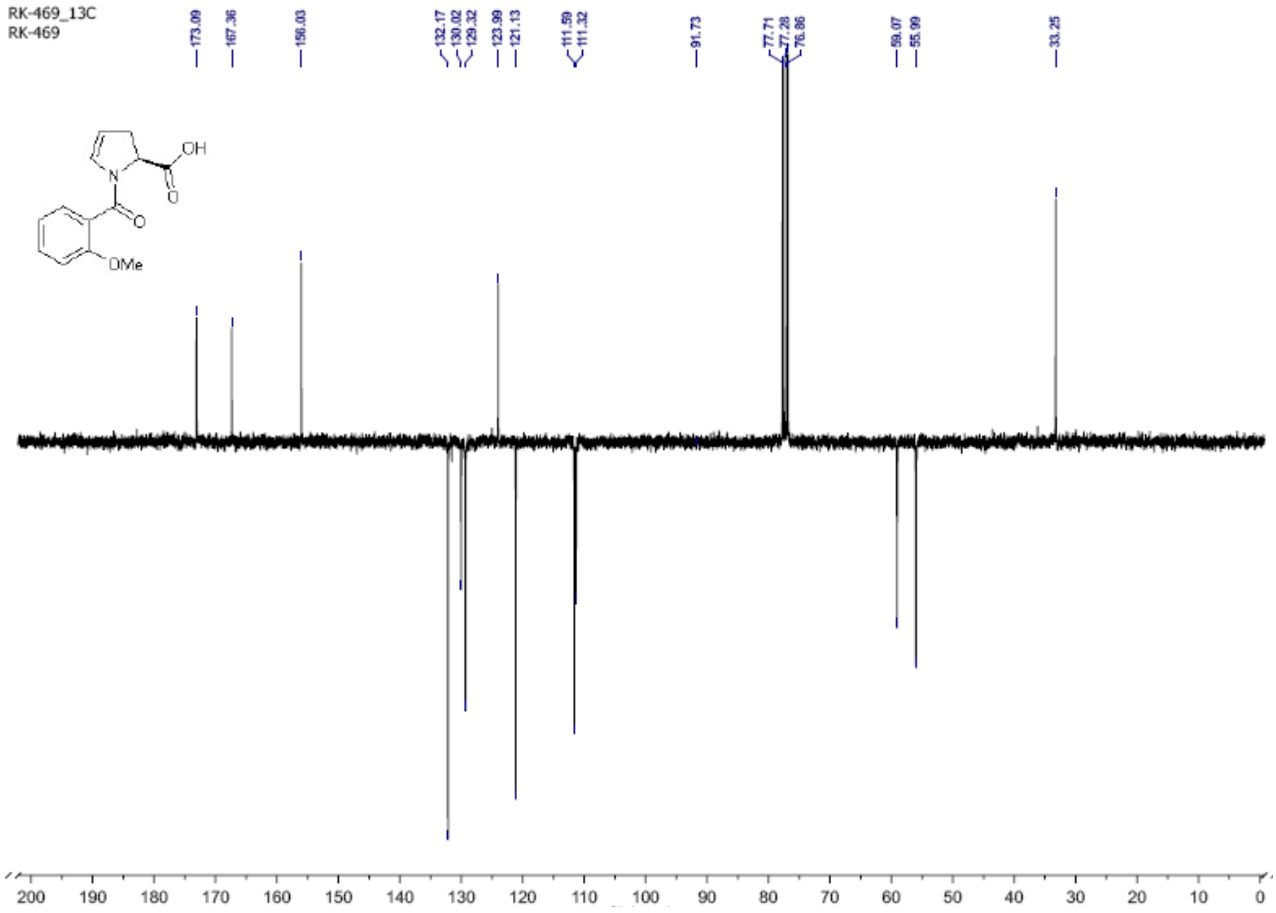
RK-469_13C
RK-469
173.09
167.36
156.03
132.17
130.02
129.32
123.99
121.13
111.59
111.32
91.73
77.71
77.28
76.86
59.07
55.99
33.25
OH
N
O
OMe
200 190 180 170 160 150 140 130 120 110 100 90 80 70 60 50 40 30 20 10 0

Compound S10

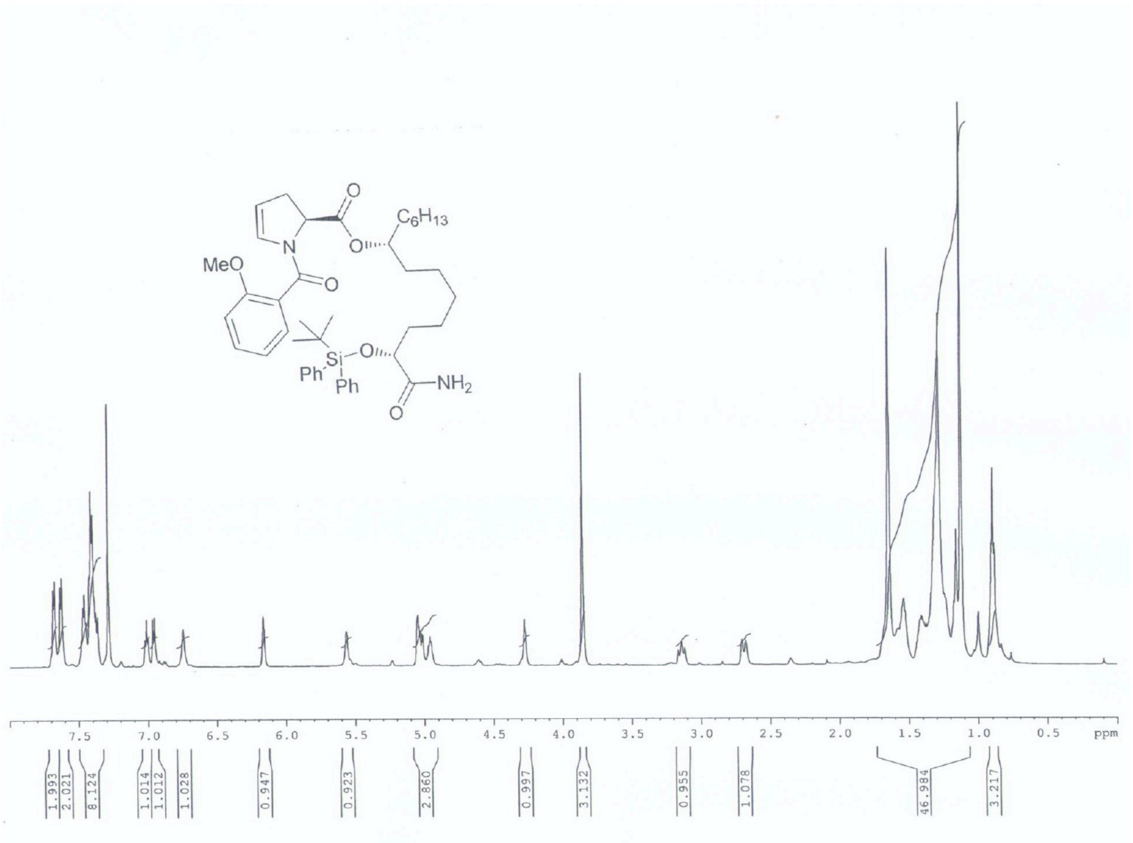

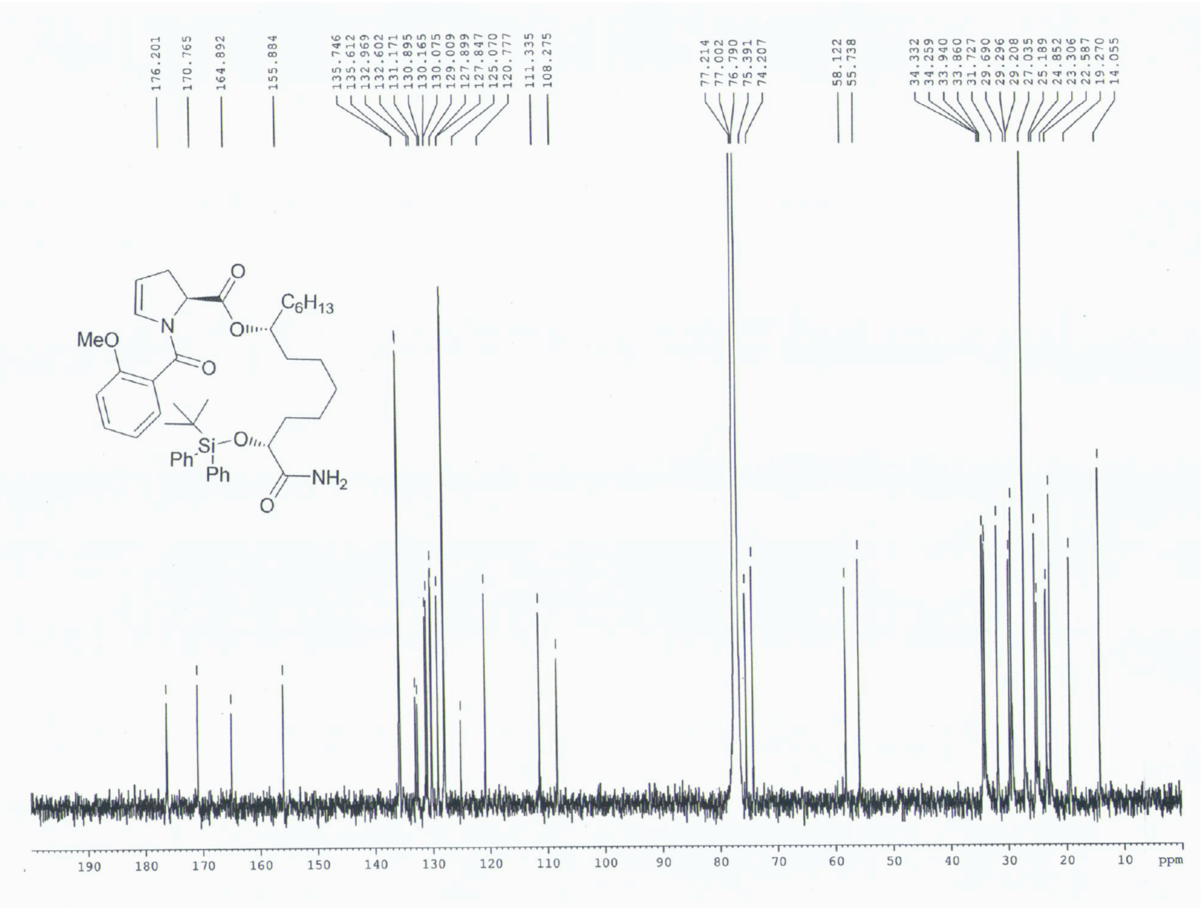

Compound 4

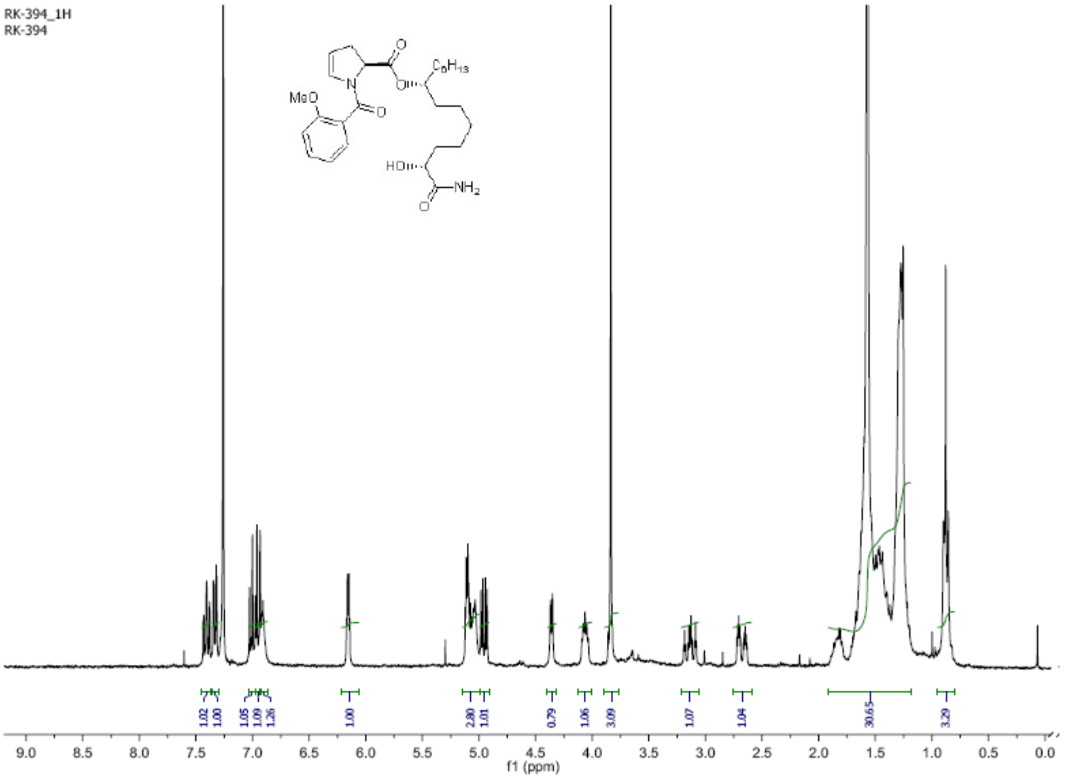

RK-394_1H
RK-394
MeO
C6H13
HO
NH2
f1 (ppm)

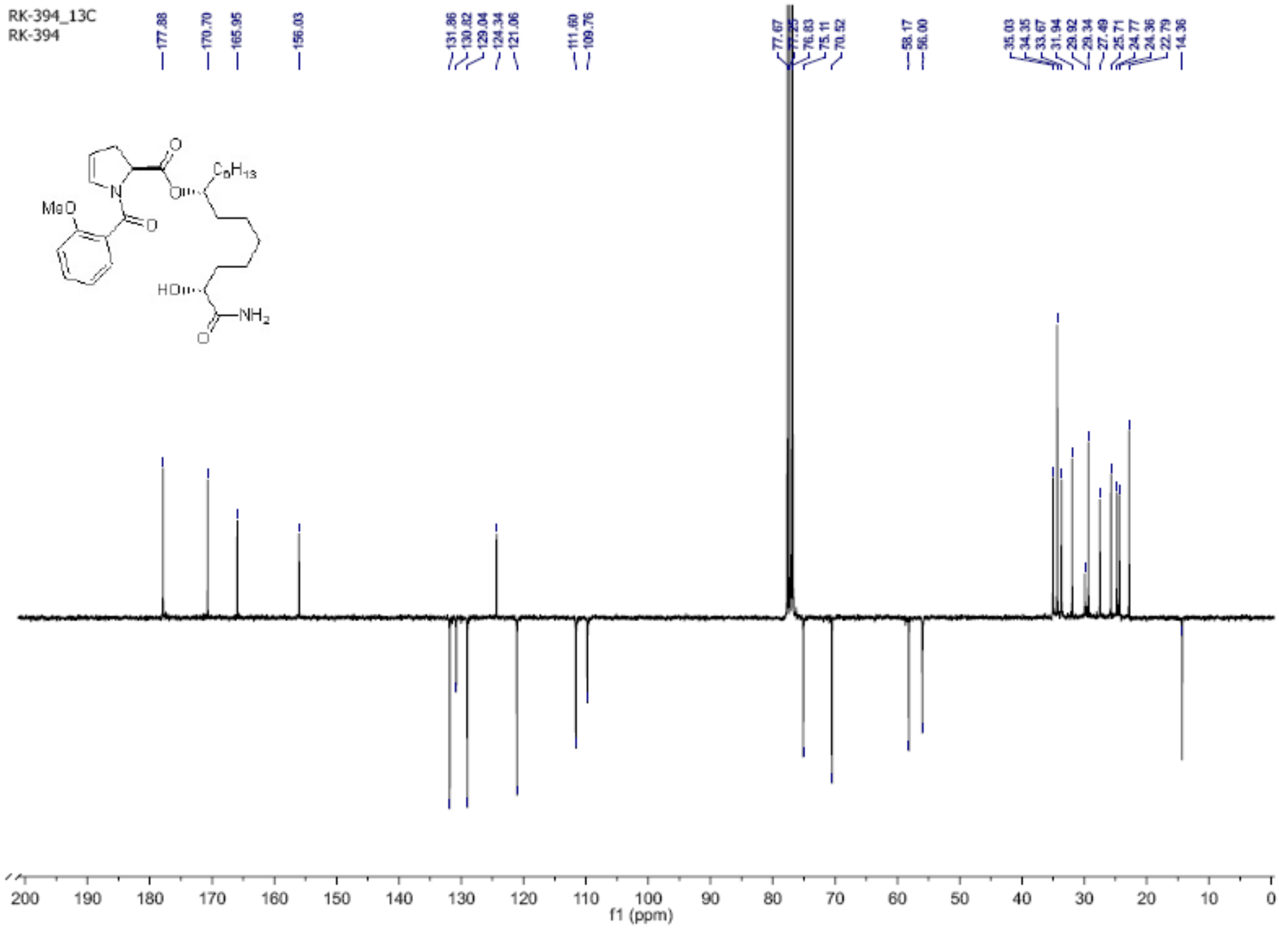

RK-394_13C
RK-394
MeO
C6H13
HO
NH2
f1 (ppm)

Compound S11

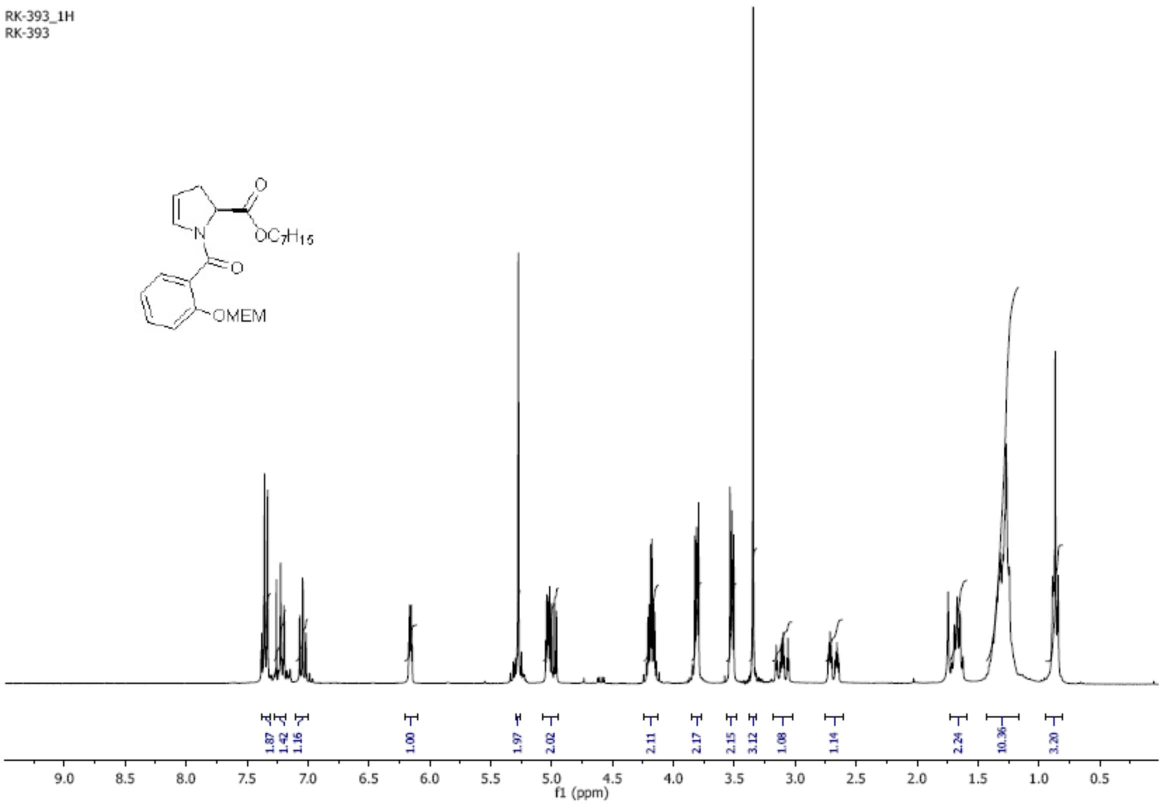

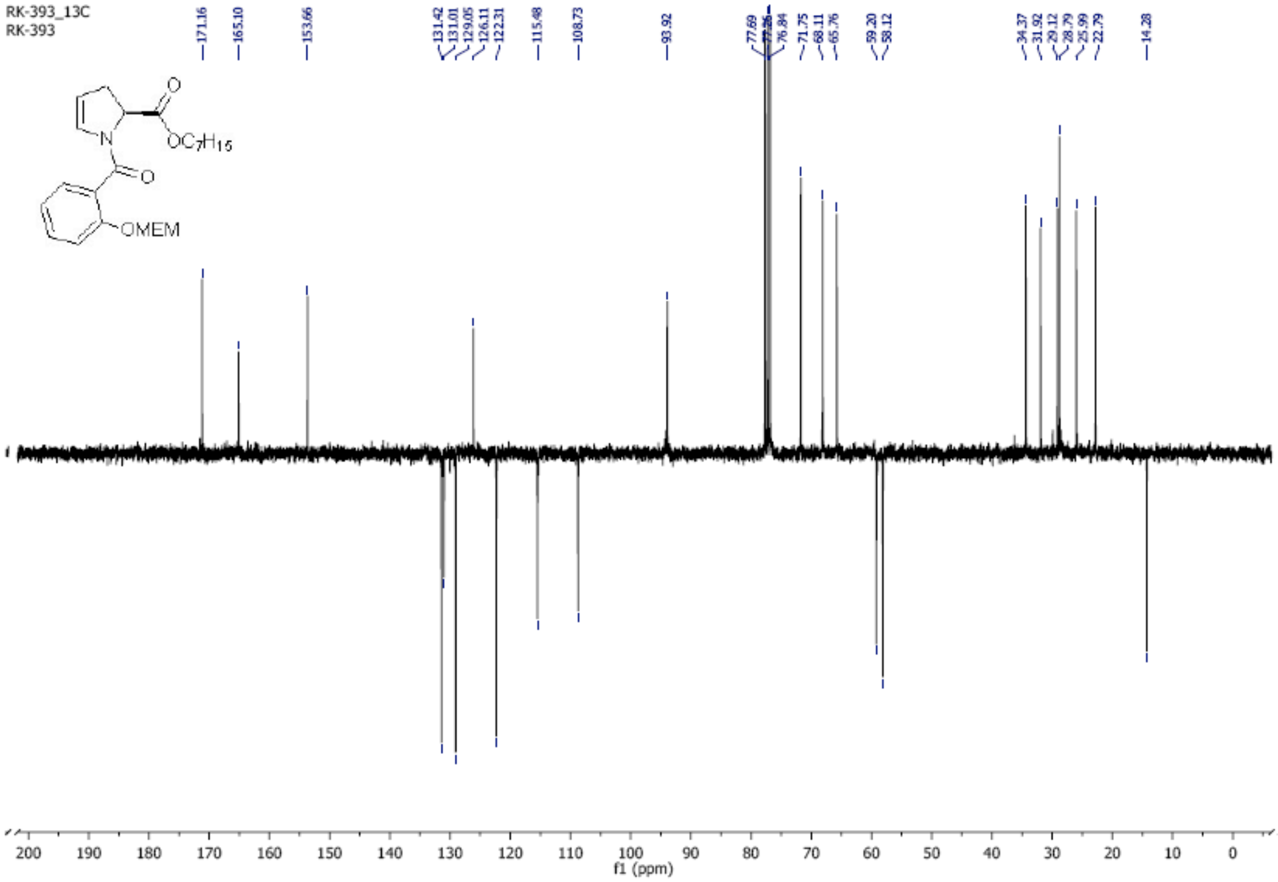

Compound 5

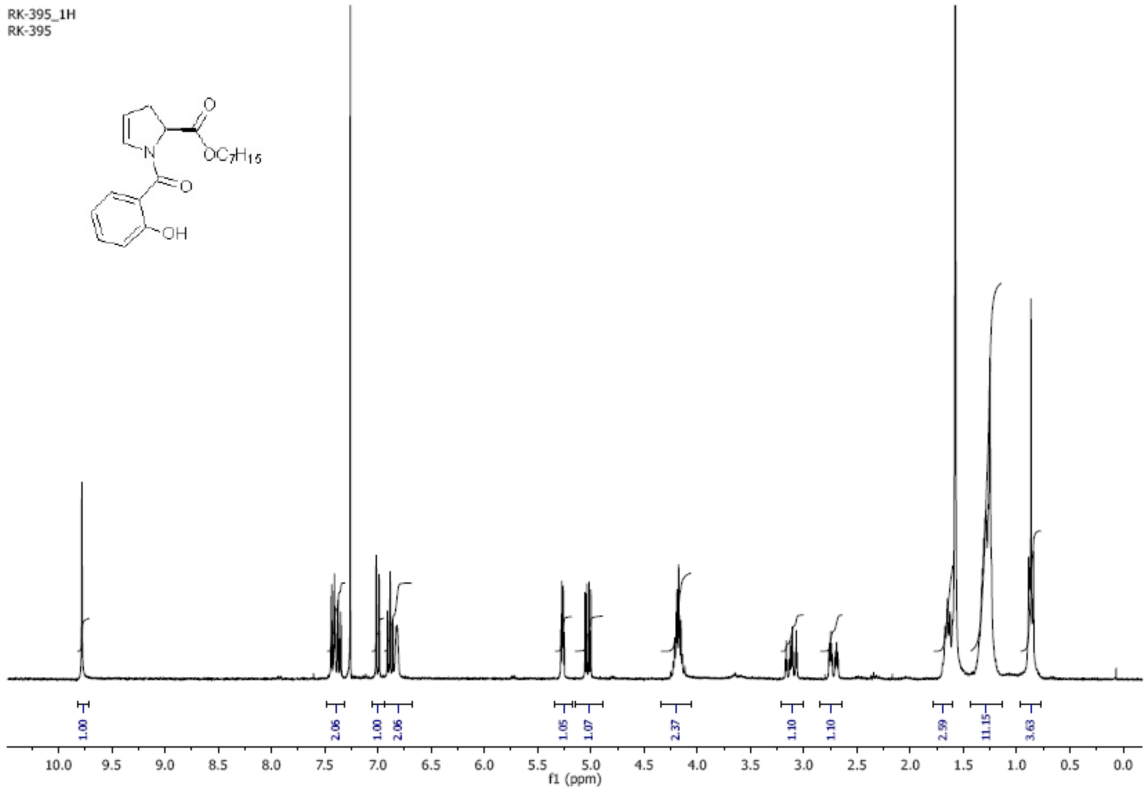
RK-395_1H
RK-395
OC7H15
OH
f1 (ppm)

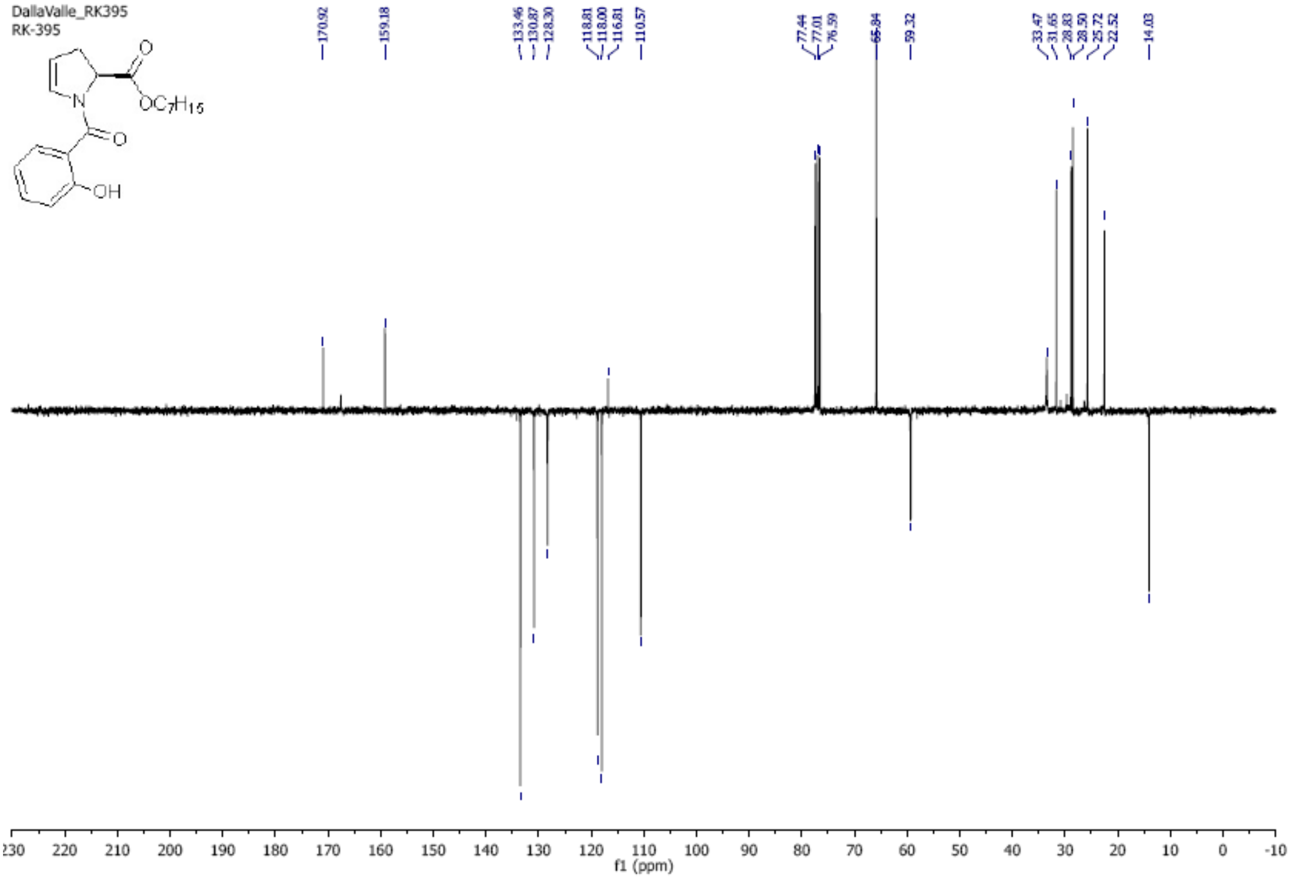
DallaValle_RK395
RK-395
170.92
159.18
133.46
130.87
128.30
118.81
118.00
116.81
110.57
77.44
77.01
76.59
59.32
33.47
31.65
28.83
28.50
25.72
22.52
14.03
OC7H15
OH
f1 (ppm)

Compound 6

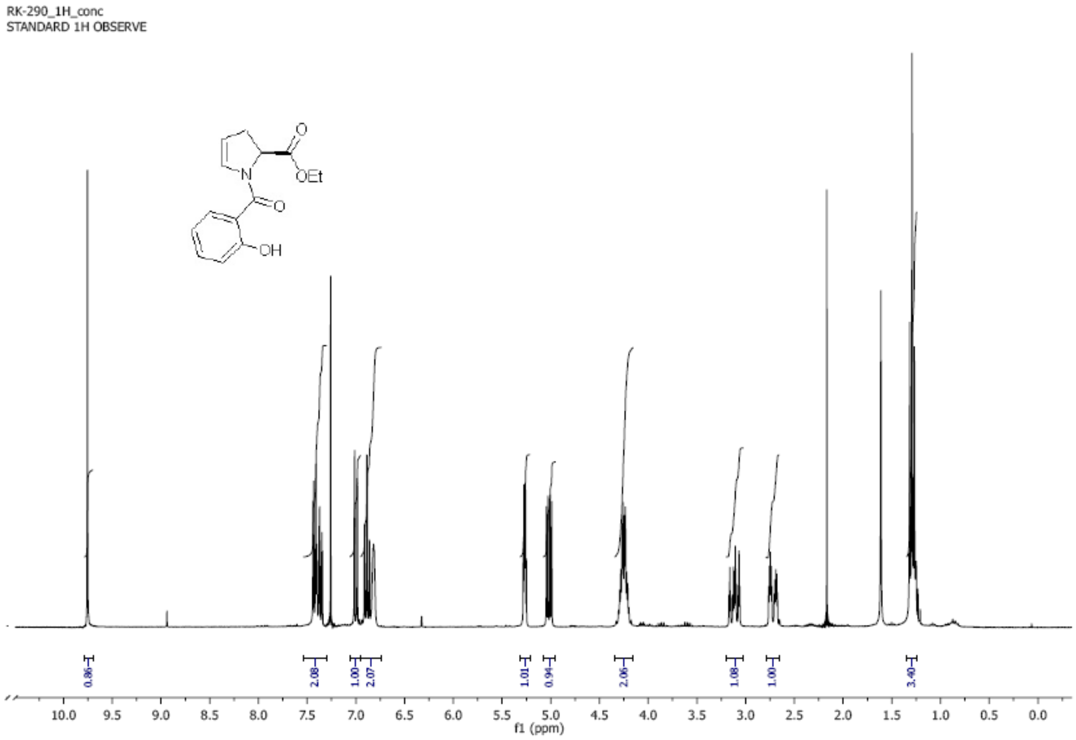
RK-290_1H_conc
STANDARD 1H OBSERVE
OEt
N
O
OH
0.86
2.08
1.00
2.07
1.01
0.94
2.06
1.08
1.00
3.40
10.0 9.5 9.0 8.5 8.0 7.5 7.0 6.5 6.0 5.5 5.0 4.5 4.0 3.5 3.0 2.5 2.0 1.5 1.0 0.5 0.0
f1 (ppm)

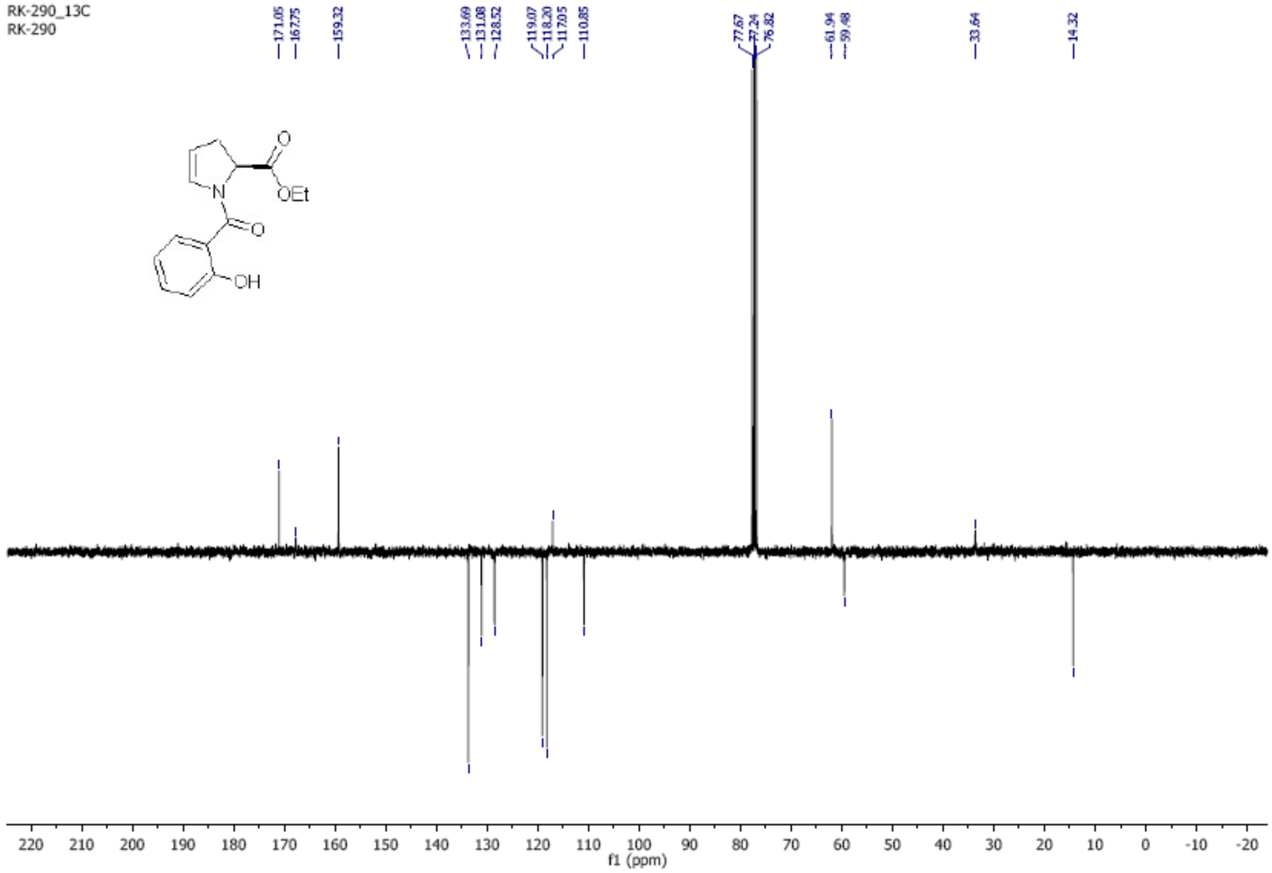
RK-290_13C
RK-290
171.05
167.75
159.32
133.69
131.08
128.52
119.07
118.20
117.05
110.85
77.67
77.24
76.82
61.94
59.48
33.64
14.32
OEt
N
O
OH
220 210 200 190 180 170 160 150 140 130 120 110 100 90 80 70 60 50 40 30 20 10 0 -10 -20
f1 (ppm)

Compound S13

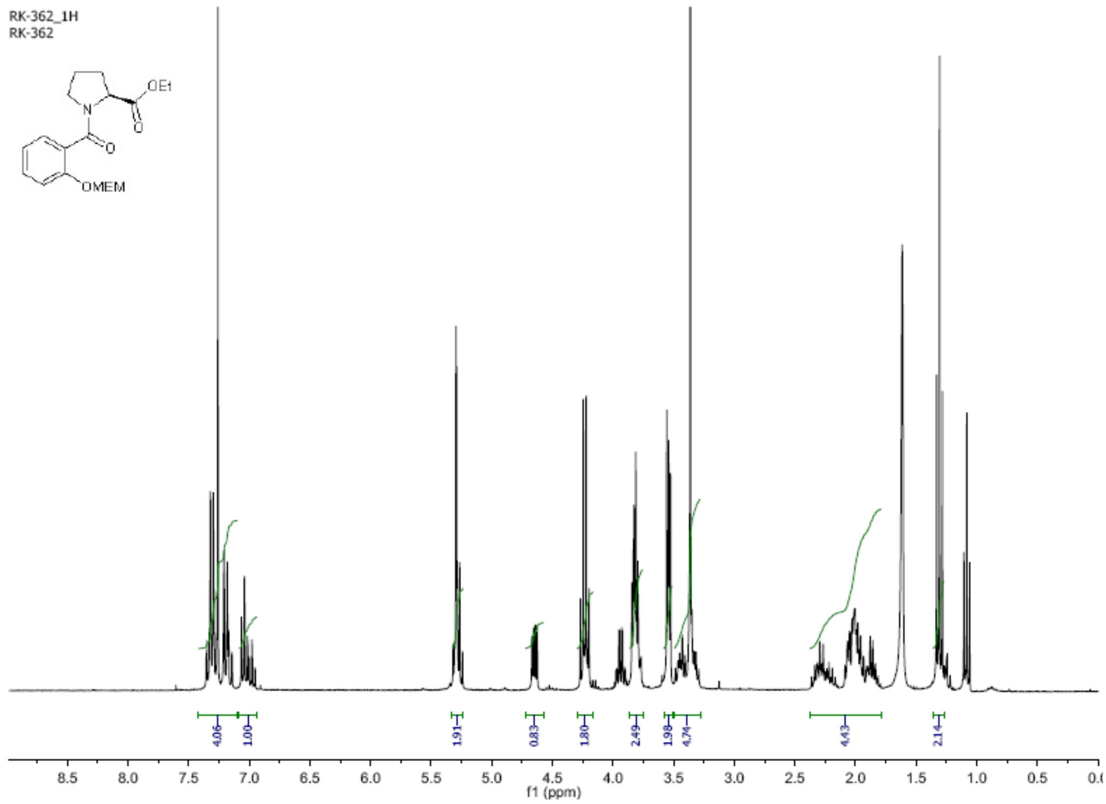

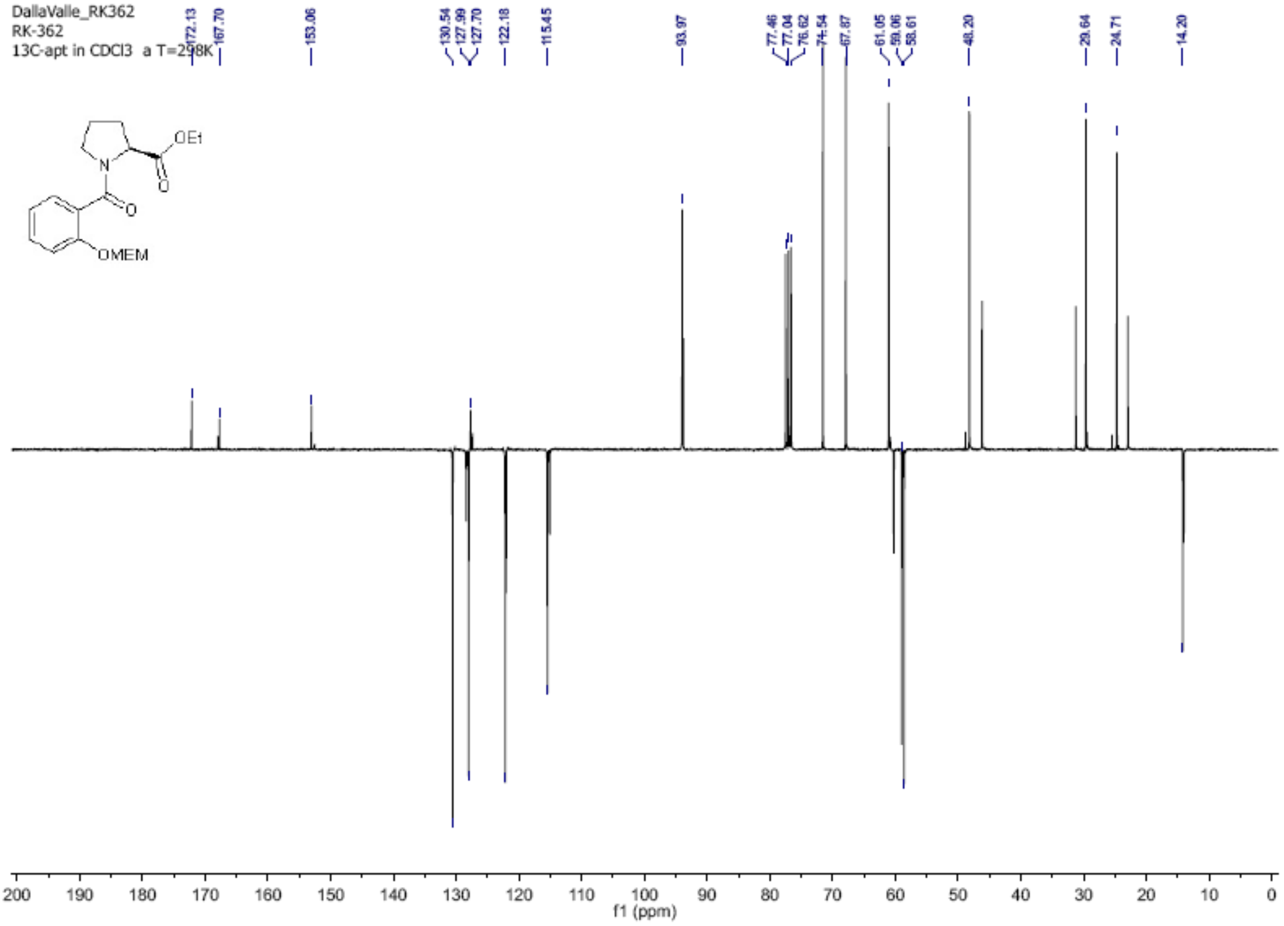

Compound S14

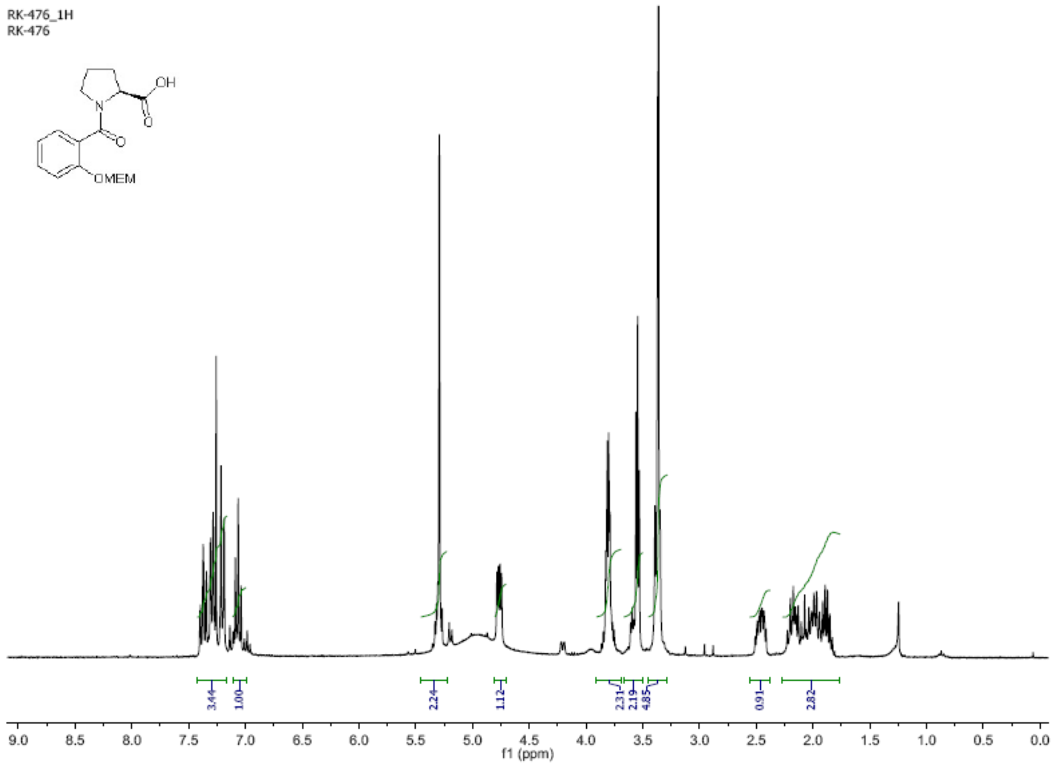

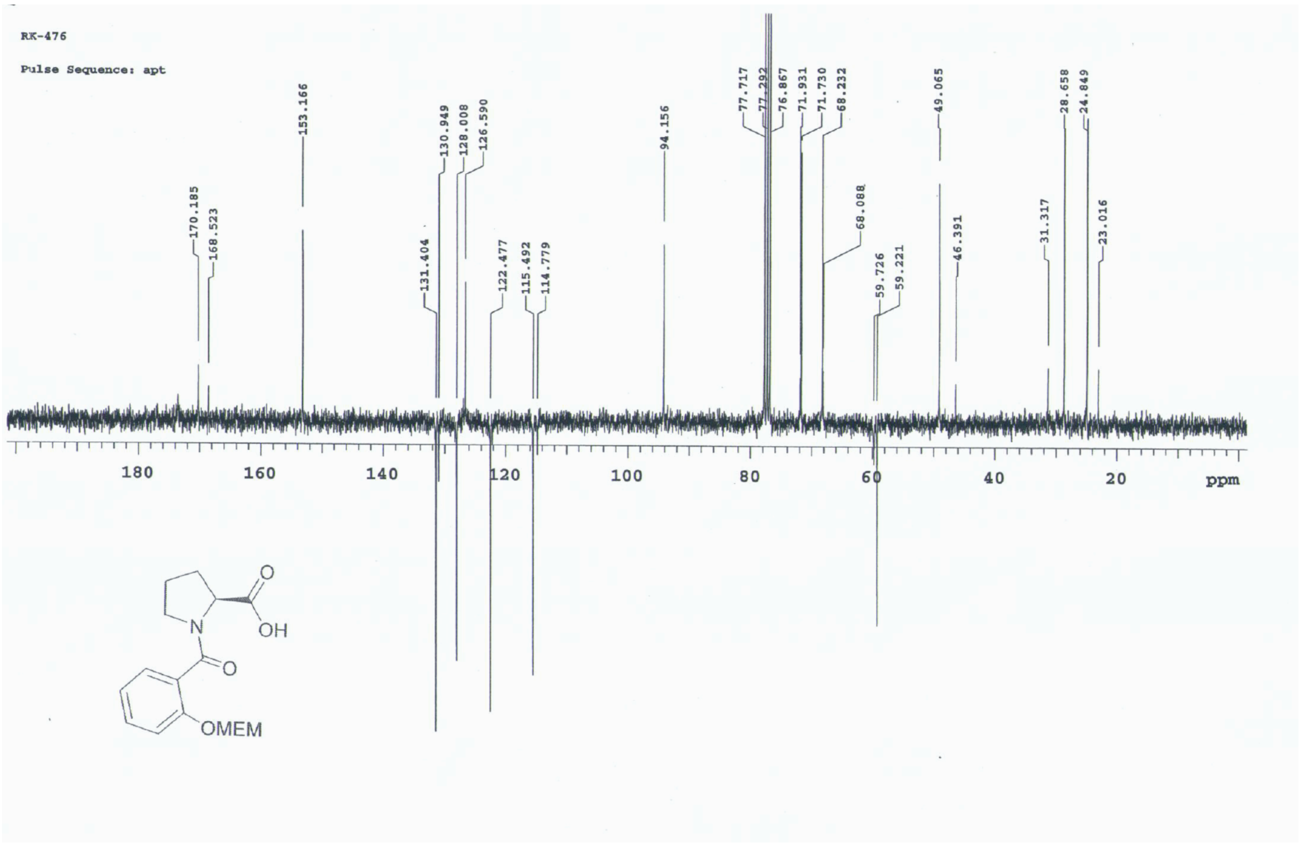

Compound S15

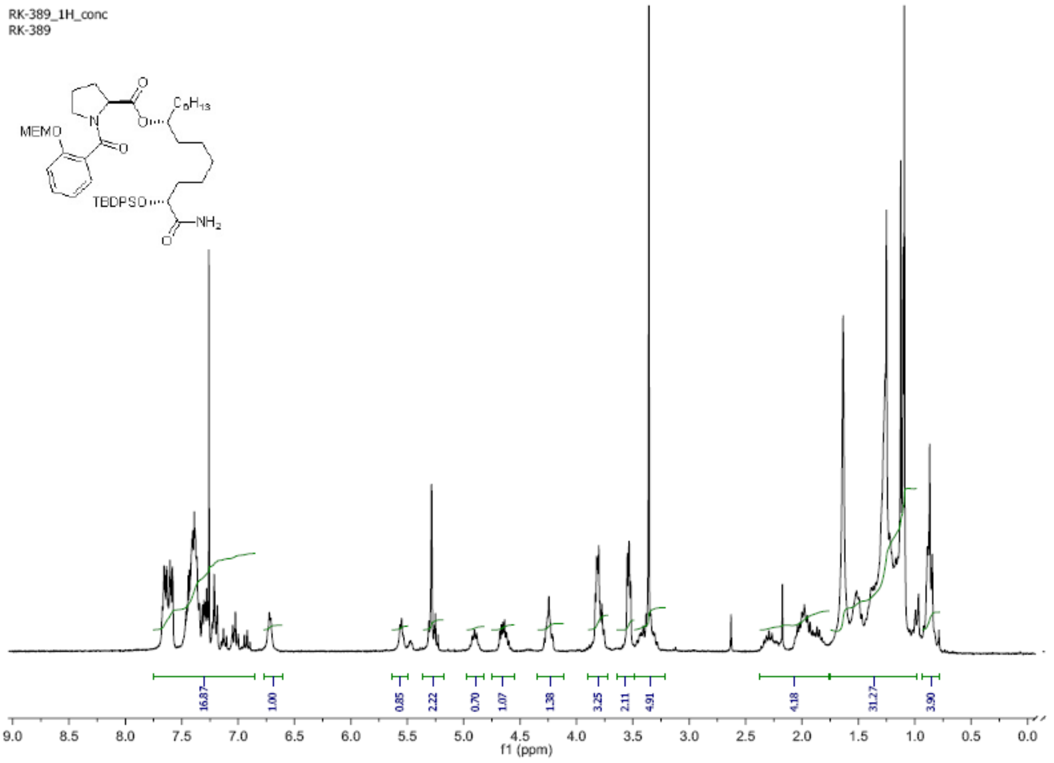

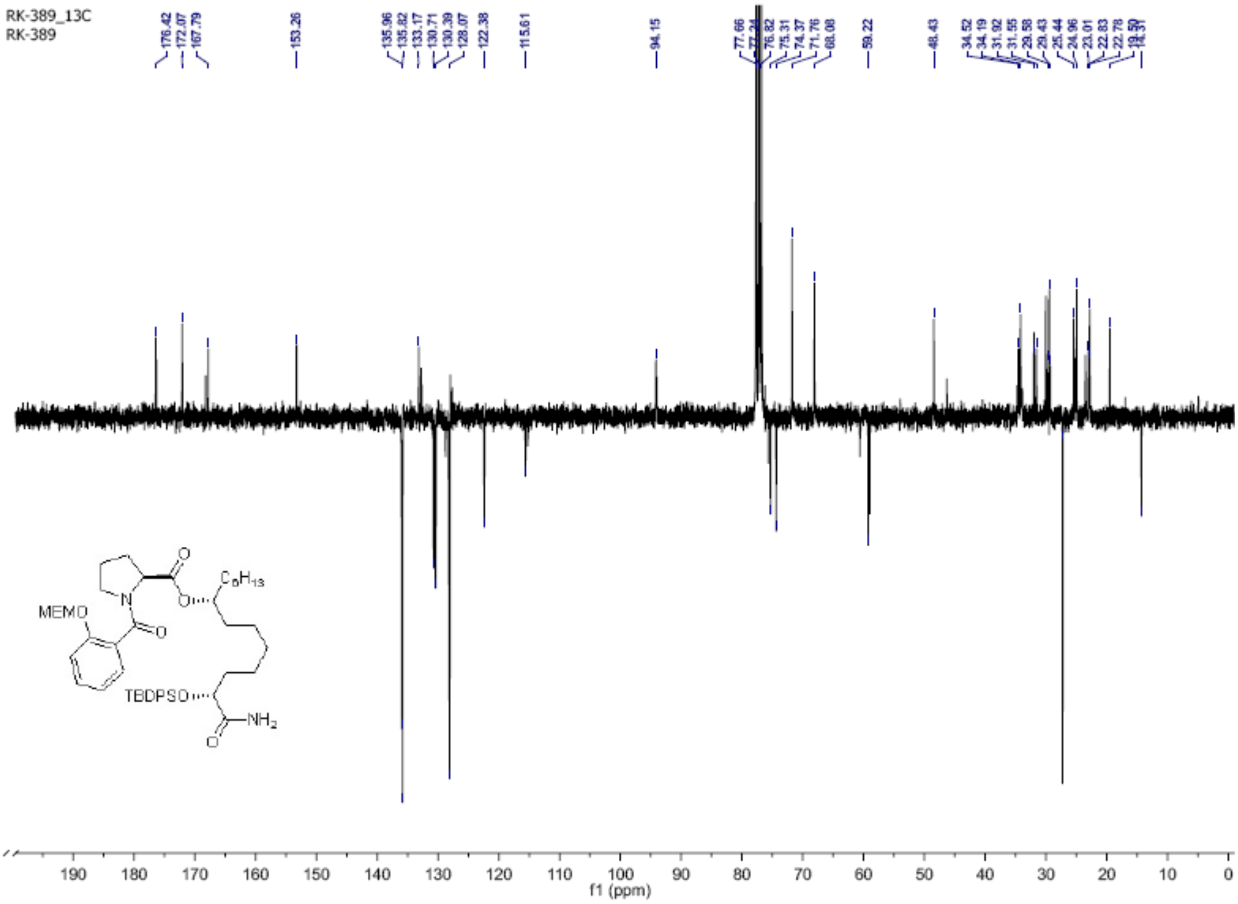

Compound 7

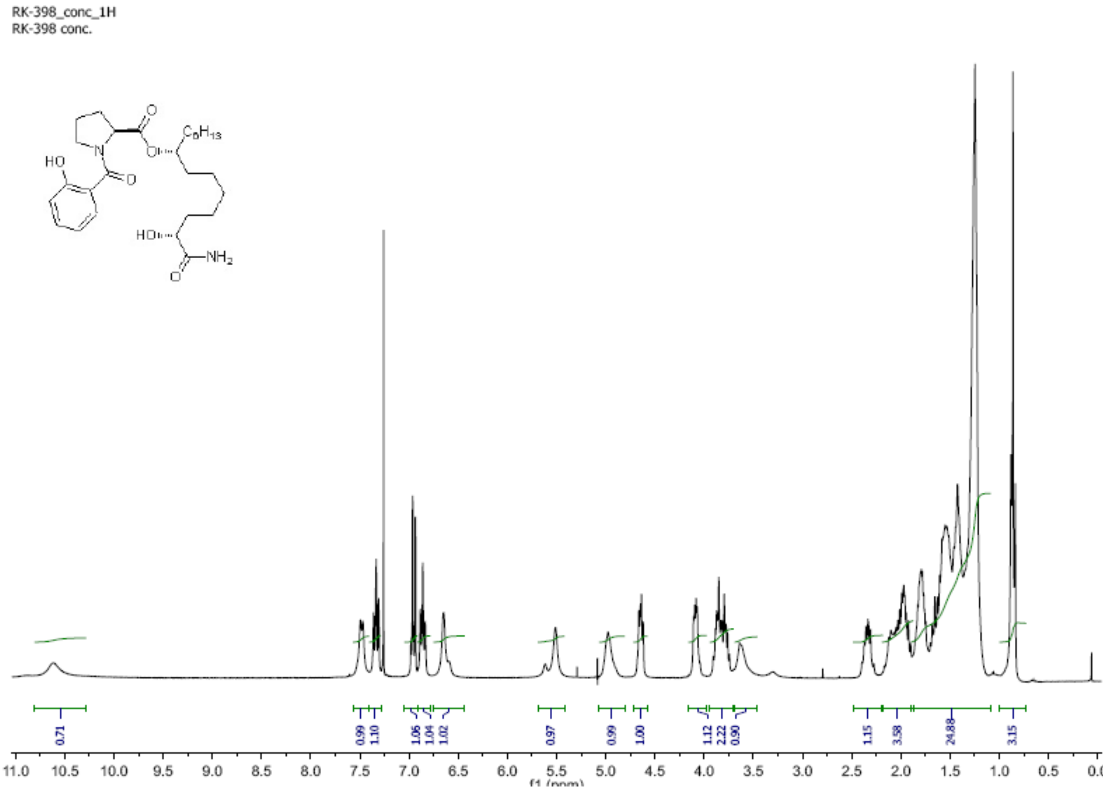
RK-398_conc_1H
RK-398 conc.
f1 (ppm)

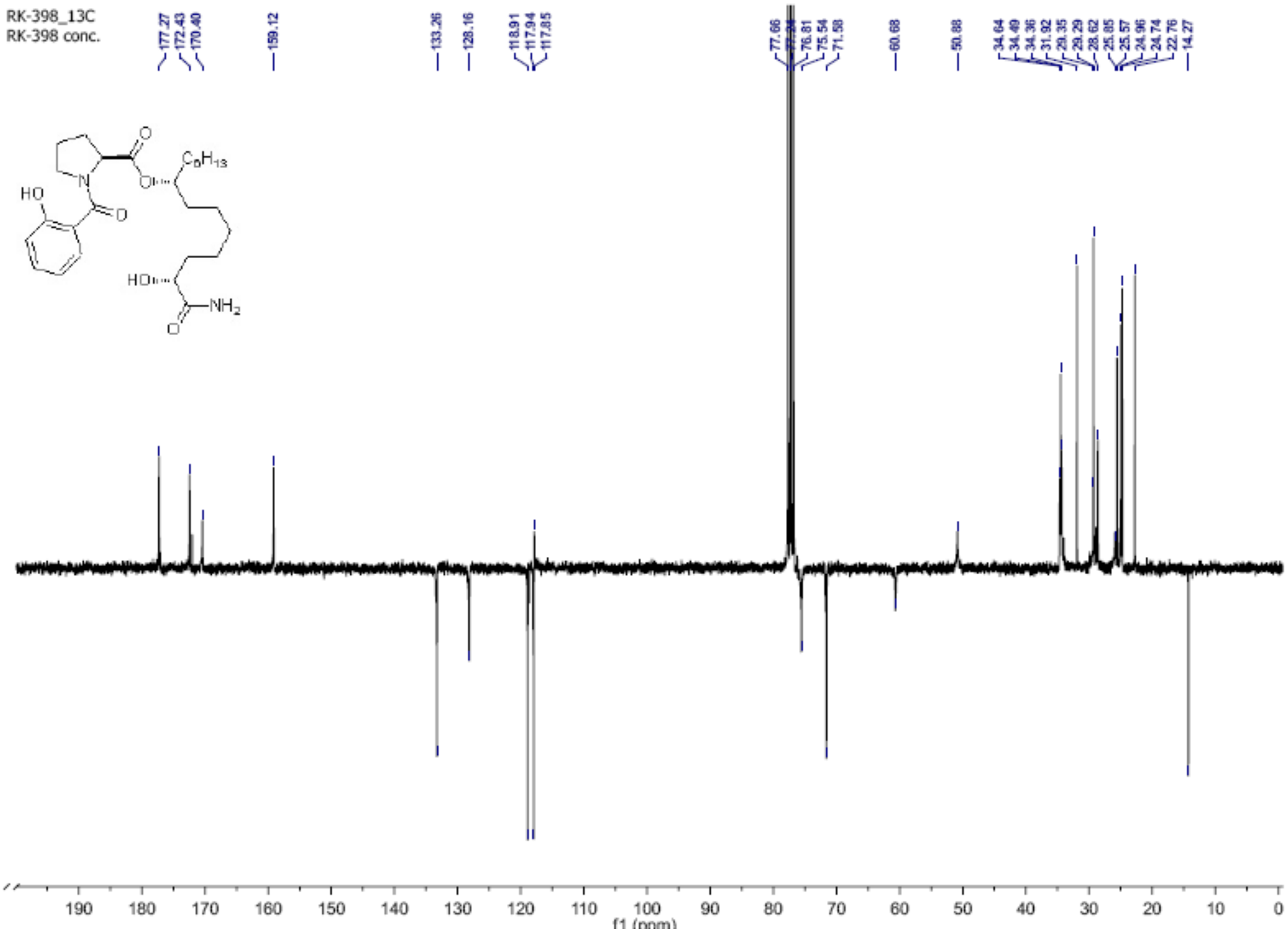
RK-398_13C
RK-398 conc.
177.27
172.43
170.40
159.12
133.26
128.16
118.91
117.94
117.85
77.66
76.81
75.54
71.58
60.68
50.88
34.64
34.49
34.36
31.92
29.35
29.29
28.62
25.85
25.57
24.96
24.74
22.76
14.27
f1 (ppm)

Compound 8

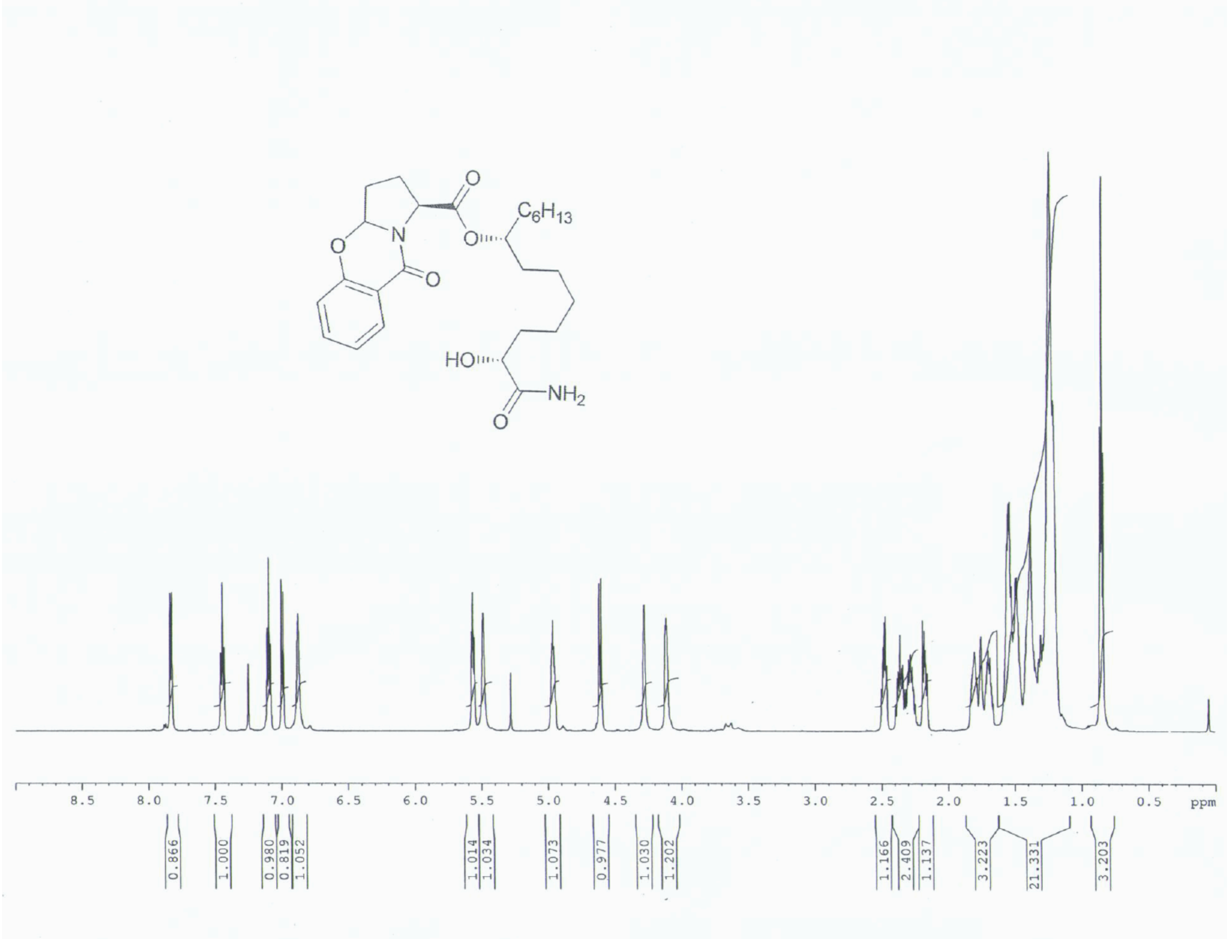

O
N
O
O
O
C_6H_{13}
HO
NH_2
O
8.5 8.0 7.5 7.0 6.5 6.0 5.5 5.0 4.5 4.0 3.5 3.0 2.5 2.0 1.5 1.0 0.5 ppm
0.866 1.000 0.980 0.819 1.052 1.014 1.034 1.073 0.977 1.030 1.202 1.166 2.409 1.137 3.223 21.331 3.203

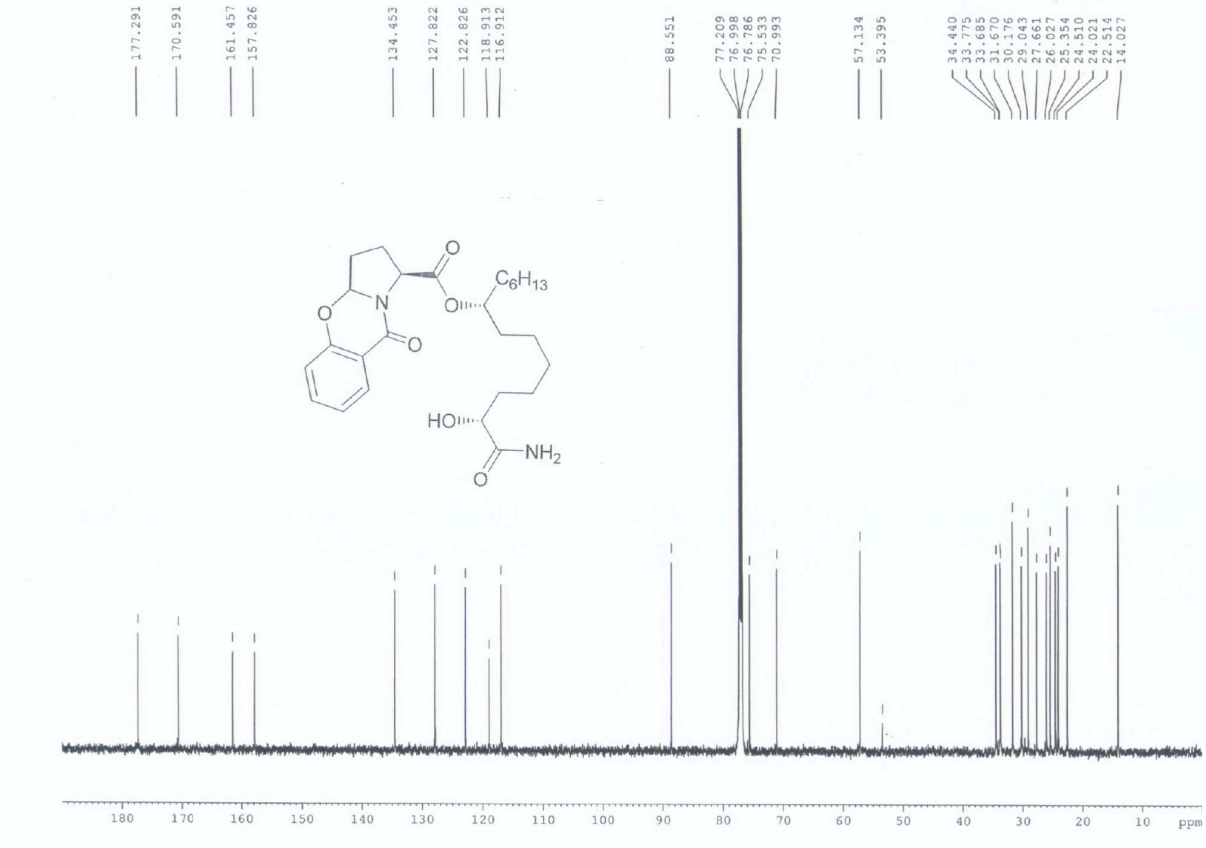

177.291 170.591 161.457 157.826 134.453 127.822 122.826 118.913 116.912 88.551 77.209 76.998 76.786 75.533 70.993 57.134 53.395 34.440 33.775 33.685 31.670 30.176 29.043 27.661 26.027 25.354 24.510 24.021 22.514 14.027
O
N
O
O
O
C_6H_{13}
HO
NH_2
O
180 170 160 150 140 130 120 110 100 90 80 70 60 50 40 30 20 10 ppm

Compound 9

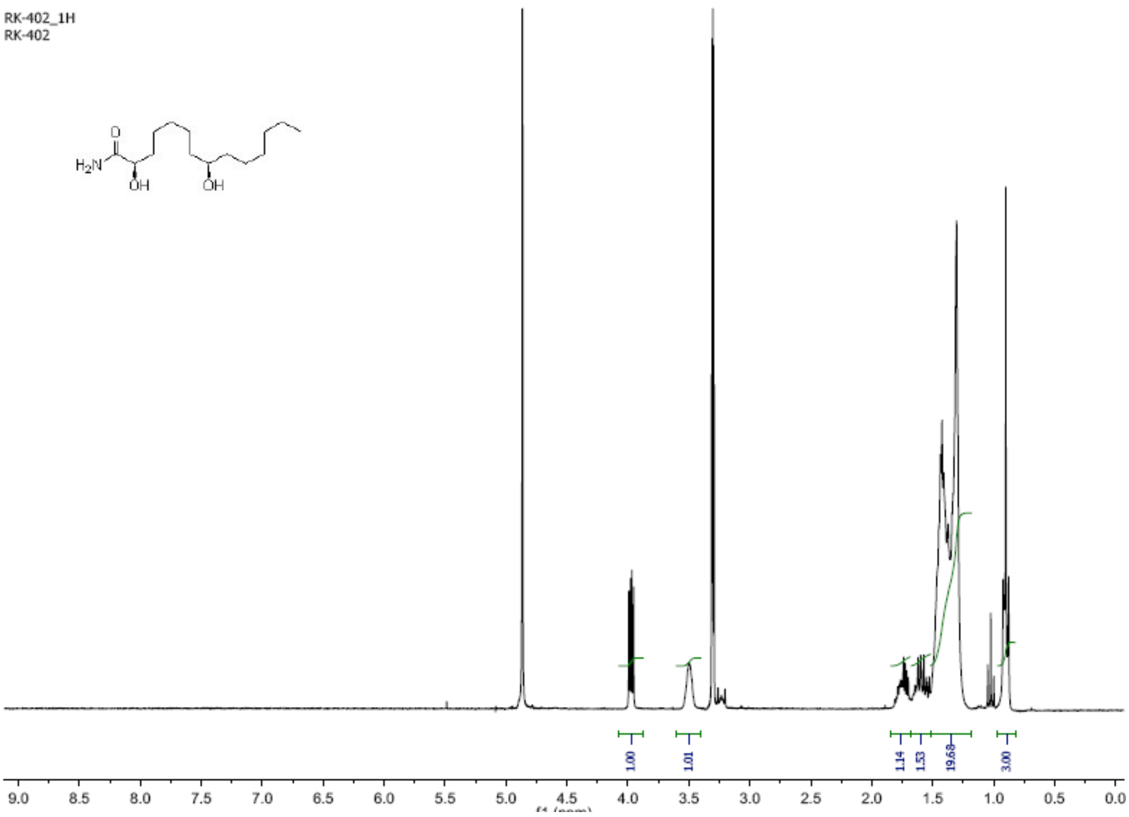
RK-402_1H
RK-402
1.00
1.01
1.14
1.53
19.68
3.00
f1 (ppm)

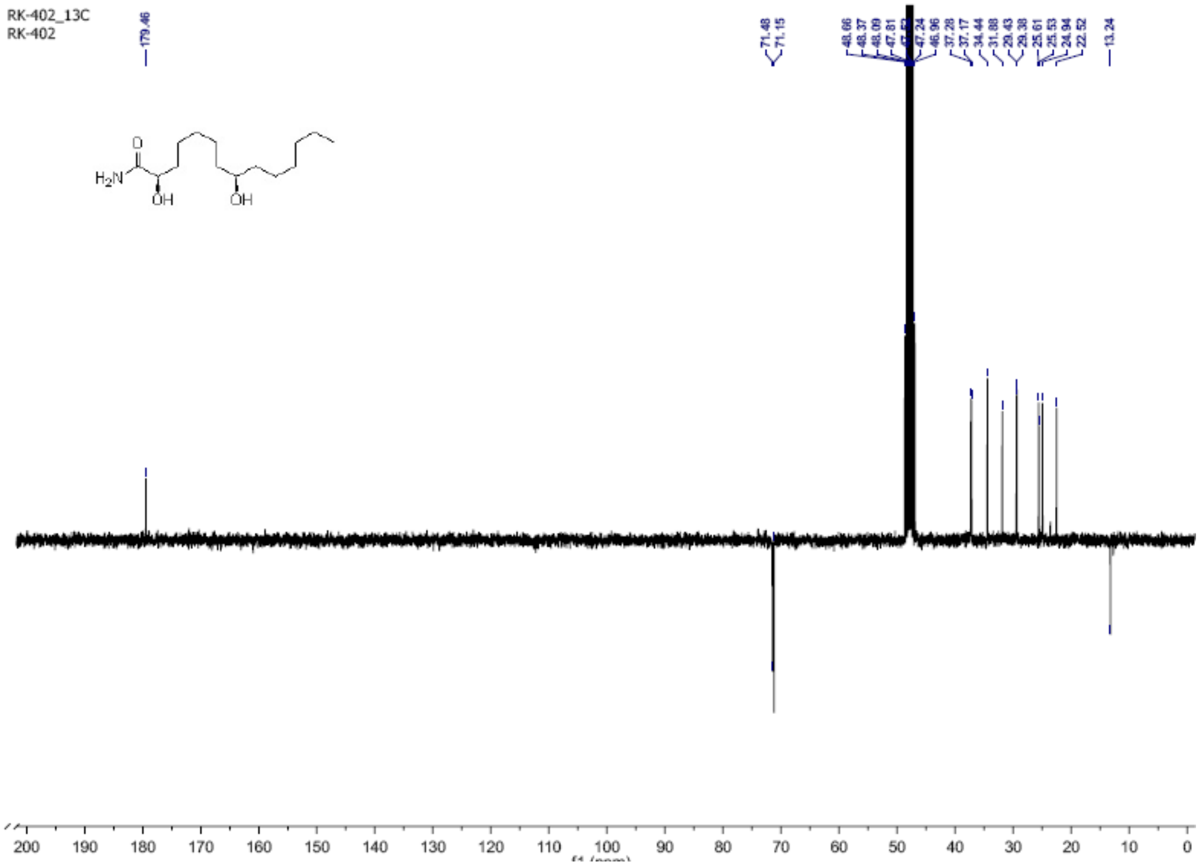
RK-402_13C
RK-402
179.46
71.48
71.15
48.66
48.37
48.09
47.81
47.24
46.96
37.28
37.17
34.44
31.88
29.43
29.38
25.61
25.53
24.94
22.52
13.24
f1 (ppm)

Compound 10

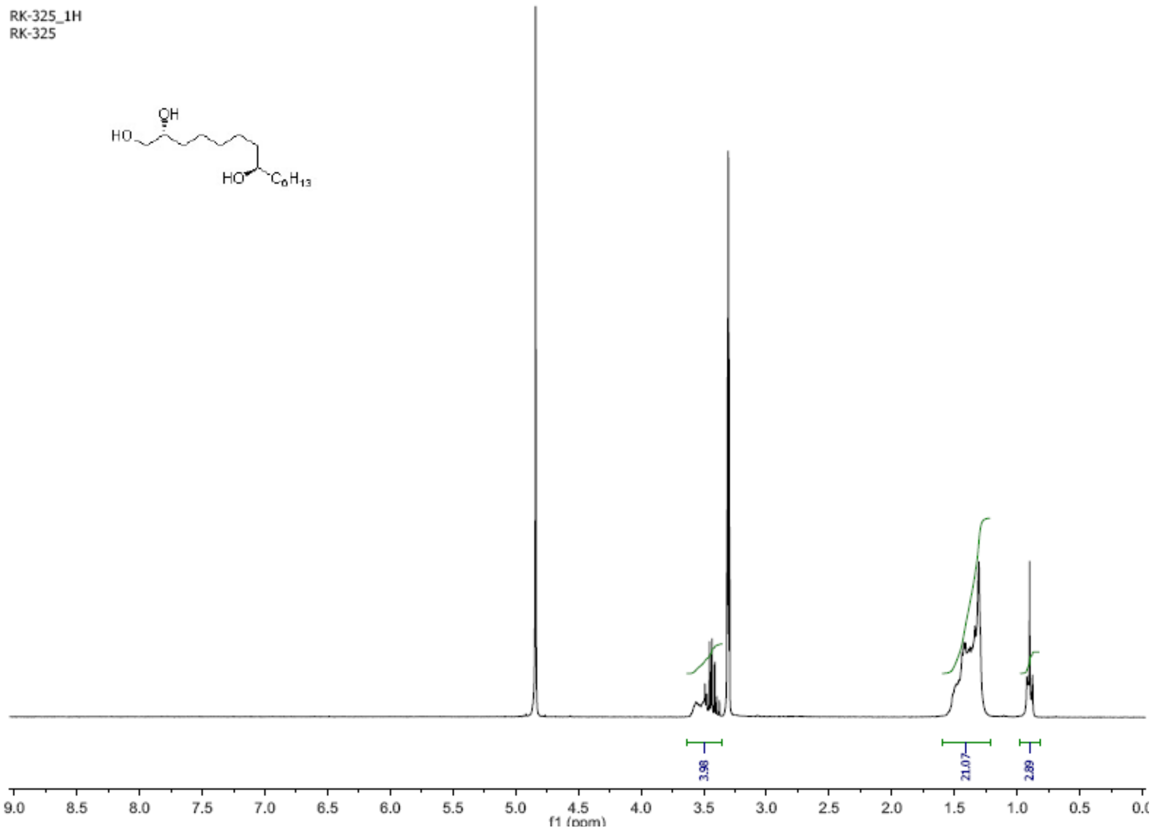

RK-325_1H
RK-325
3.98
21.07
2.89
f1 (ppm)

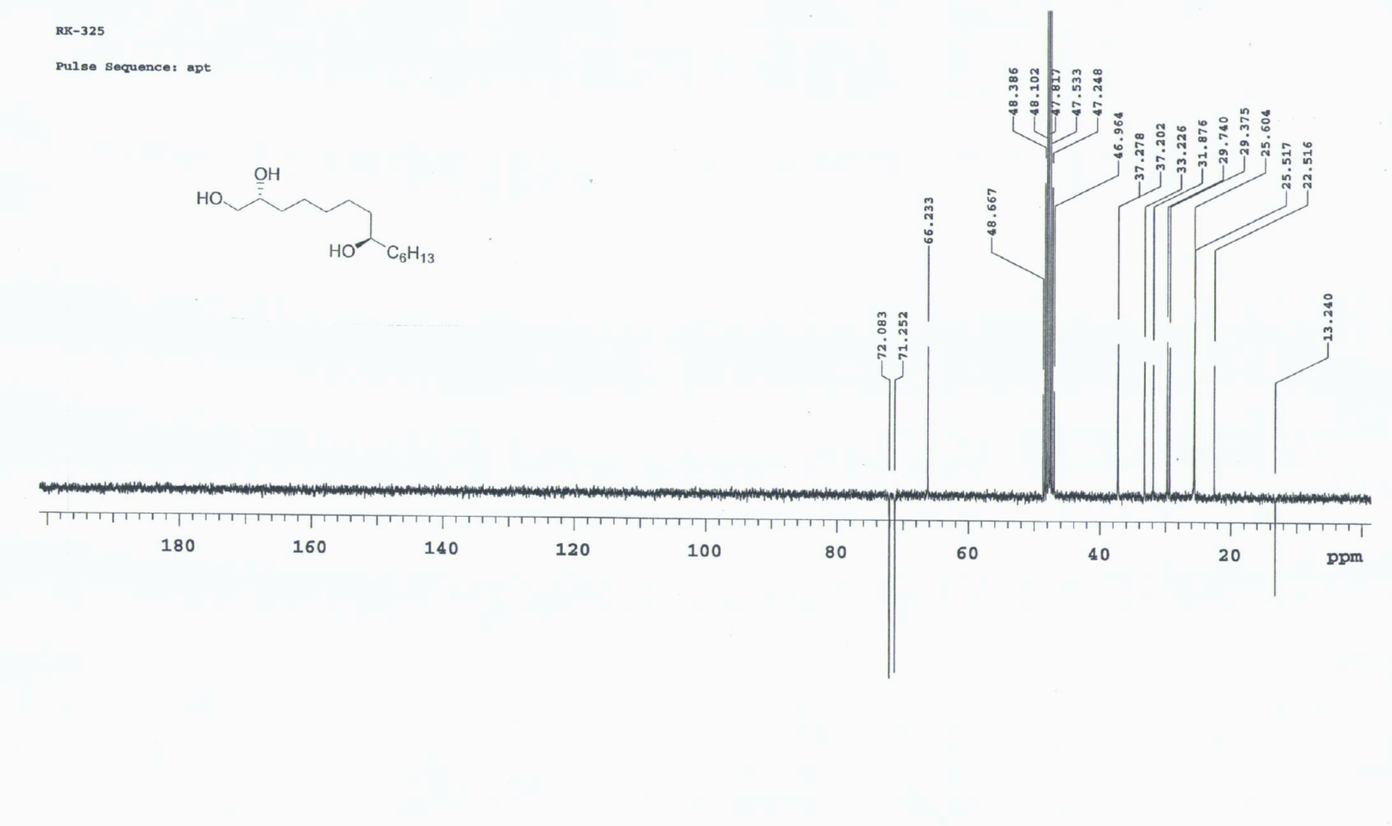

RK-325
Pulse Sequence: apt
72.083
71.252
66.233
48.667
48.386
48.102
47.817
47.533
47.248
46.964
37.278
37.202
33.226
31.876
29.740
29.375
25.604
25.517
22.516
13.240
ppm

Compound S18

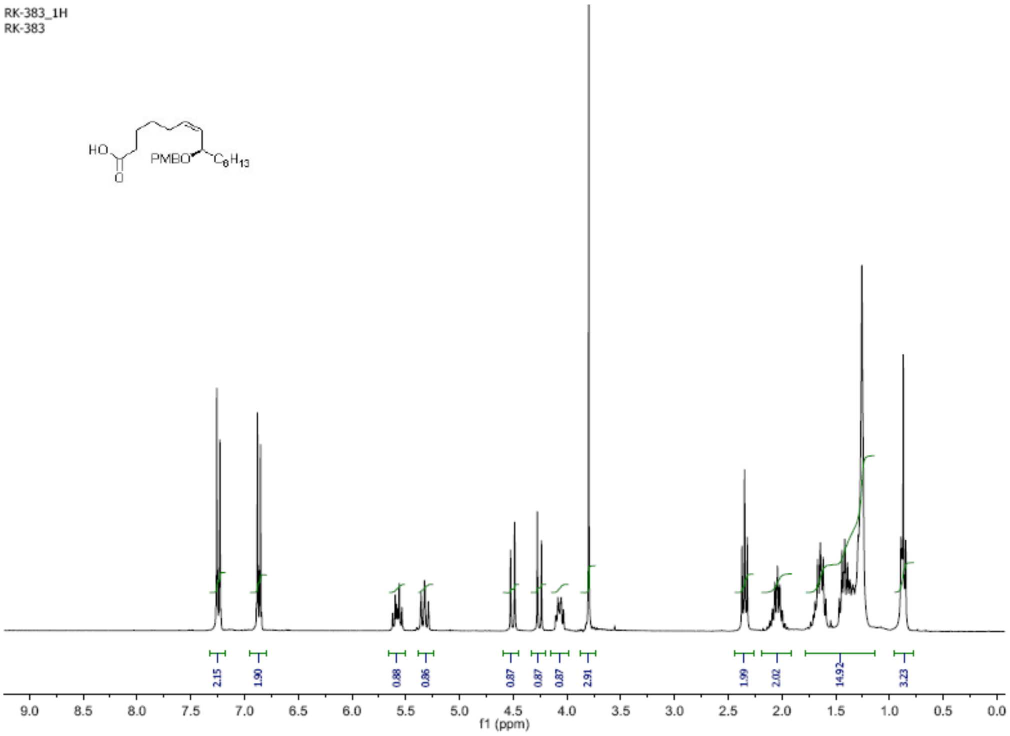

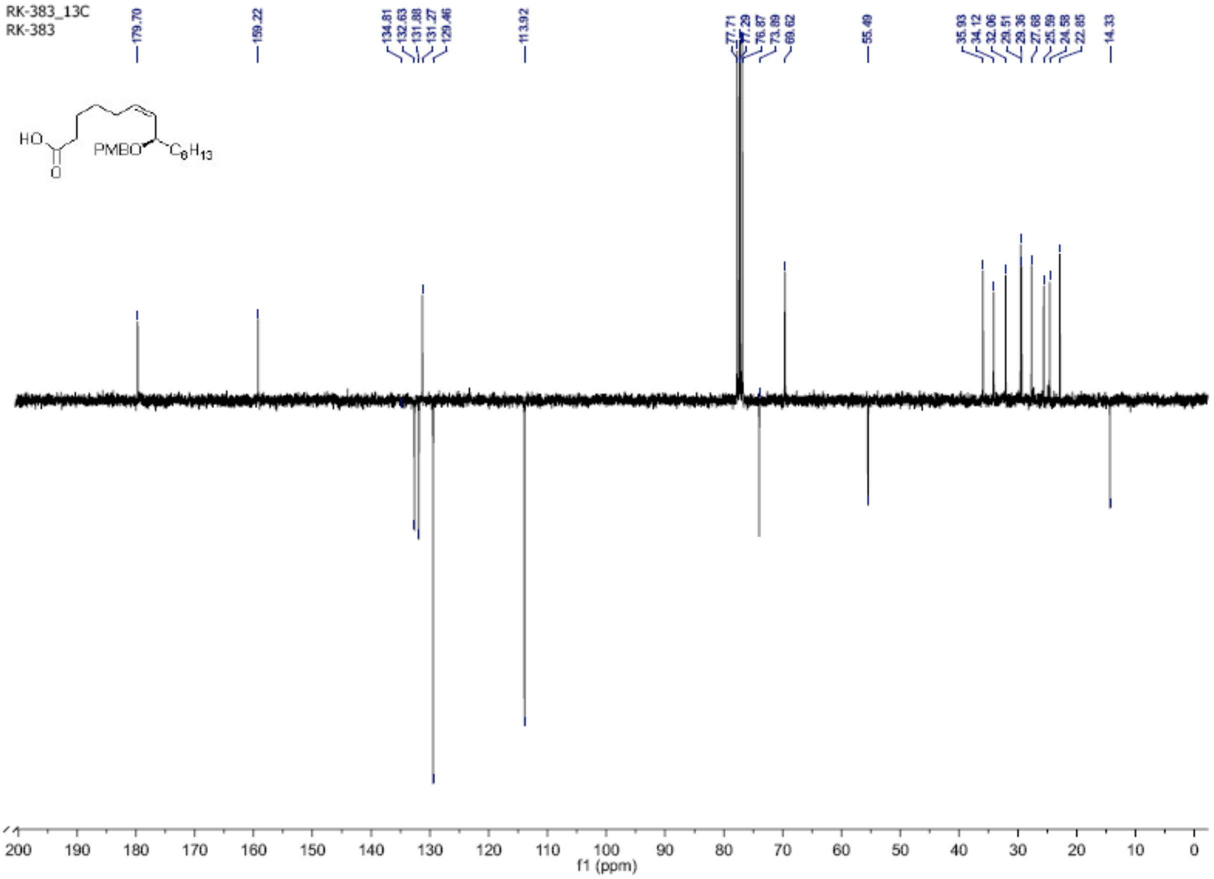

Compound S19

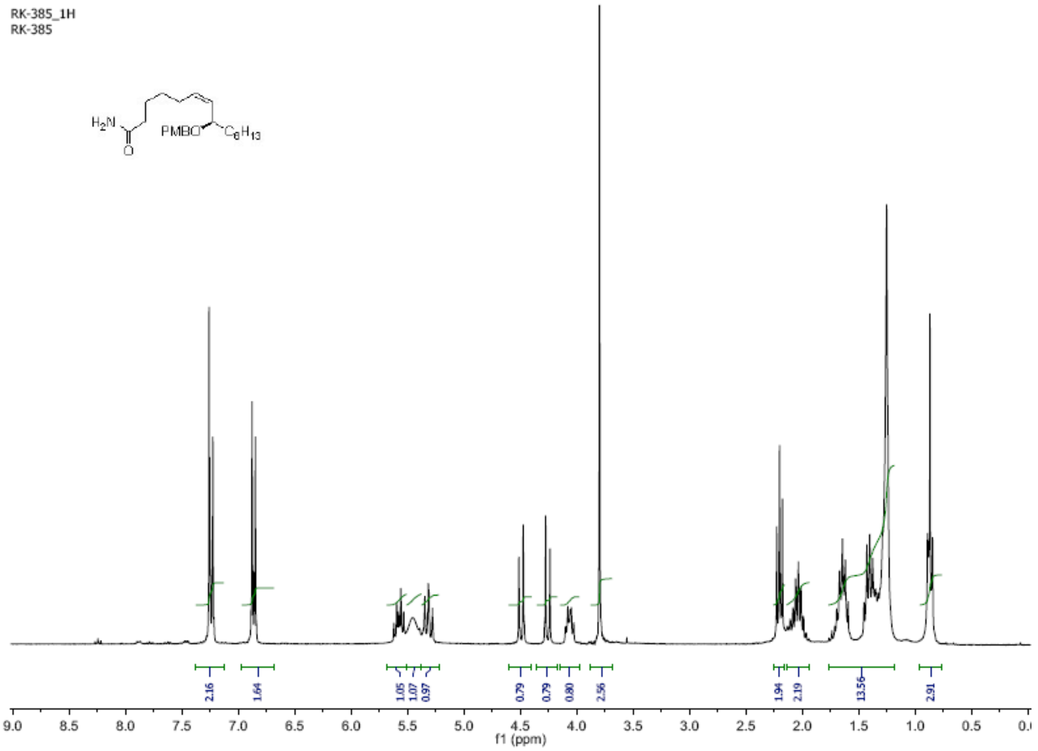

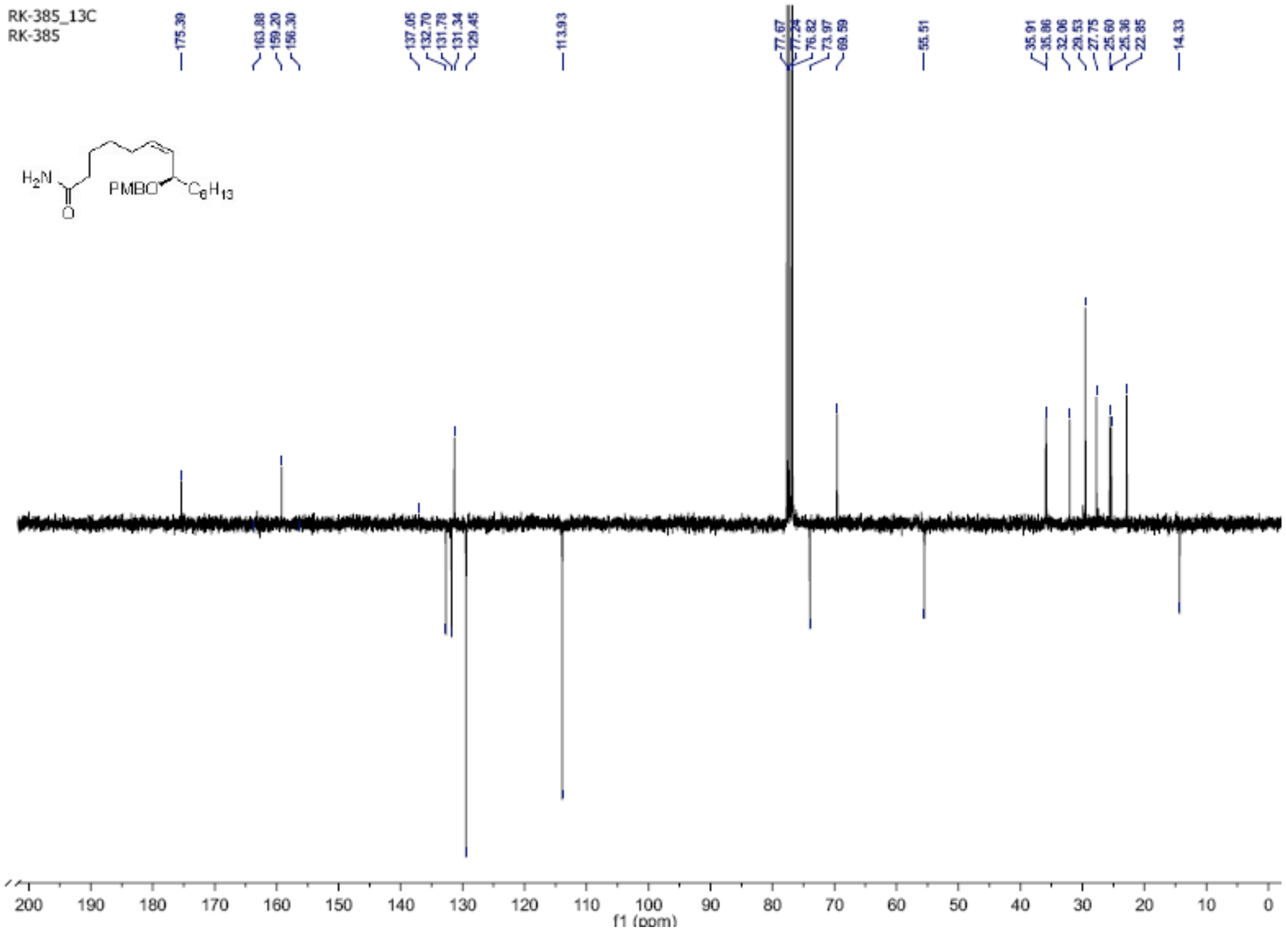

Compound 11

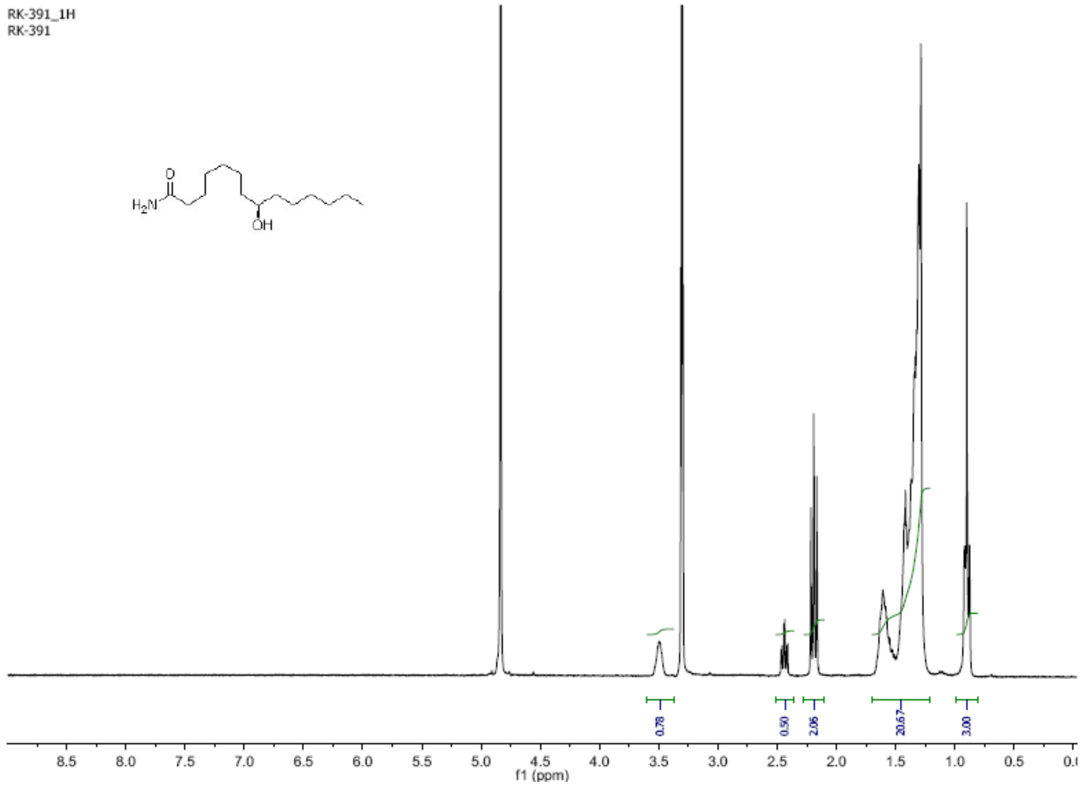
RK-391_1H
RK-391
H2N
O
OH
0.78
0.50
2.06
20.67
3.00
f1 (ppm)

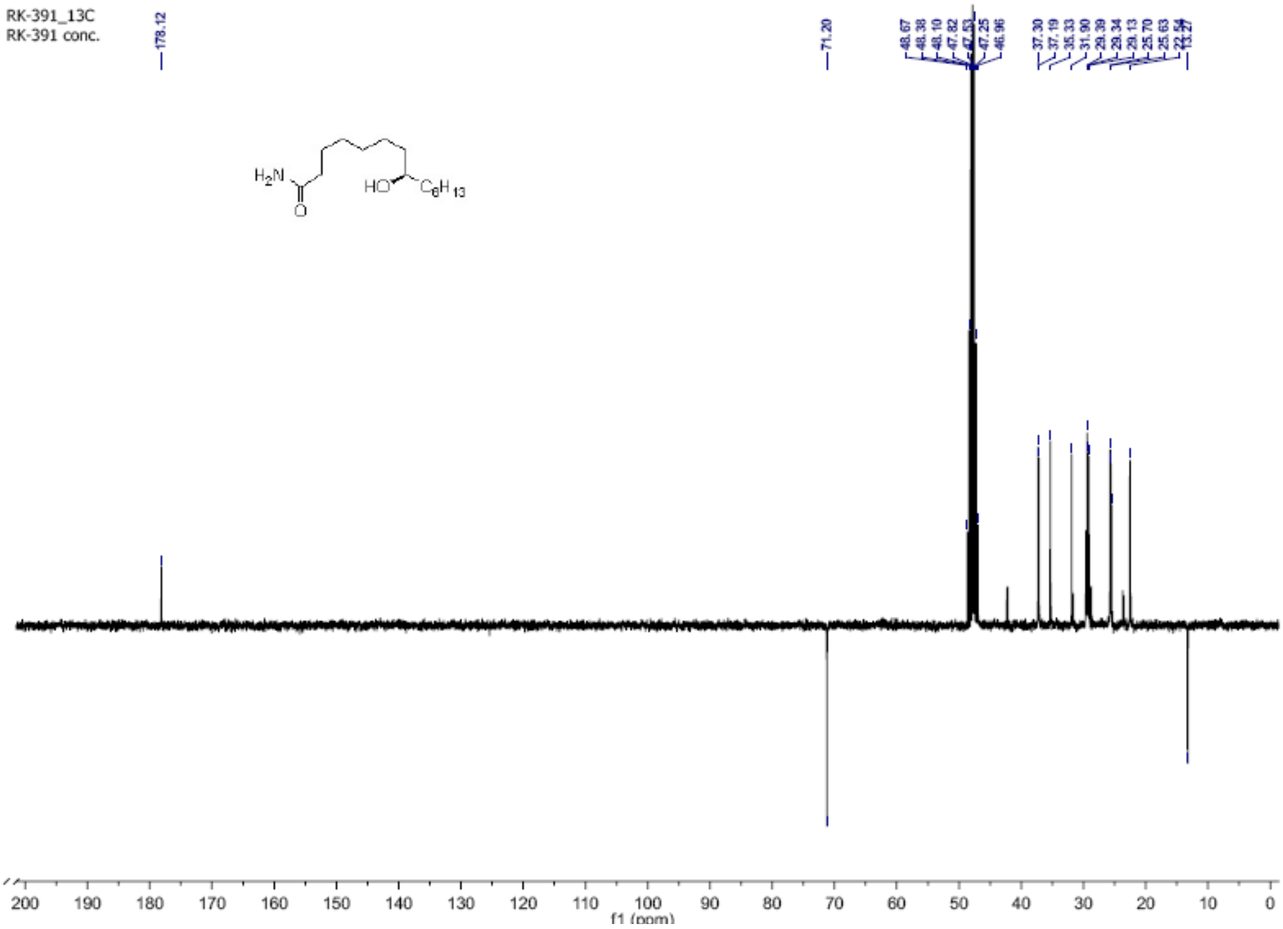
RK-391_13C
RK-391 conc.
178.12
71.20
48.67
48.38
48.10
47.82
47.53
47.25
46.96
37.30
37.19
35.33
31.90
29.39
29.34
29.13
25.70
25.63
22.54
13.27
H2N
O
HO
C6H13
f1 (ppm)

Mass Spectra

Compound 1.

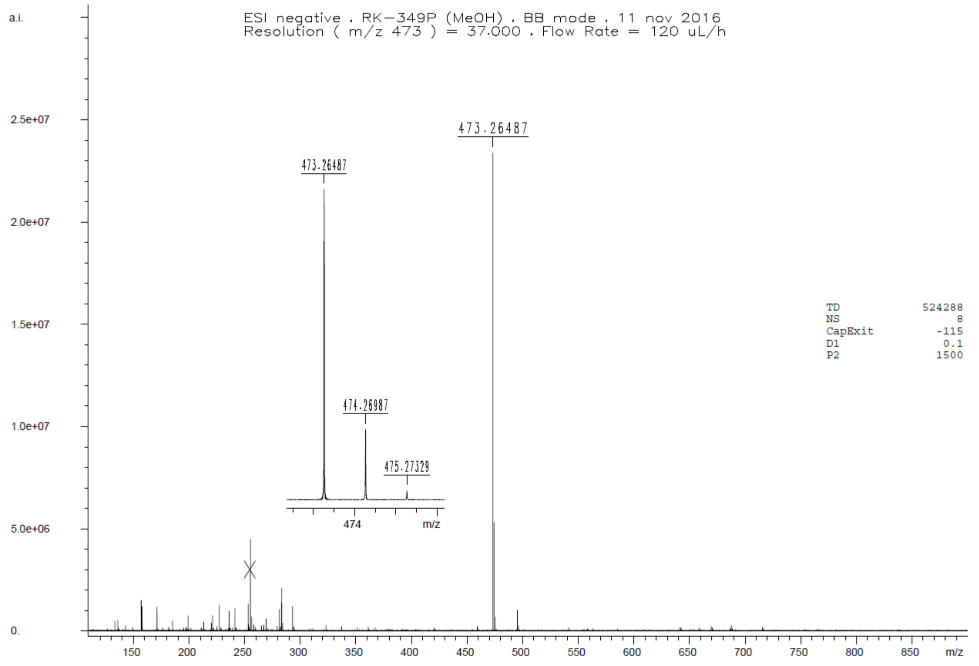

Compound 2.

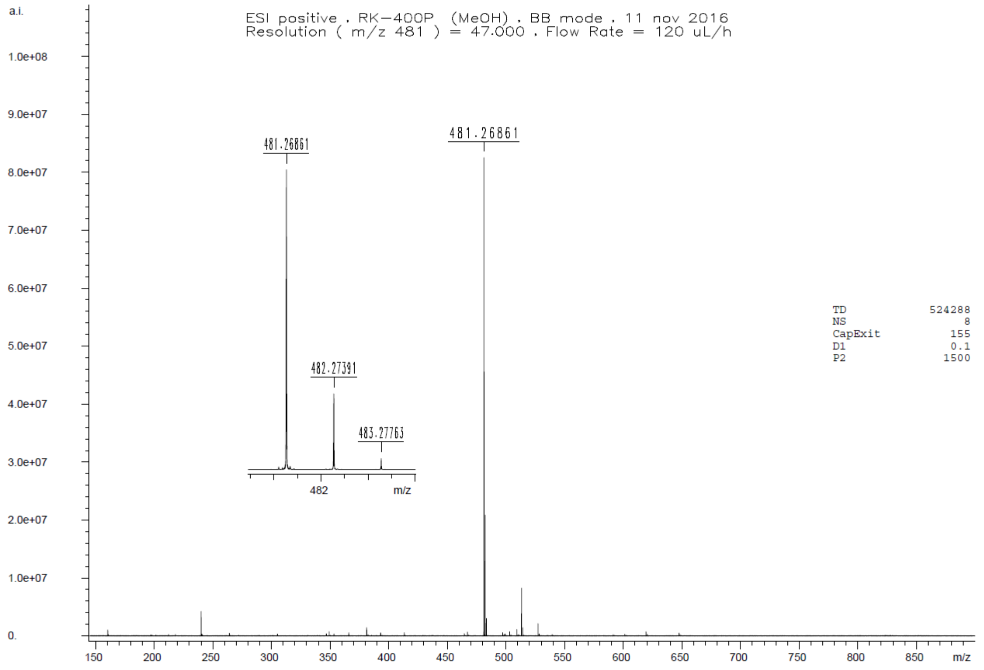

Compound 3.

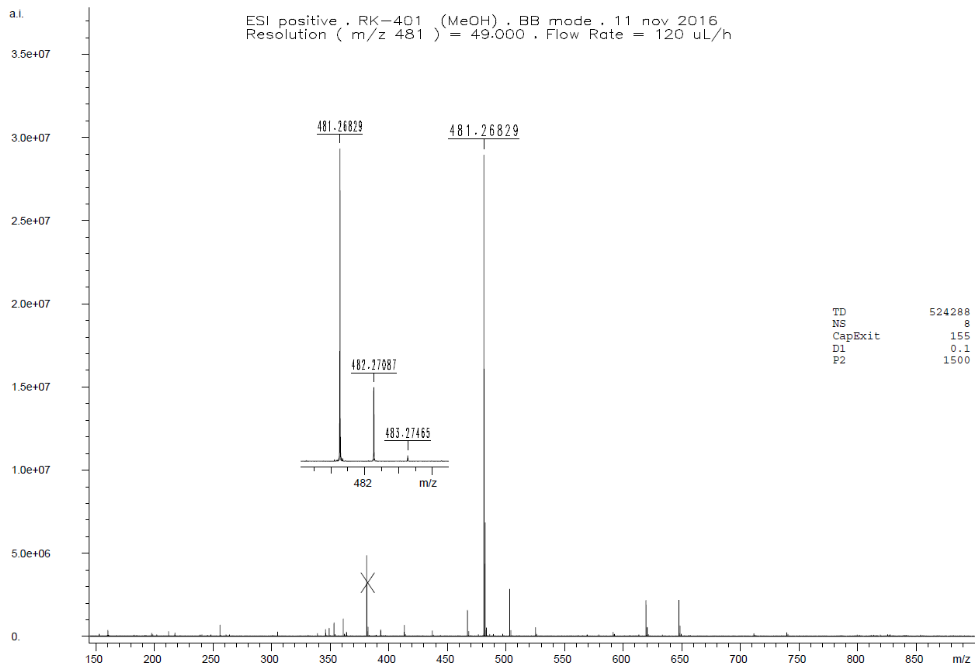

Compound 4.

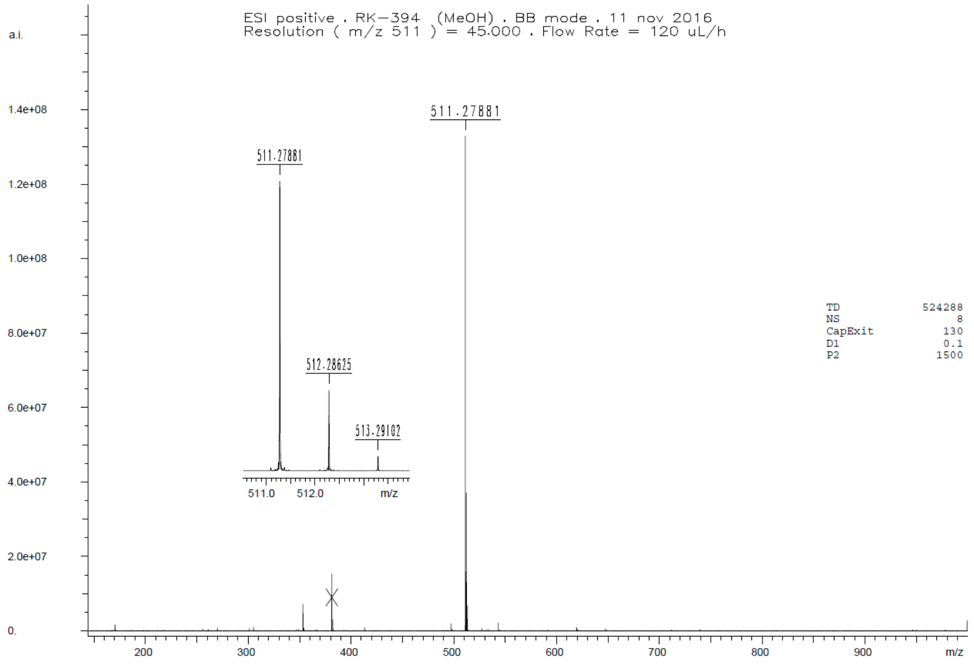

Compound 5.

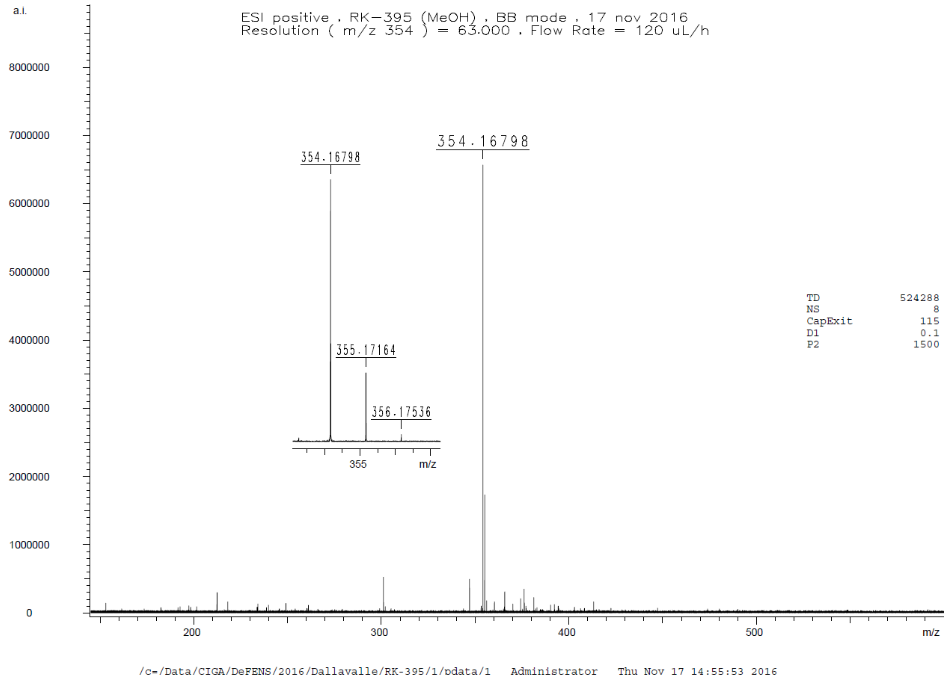

Compound 6.

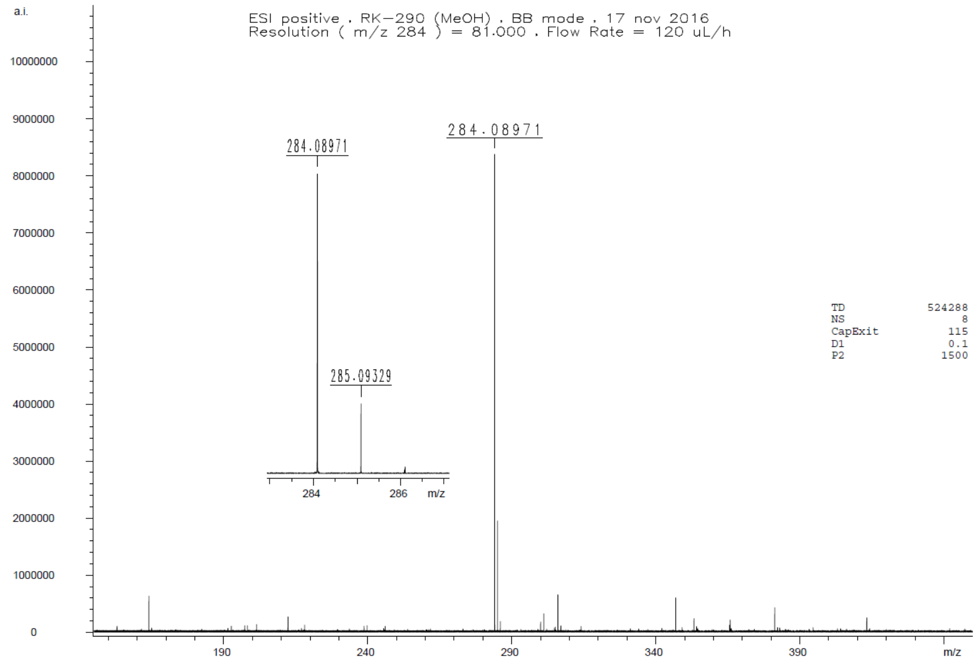

Compound 7.

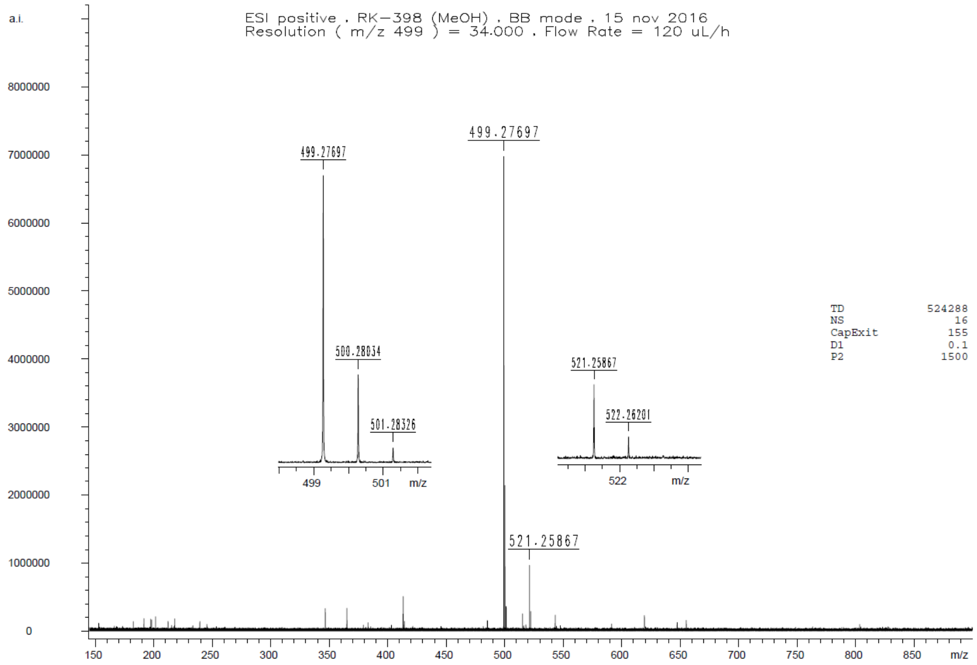

Compound 8.

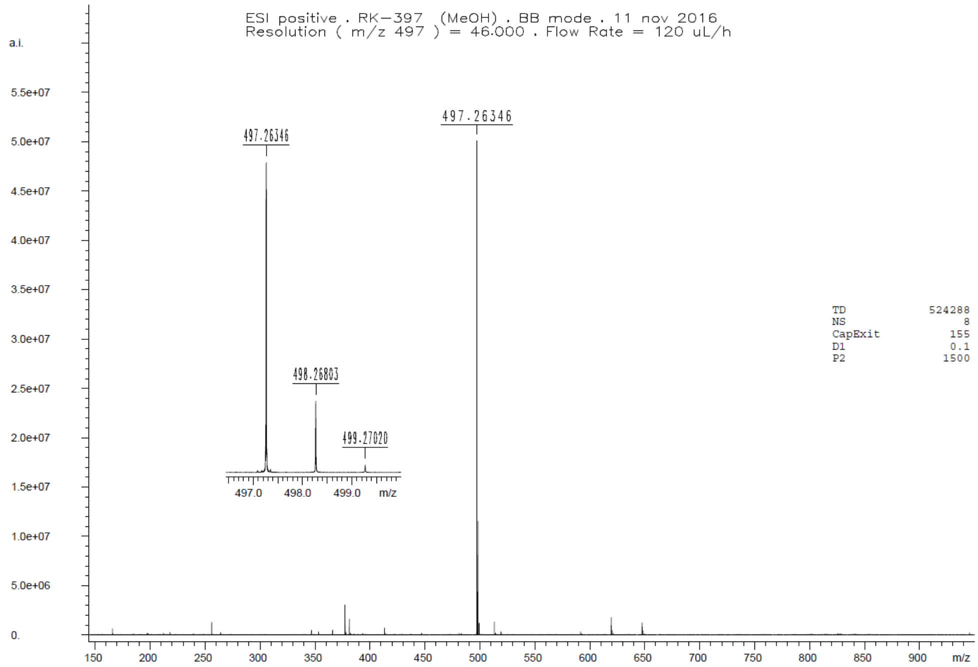

Compound 9.

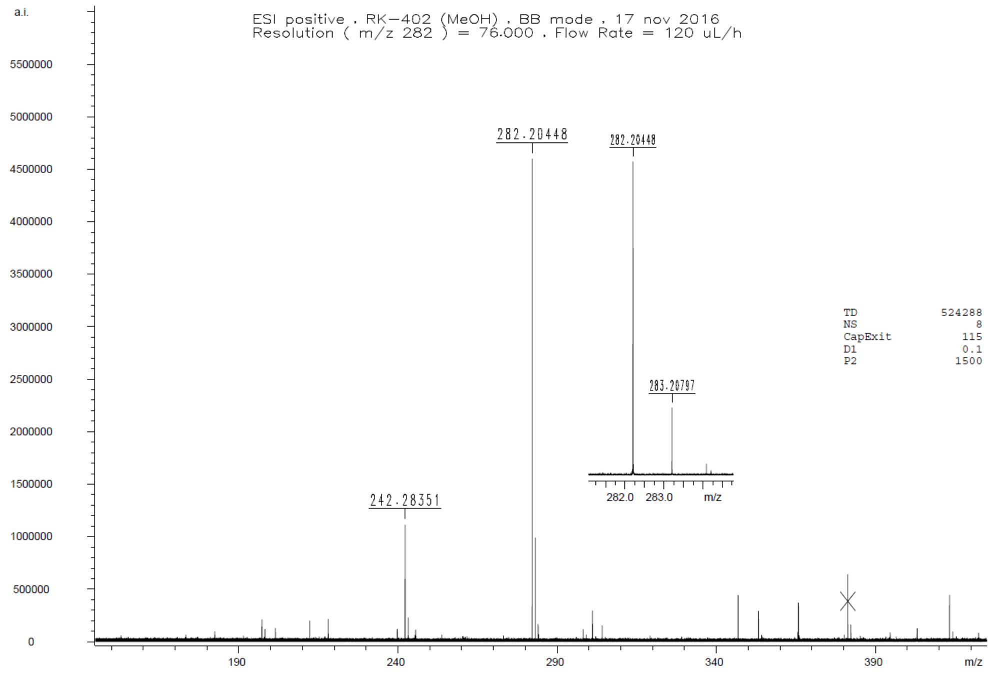

Compound 10.

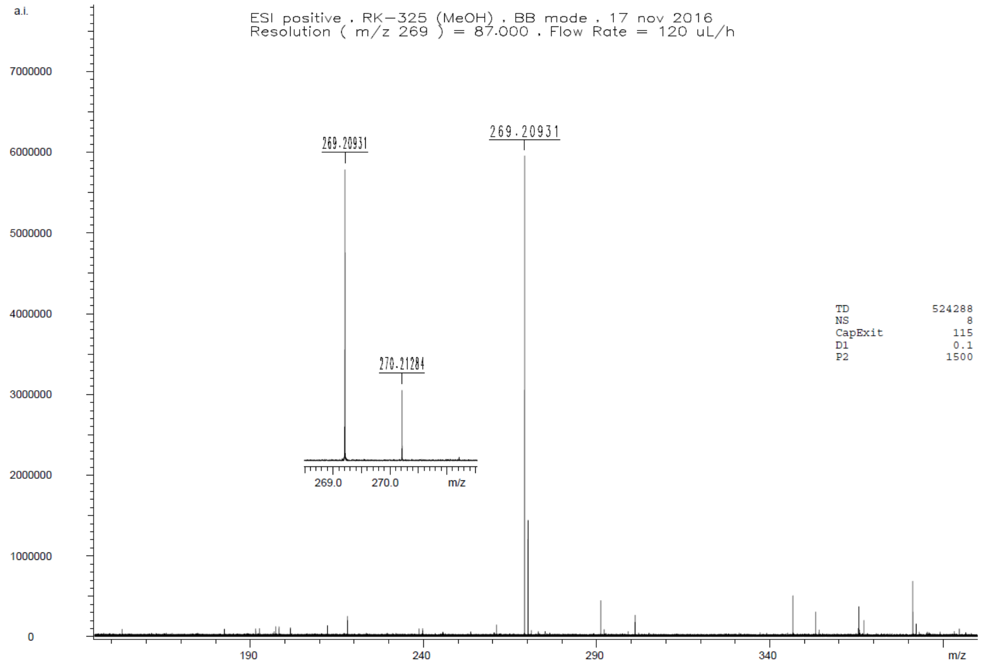

Compound 11.

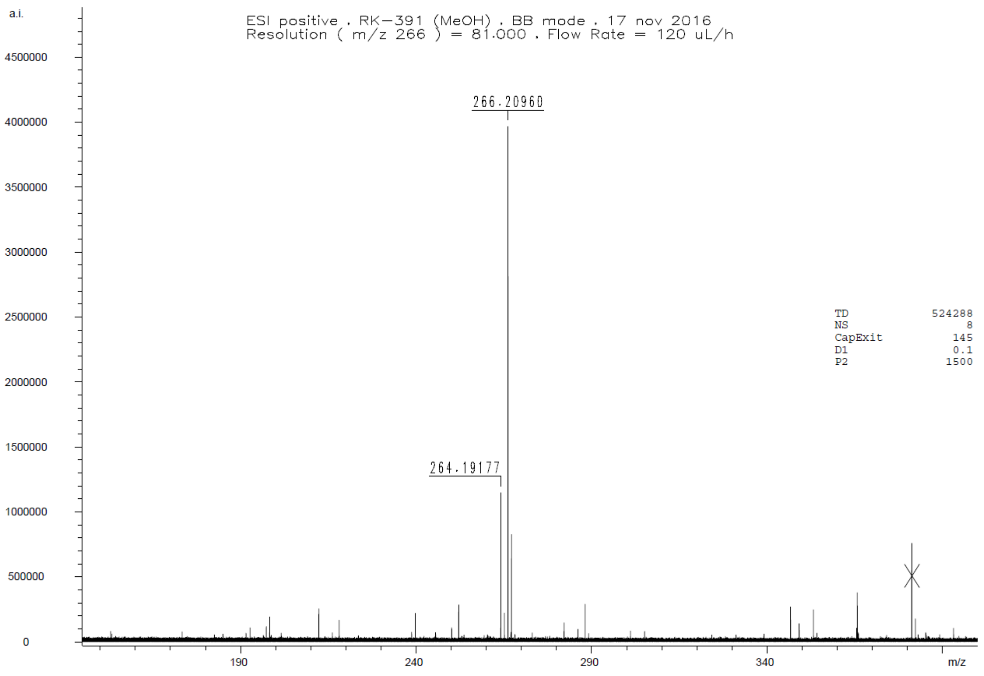

Compound S5.

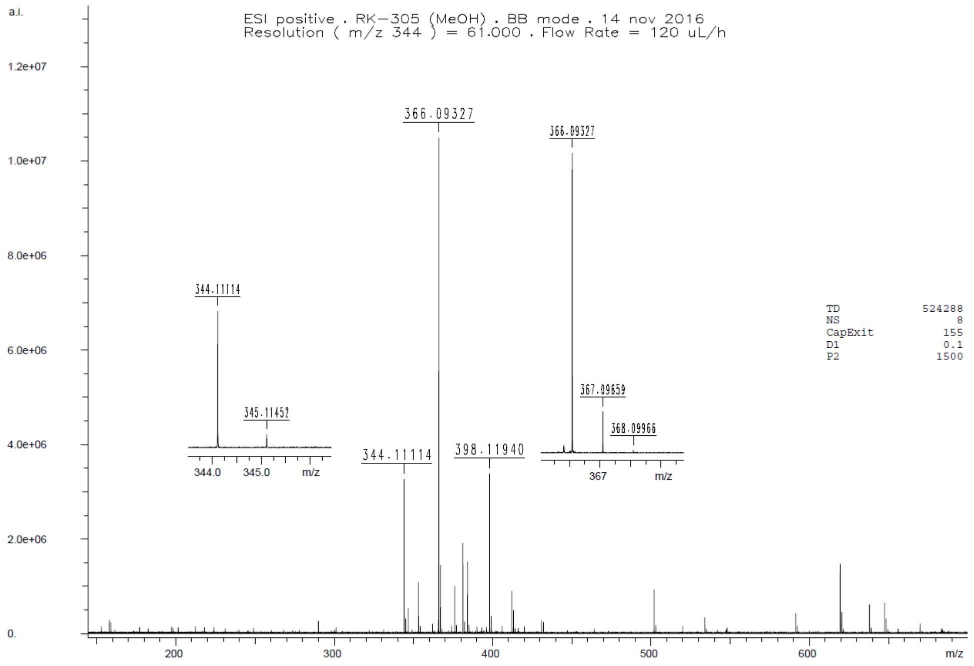

2) Materials and methods related to microbiological assays

2.1 Microbiological media and culture condition

M17 broth (Difco, Laboratories, Detroit, MI) for *Streptococcus* and *Lactococcus* species, and in MRS broth (Difco) for all *Lactobacillus*, *Pediococcus*, and *Enterococcus* species. *Staphylococcus* species were cultivated in Brain Heart Infusion broth (Difco). *Streptococcus pneumoniae* was routinely maintained in Trypticase Soy Broth (TSB) (Difco, Laboratories, Detroit, MI) with 3% (v/v) defibrinated horse blood in a 5% CO2 incubator. *Escherchia coli*, *Pseudomonas* species and *Bacillus subtilis* were cultivated in TSB (Difco, Laboratories, Detroit, MI). Cultures were incubated at 30 °C and 37 °C for mesophilic and thermophilic species, respectively.

2.2 Agar and well diffusion assay

An agar diffusion assay was carried out using *P. putida* RW10S1 (promysalin producers), and *P. stutzeri* LMG 2333 (promysalin sensitive) as reference strains[4]. Strains RW10S1 and LMG 2333 were spotted on the surface of agar Trypticase Soy Broth (TSB) (Difco, Laboratories, Detroit, MI) and incubated for 10-18 h at 30 °C. After growth, the surface of the Petri plate was exposed to a saturated atmosphere of chloroform for 10 min, and an overlay containing a suspension of 10^7 CFU/mL of the target strain in the appropriate soft agar medium was poured onto the surface. The soft agar overlay, containing agar 7.5 g/L, was prepared in TSB or M17 medium (Difco, Laboratories, Detroit, MI) for *Streptococcus thermophilus* DSM 20617^T, and in TSB or MRS medium (Difco, Laboratories, Detroit, MI) for *Pediococcus acidilactici* DSM20284^T. After solidification, the plates were incubated at the appropriate temperature for 18 h, and the presence or the absence of an inhibition halo around the *P. putida* RW10S1 was verified. For agar well diffusion assay, 10^7 CFU/ml were inoculated in melted M17 or MRS or TSB agar media (15 g/l). After the medium solidification, a well of 1 cm of diameter was created using a sterile tip and loaded with a 50 µl of DMSO of promysalin at different concentration. The plates were then incubated at the appropriate temperature for 18 h, and the presence or the absence of an inhibition halo around the wells were verified.

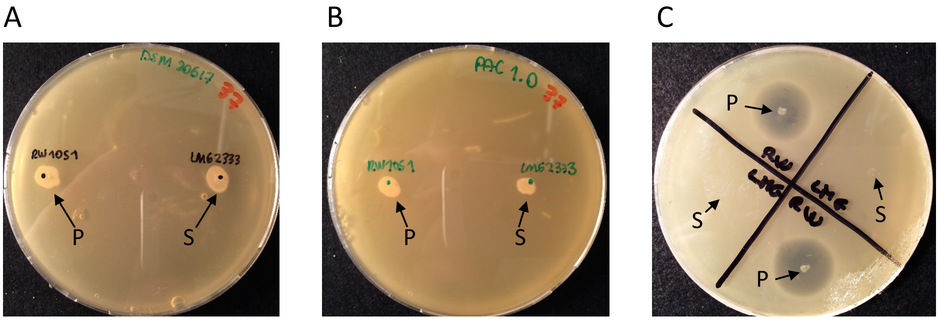

Figure S1. Agar diffusion assay carried out using P. putida RW10S1 (promysalin producers), and P. stutzeri LMG 2333 (promysalin sensitive) as reference strains 4 spotted in TSB agar. Promysalin production and activity was tested against the M17 soft agar overlay containing Streptococcus thermophilus DSM 20617T (A), and the MRS soft agar overlay containing *Pediococcus acidilactici* PAC1.0 (B) and the TSB soft agar overlay containing *Pesudomonas stutzeri* LMG 2333 (C). P, and S in the figure represent the growth of the promysalin-producer *P. putida* RW10S1, and the promysalin-sensitive strain respectively in TSB agar.

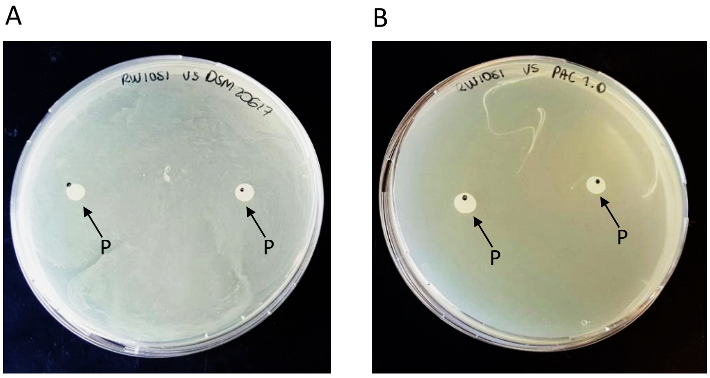

Figure S2. Agar diffusion assay carried out using *P. putida* RW10S1 (promysalin producers) as reference strains 4 spotted in TSB agar. Promysalin production and activity was tested against the TSB soft agar overlay containing *Streptococcus thermophilus* DSM 20617T (A), and *Pediococcus acidilactici* PAC1.0 (B). P in the figure represent the growth of the promysalin-producer *Pseudomonas putida* RW10S1 in TSB agar.

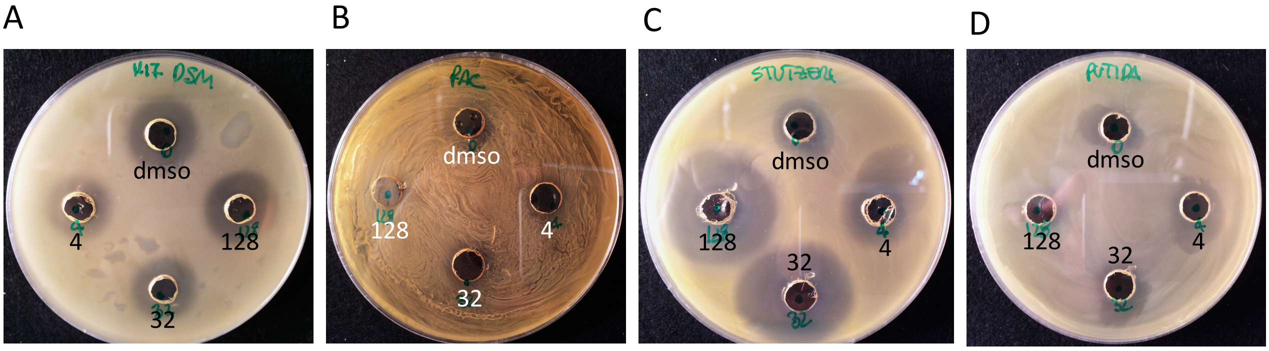

Figure S3. Agar well diffusion assay carried out using promysalin at different concentrations. Promysalin production and activity was tested against M17 or MRS or TSB agar inoculated with 107 CFU/ml of *Streptococcus thermophilus* DSM 20617T (A), *Pediococcus acidilactici* PAC1.0 (B), *Pseudomonas stutzeri* LMG 2333 (C), and the promysalin-producer (promysalin-resistant) *Pseudomonas putida* RW10S1 (D). DMSO (indicated in figure) was used in a volume equal to that used for promysalin solutions. The total amount of promysalin (μg) loaded in each well is indicated in the figure.

2.3 Evaluation of minimal inhibitory concentration (MIC) and minimal bactericidal concentration (MBC) of promysalin and its derivative analogues against Gram-positive and Gram-negative bacteria

Minimum inhibitory concentration (MIC) was determined using the broth microdilution method: after overnight growth on Mueller-Hinton broth-II (MHB-II) (Sigma-Aldrich, Milan, Italy) plates, strains were suspended in MHB-II to a standardized OD_{590nm} of 0.5. Three 10-fold dilutions were performed, and each cell suspension was inoculated in the presence of promysalin or its analogues at the indicated concentrations. Chlorhexidine digluconate (20 % w/v aqueous solution, code C9394), benzalkoniun chloride (code 12060) and surfactin (code S3523) were purchased by Sigma-Aldrich. Determination of the minimum bactericidal concentration (MBC) was performed by subculturing 10 µL from each well without visible microbial growth. After 48 hours of incubation, the promysalin or analogue dilutions yielding three colonies or less were scored as the MBC for starting inocula of 10^5 CFU/ml. The experiments were performed in triplicate. MIC and MBC were performed according to CLSI (Clinical and Laboratory Standards Institute) methods for dilution antimicrobial susceptibility tests for aerobic bacteria (approved standard, Wayne, PA, USA: CLSI; 2009).

Table S1. Minimal inhibitory concentration (MIC, µg/ml) and minimal bactericidal concentration (MBC, µg/ml) values of chlorhexidine and benzalconium chloride against Gram-negative and Gram-positive bacteria

Compound	*Pseudomonas aeruginosa* ATCC 10145		*Pseudomonas stutzeri* LMG 2333		*Pseudomonas putida* RW 10S1		*Streptococcus thermophilus* DSM20617^{T}	
	MIC	MBC	MIC	MBC	MIC	MBC	MIC	MBC
Chlohexidine	32	32	8	16	8	16	4	32
Benzalconium chloride	64	128	16	32	32	64	2	16

2.4 Flow cytometry evaluation of cell membrane damage and measurement of cFSE fluorescence cell leakage

To evaluate whether membrane damage was linked to cell leakage of intracellular components, microbial cells grown for 18 h in the appropriate medium in Petri dishes were collected and diluted in sterile filtered (0.2 µm) phosphate-buffered saline (PBS) (NaCl 8 g/L; KCl 0.2 g/L; Na_2HPO_4 1,44 g/L; KH_2PO_4 0.24 g/L; pH 7.4) to a final concentration of 10^8 events per mL. The cell suspension was diluted to 10^6 events/mL and then exposed to promysalin (100 µg/mL) or its derivative analogues (100 µg/mL), chlorhexidine (100 µg/mL) (Sigma-Aldrich) or benzalkonium chloride (100 µg/mL) (Sigma-Aldrich) at 37 °C. The cell suspension was also exposed to a DMSO control. At the time requested, a sample was collected and subjected to SYBR Green I/PI double staining and analysis by flow cytometry and, when necessary, to a standard plate count in the appropriate medium. In flow cytometry, particles/cells that pass through the beam will scatter light, which is detected as forward scatter (FSC) and side scatter (SSC). FSC correlates with cell size, cell shape and cell aggregates, whereas SSC depends on the density of the particles/cells (i.e., the number of cytoplasmic granules and membrane size). In this manner, cell populations can often be distinguished based on differences in their size and density. Cell suspensions were subjected to dual nucleic acid staining with cell permeant SYBR Green I (1X) and cell impermeant propidium iodide (PI) (5 µg/mL) (Sigma-Aldrich, Milan, Italy). SYBR Green I permeates the membrane of total cells and stains nucleic acids with green fluorescence. After incubation at room temperature for 15 min, the labeled cell suspensions were diluted to approximately 10^6 events per mL, and analyzed by flow cytometry. Cell suspensions that were prepared as described above were analyzed using a flow cytometer with the following threshold settings: FSC 5,000, SSC 4,000, and 20,000 total events collected. All parameters were collected as logarithmic signals, and a 488-nm laser was used to measure the FSC values. The rate of events in the flow was generally lower than 2,000 events/s. The obtained data were analyzed using BD AccuriTM C6 software 1.0 (BD Biosciences, Milan, Italy). Cell-membrane damage was carried out by applying double staining with SYBR Green I and PI. The SYBR Green I fluorescence intensity of stained cells was recovered in the FL1 channel (excitation 488 nm, emission filter 530/30 nm). PI fluorescence was recovered in the FL3 channel (excitation 488 nm, emission filter 670 nm long pass). PI penetrates only bacteria with damaged membranes, causing a reduction in SYBR Green I fluorescence when both dyes are present. Thus, live bacteria with intact cell membranes fluoresce bright green (defined as active fluorescent cells), bacteria with slightly damaged membranes exhibit both green and red fluorescence (defined as slightly membrane damaged cells) and cells with broken membranes fluoresce red (defined as non-active fluorescent cells) (ISO 19344:2015; IDF 232:2015). Active fluorescent cells, damaged cells and non-active fluorescent cells were electronically gated in density plots of green fluorescence (FL1) versus red (FL3) fluorescence. Green and red fluorescence allowed for optimal distinction between stained

microbial cells and instrument noise or sample background. Active fluorescent cells were gated in G1, cells with a slightly damaged membrane were gated in G2, and cells with broken membranes fluoresce red were gated in G3.

To evaluate whether membrane damage was linked to cell leakage of intracellular components, microbial cells grown for 18 h in the appropriate medium in Petri dishes were collected and diluted in PBS to a final concentration of 10^8 per mL. The obtained cell suspension was supplemented with 4 µM cFDASE (Sigma-Aldrich, Milan, Italy), which is a precursor molecule of cFSE. The suspensions were incubated for 30 min at 37 °C. During this incubation, membrane-permeating cFDASE was cleaved by intracellular esterases, and the resulting cFSE molecules were conjugated to the aliphatic amines of intracellular proteins. After centrifugation at 15,000 x *g* for 1 min and washing with PBS solution, the cells were suspended in an equal volume PBS. To ensure that unconjugated and free probes were eliminated by the cells, we periodically monitored cell fluorescence by flow cytometry as described below. The stability of the cell fluorescence was assessed; stained cells kept on ice in PBS after staining maintained a stable fluorescence, indicating that no free cFSE was inside the cells. Cell suspensions, prepared as described above and diluted to 10^6 events/mL, were analyzed using a flow cytometer with the previously described threshold settings. The cFSE fluorescence intensity of stained cells was recovered in the FL1 channel (excitation 488 nm, emission filter 530/30, provided by BD Biosciences, Milan, Italy). The cFSE-labeled cell suspension was then exposed to promysalin (100 µg/mL) or its derivative analogues (100 µg/mL), chlorhexidine (100 µg/mL) (Sigma-Aldrich) or benzalkonium chloride (100 µg/mL) (Sigma-Aldrich) at 37 °C. As a control, the cFSE-labeled cell suspension was also exposed to a volume of DMSO solvent equal to that used for promysalin and its derivative analogues. At the time point, two samples of each cell suspension were collected: i) one sample was labeled with PI as described above, incubated at room temperature for 15 min, and analyzed by flow cytometry. The second sample was used to measure cFSE-fluorescence cell leakage. In flow cytometry, density plots of cFSE-green fluorescence (FL1) and FSC allowed for optimal distinction between cFSE-stained microbial cells and instrument noise or sample background. Active cells showing only cFSE fluorescence were gated in G1, and cells with a slightly or heavily damaged membranes showing cFSE and PI fluorescence were gated in G2 and G3. Electronic gates on the green fluorescence/FSC density plot were used to select the measured bacterial concentration expressed as a % equal to the number of events in the gate divided by the total events counted. The sample for the measurement of cFSE-fluorescence cell leakage was centrifuged (13000 rpm, 2 min), and the cell-free supernatant transferred to a 96-microtiter plate for measurement of cFSE-fluorescence in a Victor 3 fluorometer (PerkinElmer). The fluorescence data were calculated as the average of three independent assays and expressed in arbitrary units of fluorescence ± the standard deviation.

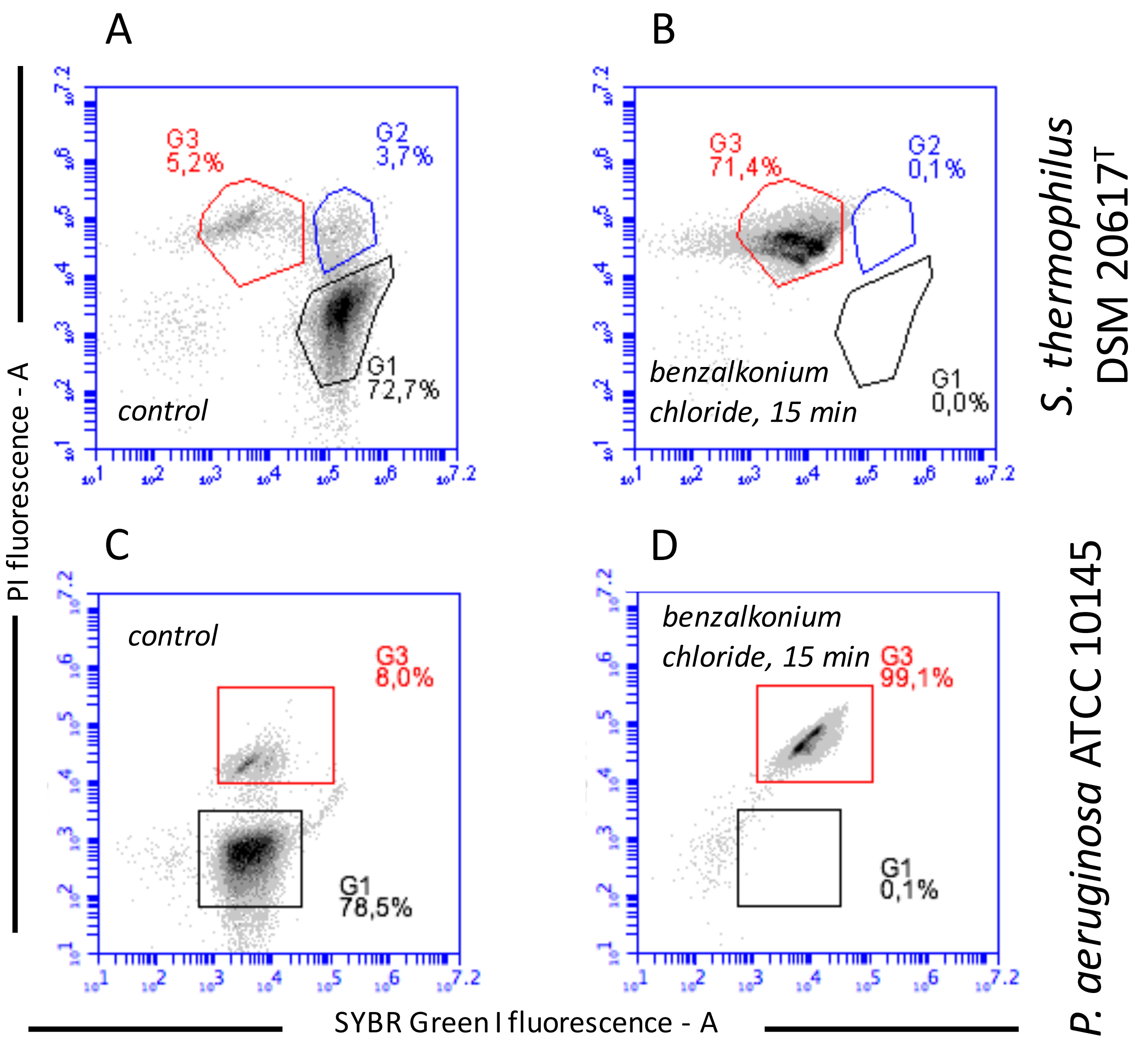

Figure S4. The effect of benzalkonium chloride on *Streptococcus thermophilus* DSM 20617^{T} and *Pseudomonas aeruginosa* ATCC 10145 cell membrane integrity. Flow cytometry density diagrams show SYBR Green I *vs* PI fluorescence of cells exposed to promysalin or benzalkonium chloride (100 and 200 µg/mL, respectively). A) and C) Cells before exposure to benzalkonium chloride (100 µg/mL). B) and D) Cells after 15 min of exposure to benzalkonium chloride. Viable cells are gated in G1. Dead cells with damaged membranes are gated in G3. The transition of cell populations from gate G1 to gate G3 is correlated to cell membrane damage.

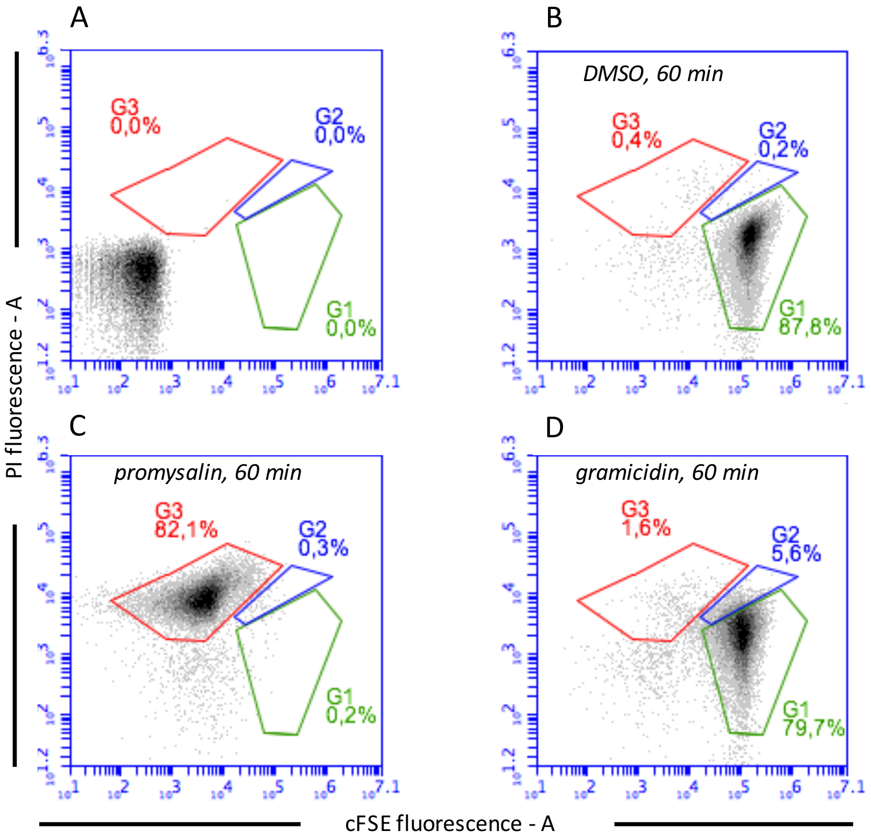

Figure S5. The effect of promysalin and gramicidin on *Streptococcus thermophilus* DSM 20617[T] cell membrane integrity. Flow cytometry density diagrams show the cFSE *vs* PI fluorescence of cells exposed to promysalin or gramicidin (100 µg/mL and 100 mM, respectively). A) Cells before exposure to antimicrobials. B) Cells after 60 min of exposure to DMSO. C) Cells after 60 min of exposure to promysalin. D) Cells after 60 min of exposure to gramicidin. Viable cells are gated in G1. Dead cells with damaged membranes are gated in G3. The transition of cell populations from gate G1 to gate G3 is correlated to cell membrane damage.

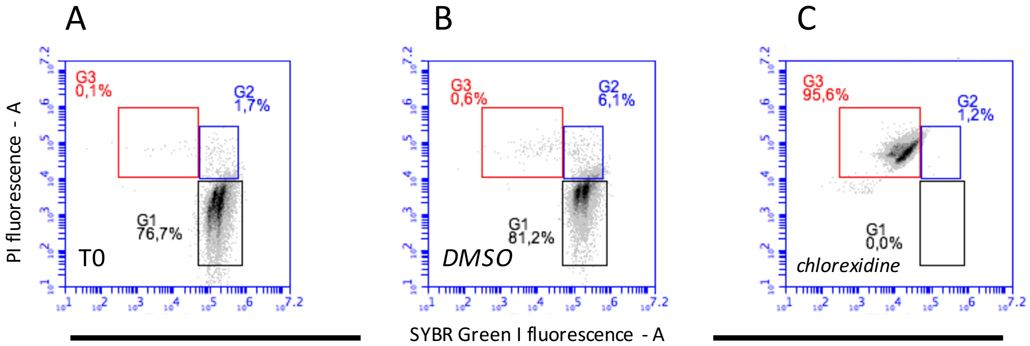

Figure S6. The effect of DMSO and chlorhexidine on *Streptococcus thermophilus* DSM 20617^{T} cell membrane integrity. Flow cytometry density diagrams show SYBR Green I *vs* PI fluorescence of cells exposed to DMSO (a volume equal to that used for promysalin and its derivative analogs), and chlorhexidine (100 µg/mL). A) Cells before exposure to antimicrobials. B) Cells after 60 min of exposure to DMSO. C) Cells after 60 min of exposure to promysalin. Viable cells are gated in G1. Dead cells with damaged membranes are gated in G3. The transition of cell populations from gate G1 to gate G3 is correlated to cell membrane damage.

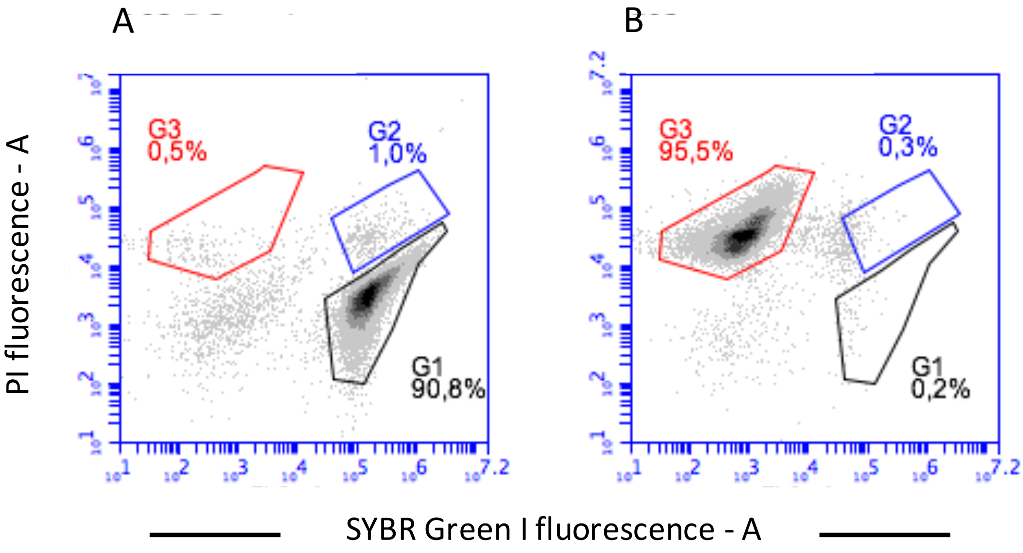

Figure S7. The effect of DMSO and promysalin on *Lactobacillus paracasei* DSM 5622^{T} cell membrane integrity. Flow cytometry density diagrams show SYBR Green I *vs* PI fluorescence of cells exposed to DMSO (a volume equal to that used for promysalin) and promysalin (100 µg/mL). A) Cells incubated 60 min at 37 °C in presence of DMSO. B) Cells after 60 min of exposure to promysalin. Viable cells are gated in G1. Dead cells with damaged membranes are gated in G3. The transition of cell populations from gate G1 to gate G3 is correlated to cell membrane damage.

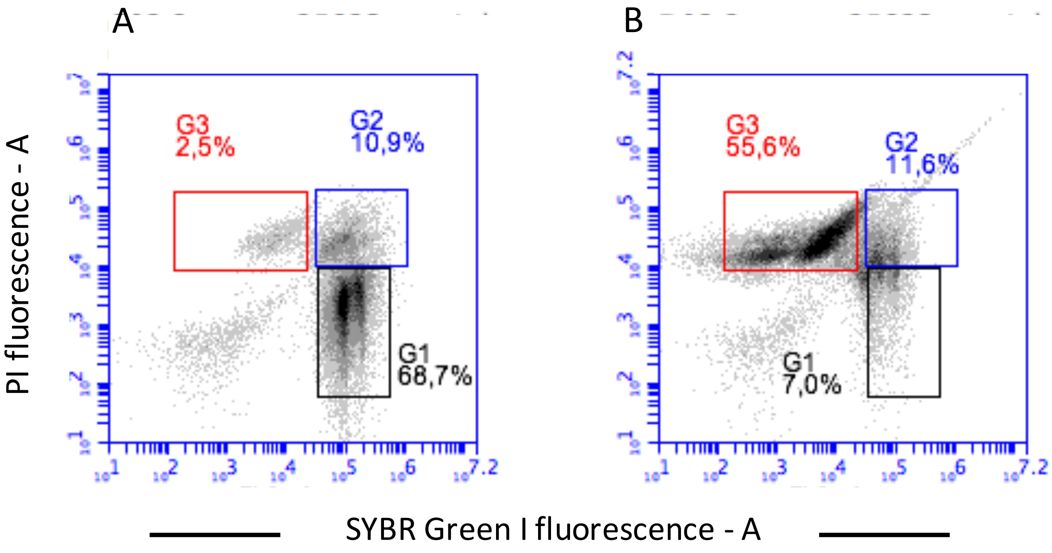

Figure S8. The effect of DMSO and promysalin on *Staphylococcus aureus* ATCC 25923 cell membrane integrity. Flow cytometry density diagrams show SYBR Green I *vs* PI fluorescence of cells exposed to DMSO (a volume equal to that used for promysalin) and promysalin (100 µg/mL). A) Cells incubated 60 min at 37 °C in presence of DMSO. B) Cells after 60 min of exposure to promysalin. Viable cells are gated in G1. Dead cells with damaged membranes are gated in G3. The transition of cell populations from gate G1 to gate G3 is correlated to cell membrane damage.

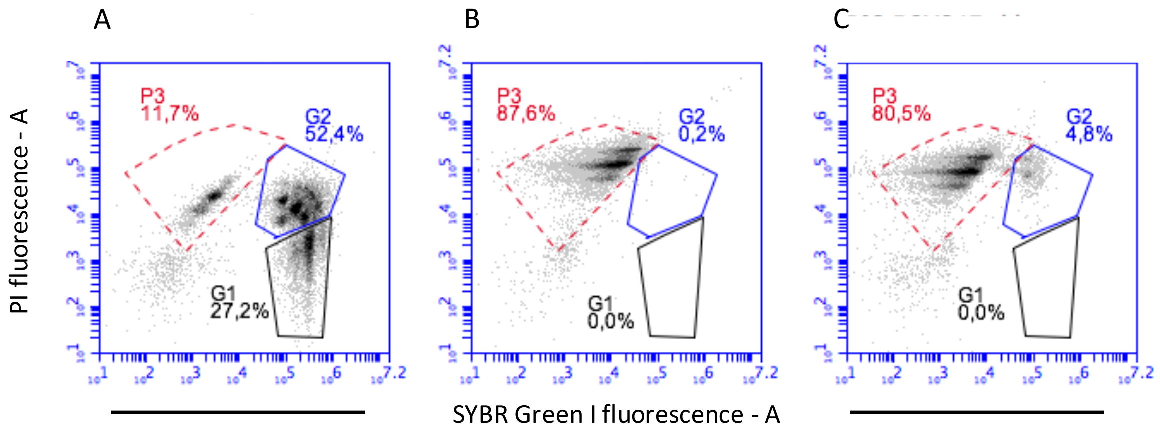

Figure S9. The effect of DMSO, promysalin and chlorhexidine on *Bacillus subtilis* DSM 347 cell membrane integrity. Flow cytometry density diagrams show SYBR Green I *vs* PI fluorescence of cells exposed to DMSO (a volume equal to that used for promysalin) and promysalin (100 µg/mL). A) Cells incubated 60 min at 37 °C in presence of DMSO. B) Cells after 60 min of exposure to promysalin. C) Cells after 60 min of exposure to chlorhexidine. Viable cells are gated in G1. Dead cells with damaged membranes are gated in G3. The transition of cell populations from gate G1 to gate G3 is correlated to cell membrane damage.

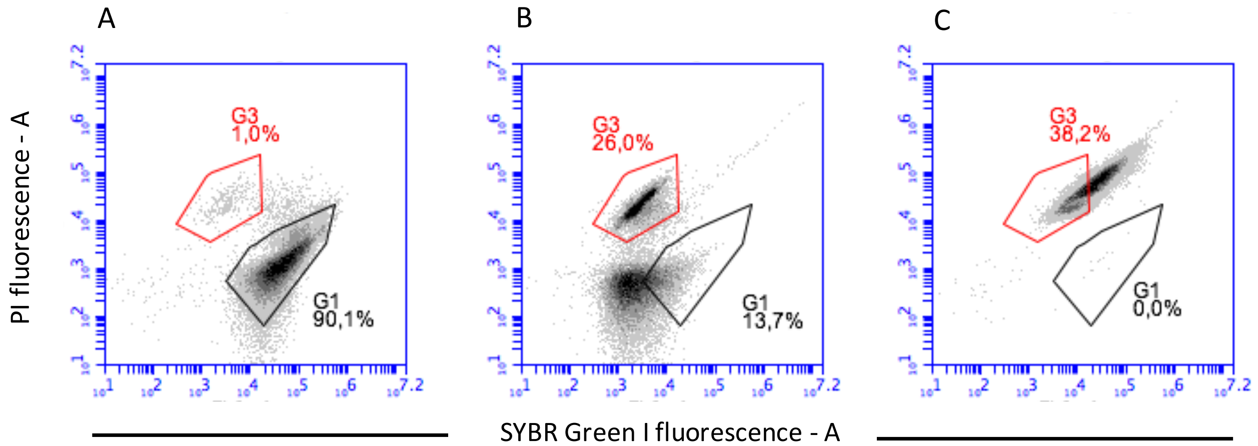

Figure S10. The effect of promysalin and chlorhexidine on *Pseudomonas stutzeri* LMG 2333 cell-membrane integrity. Flow cytometry density diagrams show SYBR Green I *vs* PI fluorescence of cells exposed to promysalin or chlorhexidine (100 µg/mL) for 1h at 37 °C. A) Cells exposed to DMSO as control; B) Cells exposed to promysalin; C) Cells exposed to chlorhexidine. Viable cells are gated in G1, Dead cells with damaged membranes are gated in G3. The transition of cell populations from gate G1 to gate G3 is correlated to cell membrane damage.

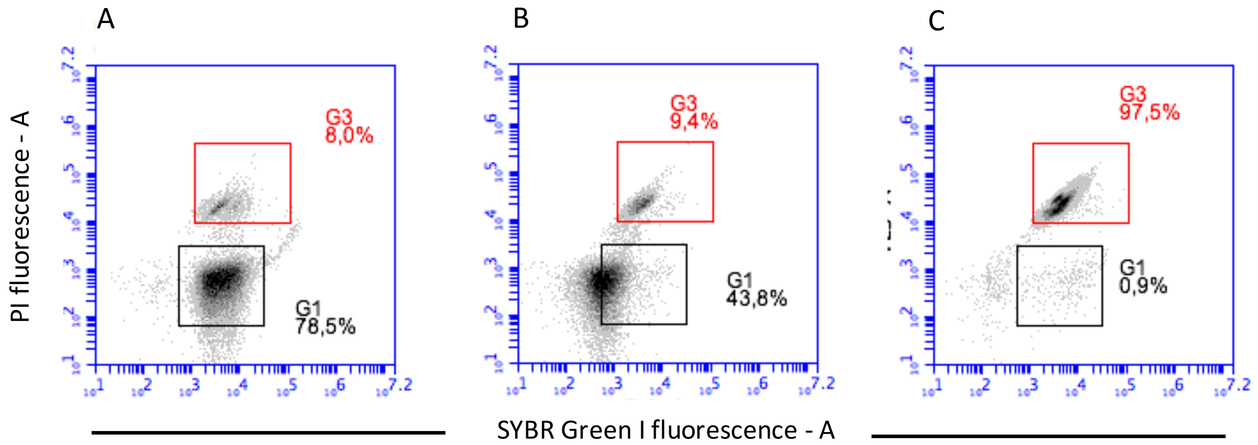

Figure S11. The effect of promysalin and chlorhexidine on *Pseudomonas aeruginosa* ATCC 10145 cell-membrane integrity. Flow cytometry density diagrams show SYBR Green I *vs* PI fluorescence of cells exposed to promysalin or chlorhexidine (100 µg/mL) for 1h at 37 °C. A) Cells exposed to DMSO as control; B) Cells exposed to promysalin; C) Cells exposed to chlorhexidine. Viable cells are gated in G1, Dead cells with damaged membranes are gated in G3. The transition of cell populations from gate G1 to gate G3 is correlated to cell membrane damage.

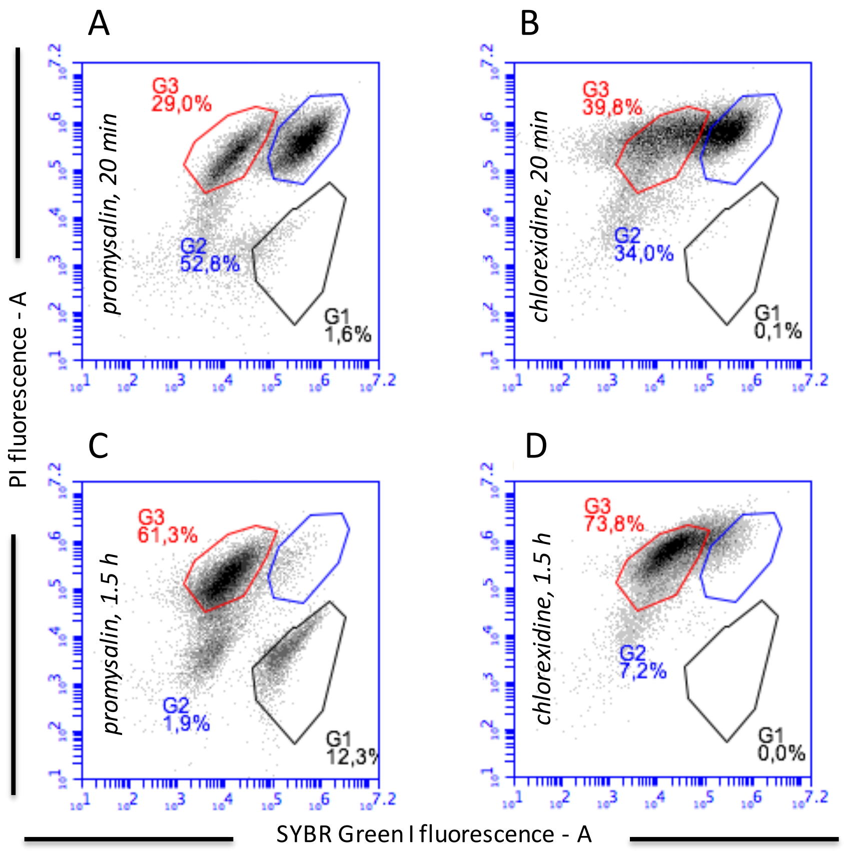

Figure S12. The effect of promysalin and chlorhexidine on *Escherichia coli* ATCC 25922 cell-membrane integrity. Flow cytometry density diagrams show SYBR Green I *vs* PI fluorescence of cells exposed to promysalin or chlorhexidine (100 and 200 µg/mL, respectively). A) and B) Cells after 20 min of exposure to antibacterials. C) and D) Cells after 1.5 h-exposure to antimicrobials. Viable cells are gated in G1, and viable cells with slightly damaged cell membranes are gated in G2. Dead cells with damaged membranes are gated in G3. The transition of cell populations from gate G1 to gate G3 is correlated to cell membrane damage.

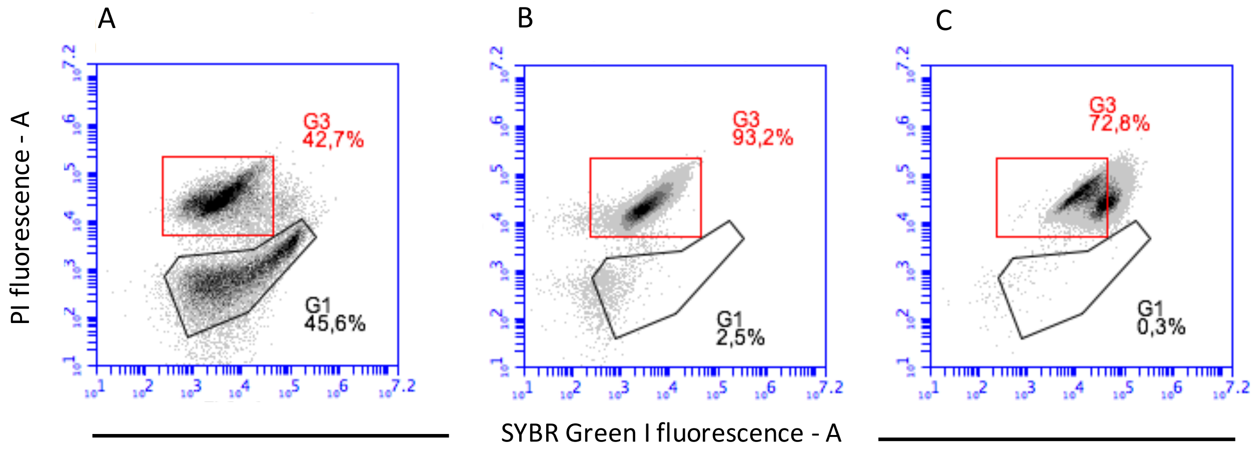

Figure S13. The effect of promysalin and chlorhexidine on *Acetobacter aceti* MIM2000/28 cell-membrane integrity. Flow cytometry density diagrams show SYBR Green I *vs* PI fluorescence of cells exposed to promysalin or chlorhexidine (100 µg/mL) for 1h at 37 °C. A) Cells exposed to DMSO as control; B) Cells exposed to promysalin; D) Cells exposed to chlorhexidine. Viable cells are gated in G1, Dead cells with damaged membranes are gated in G3. The transition of cell populations from gate G1 to gate G3 is correlated to cell membrane damage.

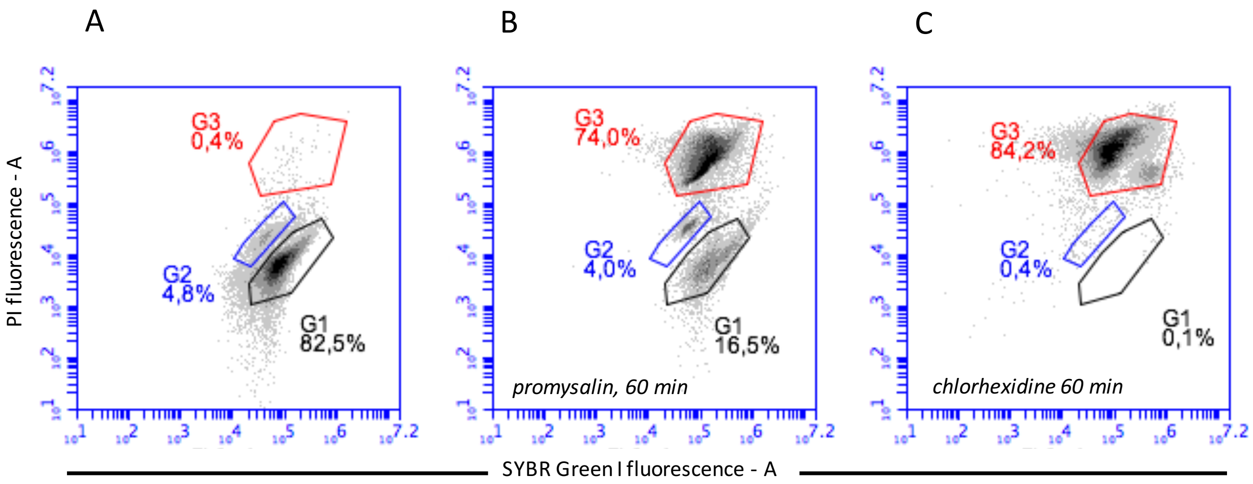

Figure S14. The effect of promysalin and chlorhexidine on *Saccharomyces cerevisiae* BC1 cell membrane integrity. Flow cytometry density diagrams show SYBR Green I *vs* PI fluorescence of cells exposed to promysalin and chlorhexidine (100 µg/mL). A) Cells before exposure to antimicrobials. B) Cells after 60 min of exposure to promysalin. C) Cells after 60 min of exposure to chlorhexidine. Viable cells are gated in G1. Dead cells with damaged membranes are gated in G3. The transition of cell populations from gate G1 to gate G3 is correlated to cell membrane damage.

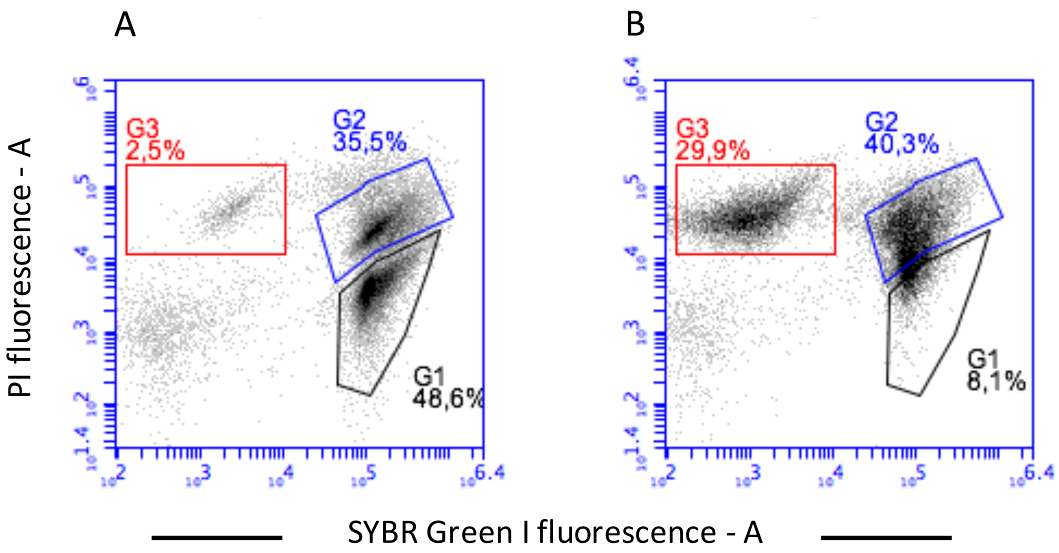

Figure S15. The effect of surfactin on *Streptococcus thermophilus* DSM 20617 cell membrane integrity. Flow cytometry density diagrams show SYBR Green I *vs* PI fluorescence of cells exposed to surfactin (200 µg/mL). A) Cells before exposure to antimicrobials. B) Cells after 60 min of exposure to surfactin. Viable cells are gated in G1. Dead cells with damaged membranes are gated in G3. The transition of cell populations from gate G1 to gate G3 is correlated to cell membrane damage.

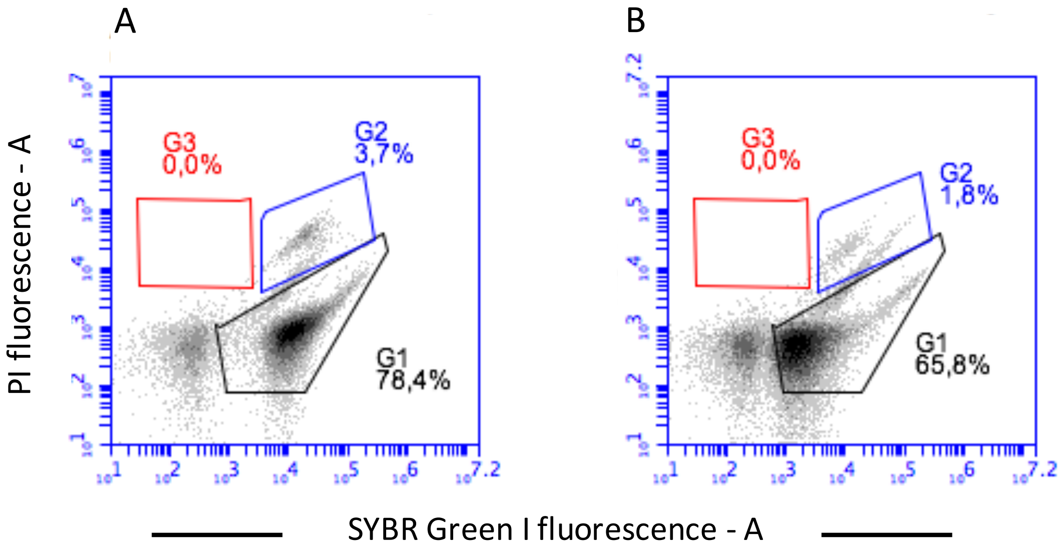

Figure S16. The effect of surfactin on *Pseudomonas aeruginosa* ATCC 10145 cell membrane integrity. Flow cytometry density diagrams show SYBR Green I *vs* PI fluorescence of cells exposed to surfactin (200 µg/mL). A) Cells before exposure to antimicrobials. B) Cells after 60 min of exposure to surfactin. Viable cells are gated in G1. Dead cells with damaged membranes are gated in G3. The transition of cell populations from gate G1 to gate G3 is correlated to cell membrane damage.

2.5 Evaluation of growth-kinetic parameters of *Saccharomyces cerevisiae* BC1 in the absence and presence of promysalin

S. cerevisiae BC1 growth was monitored in 96-well plates that were filled using an automatic liquid handling system (EpMotion, Eppendorf, Italy) to a final volume of 200 µL in the presence and absence of promysalin at different concentrations (4-128 µg/mL). A set of promysalin solutions at different concentrations in DMSO was prepared to add the same volume to each well, regardless of the final promysalin concentration. The growth of *S. cerevisiae* BC1 in the presence of promysalin was compared to its growth in the presence of a DMSO control added to the medium. Microbial growth was monitored using a spectrophotometer (MicroWave RS2, Biotek, USA) programmed for 145 readings (O.D.$_{600\ nm}$) every 10 min for 24 h at 37 °C. At the end of the incubation, the growth curve and lag time (h:min) were obtained using Gen5 software (Biotek, USA). The data were calculated as the average of three independent assays ± the standard deviation.

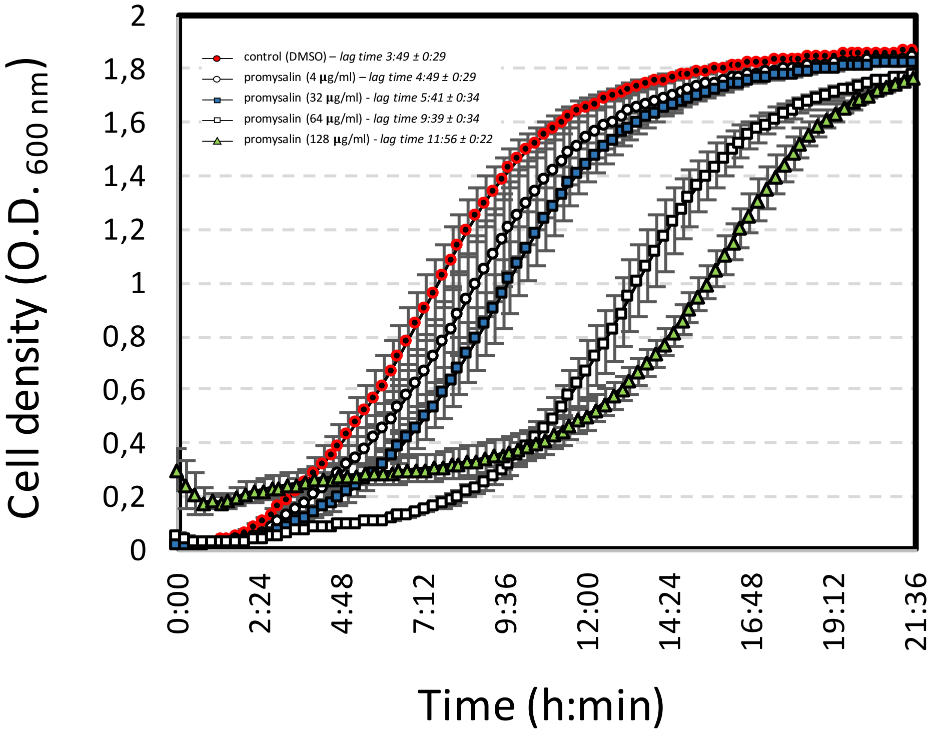

Figure S17. Growth of *Saccharomyces cerevisiae* BC1 in the absence and presence of different concentrations of promysalin. The calculated lag time (h:min) for each growth condition is indicated.

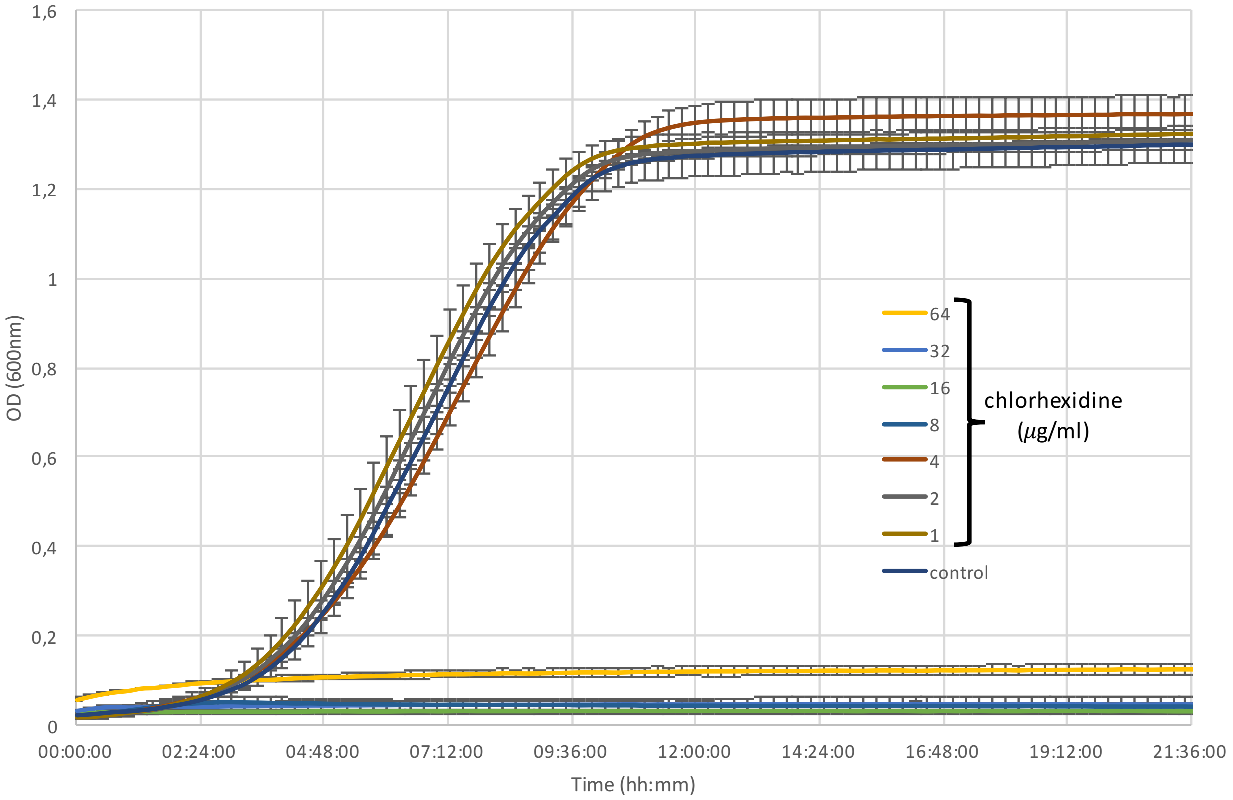

Figure S18. Growth of *Saccharomyces cerevisiae* BC1 in the absence and presence of different concentrations of chlorhexidine. Chlorhexidine concentrations (µg/ml) are indicated. The MIC for chlorhexidine was 8 µg/ml. In presence of chlorhexidine 1, 2 and 4 µg/ml, the growth curves of *S. cerevisae* were not significantly different from the growth in absence of the biocide (control).
